# Theoretical elucidation of the structure, bonding, and reactivity of the CaMn_4_O_x_ clusters in the whole Kok cycle for water oxidation embedded in the oxygen evolving center of photosystem II. New molecular and quantum insights into the mechanism of the O–O bond formation

**DOI:** 10.1007/s11120-023-01053-7

**Published:** 2023-11-09

**Authors:** Kizashi Yamaguchi, Koichi Miyagawa, Mitsuo Shoji, Takashi Kawakami, Hiroshi Isobe, Shusuke Yamanaka, Takahito Nakajima

**Affiliations:** 1 Center for Quantum Information and Quantum Biology, Osaka University, Toyonaka, Osaka 560-0043 Japan; 2 RIKEN Center for Computational Science, Kobe, Hyogo 650-0047 Japan; 3 Center of Computational Sciences, University of Tsukuba, Tsukuba, Ibaraki 305-8577 Japan; 4 Graduate School of Science, Osaka University, Toyonaka, Osaka 560-0043 Japan; 5 Research Institute for Interdisciplinary Science, and Graduate School of Natural Science and Technology, Okayama University, Okayama, 700-8530 Japan; 6 SANKEN, Osaka University, Ibaraki, Osaka 567-0047 Japan

**Keywords:** Oxyl-radical character, HOMO–LUMO mixing, HOMO-SOMO-LUMO mixing, HDFT, QM/MM, DLPNO CCSD(T_0_), High-valent metal-oxo, CaMn_4_O_5_ cluster, PSII, Water oxidation, OET, Y_z_-assisted mechanism

## Abstract

This paper reviews our historical developments of broken-symmetry (BS) and beyond BS methods that are applicable for theoretical investigations of metalloenzymes such as OEC in PSII. The BS hybrid DFT (HDFT) calculations starting from high-resolution (HR) XRD structure in the most stable S_1_ state have been performed to elucidate structure and bonding of whole possible intermediates of the CaMn_4_O_x_ cluster (**1**) in the S_*i*_ (*i* = 0 ~ 4) states of the Kok cycle. The large-scale HDFT/MM computations starting from HR XRD have been performed to elucidate biomolecular system structures which are crucial for examination of possible water inlet and proton release pathways for water oxidation in OEC of PSII. DLPNO CCSD(T_0_) computations have been performed for elucidation of scope and reliability of relative energies among the intermediates by HDFT. These computations combined with EXAFS, XRD, XFEL, and EPR experimental results have elucidated the structure, bonding, and reactivity of the key intermediates, which are indispensable for understanding and explanation of the mechanism of water oxidation in OEC of PSII. Interplay between theory and experiments have elucidated important roles of four degrees of freedom, spin, charge, orbital, and nuclear motion for understanding and explanation of the chemical reactivity of **1** embedded in protein matrix, indicating the participations of the Ca(H_2_O)_n_ ion and tyrosine(Yz)-O radical as a one-electron acceptor for the O–O bond formation. The Ca-assisted Y_z_-coupled O–O bond formation mechanisms for water oxidation are consistent with recent XES and very recent time-resolved SFX XFEL and FTIR results.

**Supplementary Information:**

The online version contains supplementary material available at 10.1007/s11120-023-01053-7.

## Introduction

Complex and well-organized biomolecular systems exhibit a huge number of important biological functions. Over past decades, functional molecular systems in biology have been investigated both experimentally and theoretically. Transition metal enzymes designed and constructed by the Nature are examples of such biological systems. One of the most important enzymatic processes on the Earth is the oxygenic photosynthesis (Govindjee and Korgmann 2004) by cyanobacteria, algae, and plant (Joliot et al. 1969; Kok et al. 1970). A lot of experimental and theoretical investigations have been performed for elucidation, understanding, and explanation of the mechanism of water oxidation in oxygen evolution complex (OEC) of photosystem II (PSII) (Cooper et al. 1978; Sauer 1980; Dismukes and Siderer 1981). Water oxidations in the native photosynthesis have been attracted great interest and importance for conversion of the Sun energy into chemical energy for sustainable life (Brudvig et al. 1989; Debus 1992; Yachandra et al. 1993). Principles elucidated for the native photosynthesis are expected to provide blueprint for artificial photosynthesis (Fujishima and Honda 1972; Tsubomura et al. 1976; Grätzel 1981; Gersten et al. 1982; Vincent et al. 1989).

Sauer wrote a review article entitled “A role for manganese in oxygen evolution in photosynthesis”, emphasizing pioneering investigations of water oxidation (Sauer 1980) initiated by Joliot and Kok and their collaborators (Joliot et al. 1969; Kok et al. 1970). Dismukes and Siderer observed the electron paramagnetic resonance (EPR) spectra of the Mn clusters in OEC of PSII (Dismukes and Siderer 1981). Mn oxides such as bi-nuclear MnO_2_Mn complex for water oxidation were attracted great interest by the late 1970s because of oil crisis. Since then, a great deal of investigations has been performed for native and artificial Mn oxide clusters (Yamaguchi et al. 1986; Pecoraro 1988; Vincent et al. 1989; Naruta et al. 1994; Limburg et al. 1999). Review articles have already been published for early studies on the natural and artificial water oxidations (Klein et al. 1993; Yachandra et al. 1996; Rüttinger and Dismukes 1997; Yagi and Kaneko 2001; McEvoy and Brudvig 2006).

Early efforts mentioned above have elucidated that the catalytic site of the native water oxidation in OEC of PSII is consisted of four Mn ions and one Ca ion, which is namely denoted as the CaMn_4_O_x_ (*x* = 5, 6) cluster (**1**) in this review. Water oxidation in Eq. (1) proceeds through five steps S_i_ (*I* = 0 ~ 4) induced by absorption of four photons (Joliot et al. 1969; Kok et al. 1970).

1 $$2{H}_{2}O+4h\nu \stackrel{Ca{Mn}_{4}{O}_{x} catalyst}{\to }{O}_{2}+4{H}^{+}+4{e}^{-}$$

Over past decades, significant efforts have been made for elucidation of the mechanism of water oxidation by **1** in OEC of PSII. To this end, the molecular structure of **1** in OEC of PSII has been investigated by the extended X-ray absorption fine structure (EXAFS) (Yano et al. 2005a; Dau et al. 2008; Grundmeier and Dau 2012), X-ray diffraction (XRD) (Zouni et al. 2001; Ferreira et al. 2004; Guskov et al. 2009), other experimental and theoretical methods (Isobe et al. 2005; Siegbahn 2006; Zein et al. 2008).

Zouni et al. first reported the XRD result of OEC of PSII (Zouni et al. 2001). Barber et al. proposed a cubane-like model for **1** (Ferreira et al. 2004). However, the precious three-dimensional structure of **1** remained to be a secret because positions of the oxygen atoms in **1** were hardly observed by XRD with the lower resolution than 2.9 Å (Guskov et al. 2009). Early theoretical computations (Zein et al. 2008; Isobe et al. 2005; Siegbahn 2006) were, respectively, performed assuming the coordination structures of amino acid residues by the EXAFS (Yano et al. 2005a), London structure (Iwata and Barber 2004) and modified London structure. Umena et al. discovered and solved a high-resolution (HR) XRD structure of **1** at 1.9 Å resolution (Umena et al. 2011). Tanaka et al. have elucidated the damage-free structure of **1** in the S_1_ state by the low-dose XRD method (Tanaka et al. 2017). Very recently, XFEL SFX experiments have elucidated the geometric structures of transient intermediates in the S_2_ and S_3_ states (Kern et al. 2018; Suga et al. 2019). Thus, the XRD experiments have provided structural foundation for elucidation of the mechanism of water oxidation in Eq. (1). However, hydrogen atoms are still invisible at current resolutions (about 1.9 Å resolution) of XRD.

The manganese ions of **1** are well known to exhibit various oxidation states for chemical reactions in chemical and biological systems (Yachandra et al. 1996; Kuzek and Pace 2001). EPR and related spectroscopies (Boussac et al. 1990; Yamauchi et al. 1997; Peloquin et al. 2000; Cox et al. 2011) have been performed to elucidate electronic and spin structures of the intermediate structures of **1** in the Kok cycle. After the discovery of the HR XRD structure (Umena et al. 2011), spectroscopic and computational studies have been performed extensively for the elucidation of the geometric, electronic, and spin structures of **1**. We have first initiated constructive collaborative work with Shen-Kamiya group, elucidating possible structures of **1** in the S_*i*_ (*i* = 1 ~ 3) states of the Kok cycle (Kanda et al. 2011b; Yamanaka et al. 2011; Isobe et al. 2012) and transition structure (S_4_) for the O–O bond formation (Saito et al. 2012; Shoji et al. 2018a, 2019b). Theoretical investigations of hydrogen bonding networks around **1** revealed by HR XRD (Umena et al. 2011) are also performed to elucidate water inlet and proton release pathways for water oxidation in Eq. (1) (Shoji et al. 2013, 2015a, b). Several review articles have already been published for elucidation of the geometric structure, electronic valence, and spin states of **1** (Lubitz et al. 2008; Yano and Yachandra 2014; Shen 2015; Shoji et al. 2019a; Yamaguchi et al. 2022a).

Over past decades, we have developed for broken-symmetry (BS) methods for theoretical investigations of complex transition metal clusters. Nowadays, BS hybrid density functional theory (HDFT) methods are handy and practical tools for investigations of large systems such as metalloenzymes. Historical developments of BS methods in our group (Yamaguchi et al. 1986, 1988a, b; Yamaguchi 1975a, 1983, 1990a) are reviewed briefly in relation to a basic principle for theoretical investigation of structure and bonding of the Earth abundant 3d transition metal oxides for oxygenation reactions such as mono-oxygenation, water oxidation.

In this review, we have mainly summarized our theoretical investigations on **1** and related model clusters by BS quantum mechanics (QM) and BS QM/molecular mechanics (MM) methods, elucidating fundamental concepts and theoretical models for understanding and explanation of structure, bonding, and reactivity of whole key intermediates structures in the S_*i*_ state (*i* = 0 ~ 3) of the Kok cycle for water oxidation. Particularly, the nature of the chemical bonds of the high-valent Mn-oxo (Mn = O) bonds (Yamaguchi et al. 1986) in the S_3_ state is examined on the basis of four degrees of freedom (spin, charge, orbital, and nuclear motion) to obtain new biomolecular and quantum insights into the mechanism of the O–O bond formation for native water oxidation. To this end, relative energies among the S_3_ intermediates by conventional HDFT are also investigated by beyond HDFT; DLPNO CCSD(T_0_). Reaction mechanisms proposed for the O–O bond formation in the final S_4_ state are revisited to obtain deep insights into very recent experimental findings obtained for the S_3_ → [S_4_] → S_0_ transition by time-resolved (TR) XFEL SFX (Bhowmick et al. 2023) and TR Fourier transform infrared experiment (FTIR) (Greife et al. 2023). Finally, the Y_z_-assisted mechanisms without formation of the Mn(V) site in OEC of PSII are discussed in relation to the reverse process, namely oxygen reduction into water molecules, by cytochrome *c* oxidase with or without Fe(V) = O site (Wikström et al. 2018; Shimada et al. 2023).

## Theoretical backgrounds

### Instability of the chemical bonds and the broken-symmetry methods

Nowadays, theoretical computation is a useful tool for investigations of photosynthesis. First of all, theoretical backgrounds are briefly referred to explanation of our way of thinking and investigation. How to understand and explain the nature of the chemical bonds of **1** embedded in protein matrix is a fundamental matter when we have initiated theoretical work as described in the proceeding of the 2009 ICCMSE conference (Yamaguchi et al. 2012). Mn oxide clusters such as **1** have been known to be challenging systems in quantum science because the 3d transition metal oxides are regarded as the so-called strongly correlated electron systems (SCES). Past decades, we have been involved to elucidate the nature of chemical bonds of open-shell systems which are suffered from the instability of the closed-shell restricted Hartree–Fock (RHF) solution with nearly degenerated orbitals, entailing broken-symmetry (BS) unrestricted Hartree–Fock (UHF) and UHF coupled cluster (CC) methods (see supporting section SI) (Yamaguchi 1980; 1990a). In early 1970s, we have initiated BS theoretical approaches to organic biradical and polyradical systems (Yamaguchi 1975a) for which electron and spin correlation effects play important roles (see Fig. S1). Several chemical indices and computational procedures such as the HOMO–LUMO mixing (Yamaguchi 1975a) have been developed and they are useful even now because of their applicability and utility of BS hybrid density functional theory (HDFT) approach to SCES (see supporting section SII). BS theoretical computations were therefore initiated to elucidate the nature of the chemical bonds of **1** in OEC of PSII on the theoretical grounds (Kanda et al. 2011a, b; Yamanaka et al. 2011).

The above theoretical view of **1** as SCES is indeed related to the nature of the chemical bonds of many other 3d transition metal oxides in general. According to our theoretical viewpoint (Yamaguchi 1990a, b), discovery of the high-*T*_c_ superconductivity of hole-doped copper oxides (Bednorz and Müller 1986) has opened the door for enormous scientific research of SCES. Interestingly, copper oxides were known to be catalytic clusters for oxygenation reactions even in 1980s (Solomon et al. 1983; Karlin et al. 1984; Kitajima et al. 1986). Therefore, common theoretical concepts and models have been indispensable for elucidation and explanation of structure, bonding, reactivity, and specific property of SCES such as copper oxides in the fields of physics (Bednorz and Müller 1986), chemistry (Yamaguchi 1983), and biology (Solomon et al. 1983). Basic theoretical models for SCES were mainly classified into the broken-symmetry (BS) single Slater determinant (Slater 1972; Yamaguchi 1975a; Döhnert and Koutecký 1980) and multi determinant (Roos 1980; Yamaguchi 1979, 1980) methods in quantum chemistry of early 1980s as shown in Fig. S1. As mentioned above, we have employed the BS method for theoretical investigation of **1**.

Too many determinants (Roos 1980; Yamaguchi 1980) are indeed necessary for the multi determinant approach to the 3d transition metal complexes. On the other hand, BS methods have been found to be practical and handy for theoretical investigation of relatively large molecular systems, metal clusters, metal oxides, etc. (Slater 1972; Yamaguchi 1979). Therefore, we have applied BS methods to elucidate structure and bonding of 3d transition metal oxides (Yamaguchi 1983, 1985, 1990a; Yamaguchi et al. 1986), elucidating that the four important concepts, (a) *the instability of the closed-shell bonds*, (b) *the orbital symmetry breaking*, (c) *appearance of local spins* (Yamaguchi 1975b), and (d) *recovery of broken symmetry *via* quantum resonance* (Yamaguchi 1990a) are guiding principles for understanding and explanation of both electronic and magnetic properties and chemical reactivity of them. Spin Hamiltonian models have been also employed to elucidate exchange coupling between local spins in metalloenzymes (Solomon et al. 1983; Yamaguchi 1988a, b). Therefore, our theoretical models are different from Hückel and extended Hückel molecular orbital models used to derive (e) *the orbital symmetry conservation rule* for no-mechanism concerted reactions (Fukui 1982; Hoffmann 1982).

### Oxy-radical character of high-valent transition metal-oxo bonds

Historically, one of the basic concepts for understanding of oxygenation reactions was the oxyl-radical character of the high-valent transition metal-oxo (M = O) bonds (Yamaguchi et al. 1986). Early discovery of radical reactivity of high-valent transition metal-oxo bonds (Groves et al. 1979; Groves and Nemo 1983) was indeed a surprise since M = O bonds were regarded as M^2+^O^2−^ responsible for the nucleophilic reactivity of the O^2−^ site. In early 1980s we have extended BS theoretical methods for M = O (M = Ti, V, Cr, Mn, Fe, Ni, Cu) to elucidate the nature of dσ-pσ and dπ-pπ bonds (Yamaguchi et al. 1986). The ab initio BS computations have elucidated that the high-valent M = O species such as [Mn(IV) = O]^2+^ and [Fe(IV) = O] ^2+^ exhibit electrophilic and/or radical reactivity in a sharp contrast to the nucleophilic character of low-valent M = O bonds: [M(m)O^2–^]^m–2^. This basic notion is very important even for understanding of water oxidation in Eq. (1).

**Fig. 1:**
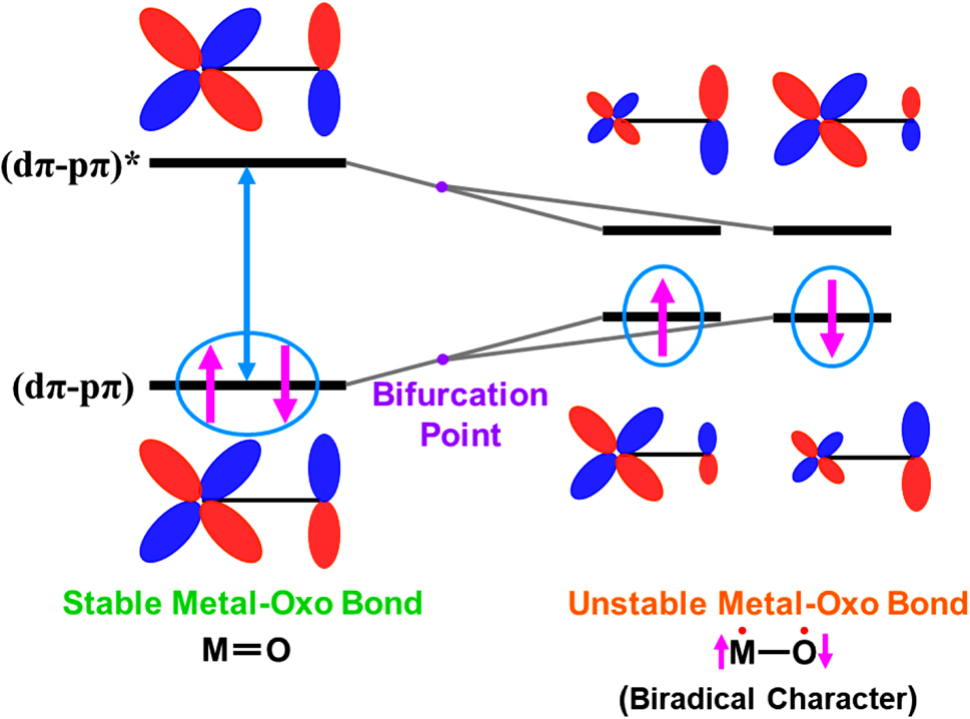
The closed-shell metal-oxo bond M = O with the large energy orbital gap between the HOMO ((dπ-pπ) bonding orbital)-LUMO ((dπ-pπ)* anti-bonding orbital). The HOMO–LUMO mixing occurs if the gap becomes smaller than the electron–electron repulsion term, providing spin polarized (SP) orbitals; namely the up-spin orbital is mainly localized on the M-site (with small tail on the O-site) and the down-spin orbital is mainly localized on the O-site (with small tail on the M site), providing the metal-oxyl-radical configuration. Indeed, the localized orbital on the O-site is similar to the singly occupied MO (SOMO) of the OH radical or atomic oxygen (O) with strong oxyl-radical reactivity. Thus, the orbital bifurcation from the closed-shell orbital to the open-shell localized orbitals provides the metal oxyl diradical state, which is often referred to as the broken-symmetry (BS) state distinct from the closed-shell non-radical state

A molecular orbital model is employed for the high-valent M = O bond as illustrated in Fig. 1, providing a closed-shell bond without the oxyl-radical character in the case of the large HOMO–LUMO gap (Holm 1987). However, the closed-shell dπ-pπ bonds of high-valent M = O species (Meunier et al. 2004; Nam 2007; Larson et al. 2020) often suffered the triplet-instability (Yamaguchi et al. 1986) because of the small HOMO–LUMO gap, giving rise to open-shell configurations with significant oxyl-radical character ·M–O·. The key concept for theoretical insight of the oxyl-radical character was the “*orbital symmetry breaking*” obtained by the HOMO–LUMO mixing (Yamaguchi 1975a), providing BS molecular orbitals localized at metal and oxygen sites, respectively, as illustrated in Fig. 1. The high-valent manganese-oxo bond exhibited significant oxyl-radical character responsible for its radical reactivity. Because of the oxyl-radical character, 1,4-diradical mechanism was indeed preferable to four-centered mechanism in the case of mono-oxygenation reaction of ethylene by Mn(IV) = O (Yamaguchi et al. 1986).

On the other hand, the metal-oxo bond M = O for early 3d transition metals such as Ti was regarded as zwitterionic (ZW) bonds such as Ti(IV)O^2−^, indicating the nucleophilic reactivity (Yamaguchi et al. 1986; Holm 1987). Therefore, the photo-induced charge-transfer (CT) from O^2−^ to Ti(IV) was necessary for generation of the ·Ti(III)-O· bond with active oxygen responsible for electrophilic or radical reactivity of the photo-catalysts. Thus, the BS method elucidated a common oxyl-radical character for radical reactivity of the excited state of early 3d transition metal-oxo bonds and the ground state of intermediate and late high-valent 3d transition metal-oxo bonds (Groves et al. 1979; Yamaguchi et al. 1986). Therefore, very important question remains “*How the nature suppresses the production of free active oxygen and oxyl-radical in water oxidation catalyzed 3d Mn oxide clusters of OEC of PSII since these species are harmful for living organisms (*Gray and Winkler 2018*)*” (one of the answers is given later).

### Four degrees of freedom for 3d metal oxides and spectroscopies

**Fig. 2:**
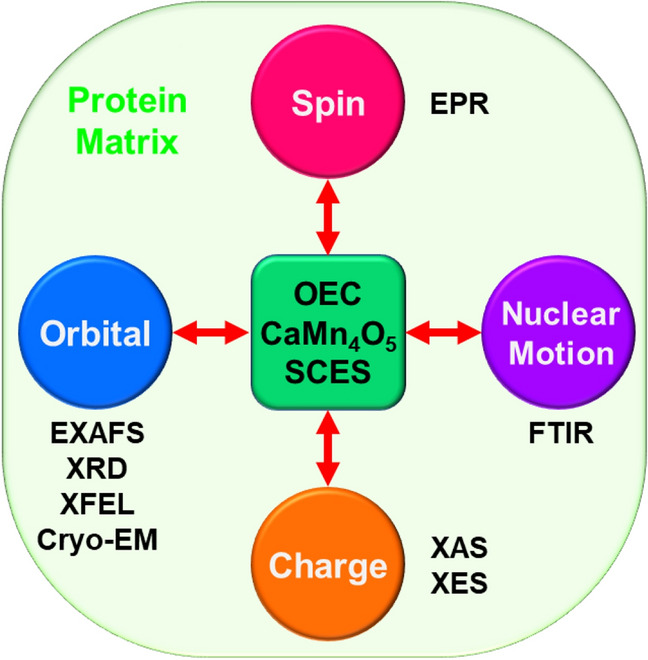
Four degrees of freedoms for transition metal oxides such as CaMn_4_O_5_ cluster with strongly correlated electron systems (SCES). Several spectroscopic methods have been applied to elucidate structure and bonding of these systems. Therefore, interplay between theory and experiment is crucial for investigations of water oxidation in oxygen evolving center (complex) (OEC) in PSII

BS computational results have elucidated four degrees of freedom for understanding of the nature of chemical bonds of 3d transition metal oxides. As mentioned above, the nature of the metal-oxo bonds [M(X) = O]^Y^ is variable with the valence state (X = II ~ V) of the metal, indicating an important role of charge degree of freedom Y = X–2. The HOMO–LUMO mixing provides the up- and down-spin densities on the metal and oxygen sites responsible for local spins with radical reactivity as shown in Fig. 1. Therefore, spin correlation diagrams (Yamaguchi 1975b; Yamaguchi et al. 2010) can be depicted for elucidation of selection rules of radical reactions. The orbital degree of freedom, namely Jahn–Teller (JT) effect of the Mn(III) and Cu(II) ions, was also important for understanding of subtle geometry changes of Mn and Cu oxides (Zein et al. 2008; Isobe et al. 2012) as shown in Fig. 2. The molecular vibration (phonon) via JT effect of the copper oxide was indeed a guiding principle for discovery of the high-*T*_c_ superconductivity (Bednorz and Müller 1986). The nature of the metal-oxo bonds is variable with the JT effect, indicating subtle changes of the M–O distances. Thus, structure, bonding, reactivity, and property of transition metal oxides such as **1** are characterized by four degrees of freedom, spin, charge, orbital, and nuclear motion (molecular vibration, motion of invisible proton, etc.) as shown in Fig. 2 (Yamaguchi 1990a; Yamaguchi et al. 2022a).

The nature of labile metal-oxo bonds is variable by strong couplings with ligands and protein matrix, exhibiting complex chemical behaviors. Various experimental methods have been applied to elucidate the nature of chemical bonds of transition metal oxides embedded in protein matrix (see later) as illustrated in Fig. 2, providing important information for the complex biological systems. Therefore, interplay between theory and experiment (Yamaguchi et al. 2022a) is crucial for understanding and explanation of chemical reactions of metal oxides with SCES in protein matrix. The basic concepts emerged by the interplay are applicable to obtain deep insights into their reaction mechanisms, providing guiding principles for design of artificial catalysts consisted of Earth abundant metals for water oxidation (Pecoraro 1988; Rüttinger and Dismukes 1997; McEvoy and Brudvig 2006; Lubitz et al. 2008; Nocera 2012).

### Early Mn oxide cluster models for water oxidation

Third wave for investigation of water oxidation by sunlight is now coming in relation to various energy and environmental sciences after the first wave of energy crisis in 1970s (Sauer 1980). As mentioned above, we can remember significant efforts for synthesis of artificial Mn complexes, and spectroscopic studies by Prof. Sauer and Dr. Yachandra and their collaborators at Berkeley (Yachandra et al. 1996). In 1980s, Prof. Tsubomura opened the research center of artificial photosynthesis at Osaka University (Tsubomura et al. 1976), where we can see the monumental card written as “A Step toward the Peace of the World “ presented by Prof. Calvin at his visit to the center; Dec 2, 1981. As mentioned above, researchers at Berkeley (Calvin 1974, 1976; Cooper et al. 1978) already started synthesis of Mn oxides model complexes at that time, suggesting formation of Mn-oxo species for oxygen evolution. Otsuji et al. also initiated experimental study on the di-μ-oxo binuclear Mn complexes (Otsuji et al. 1977). On the other hand, RIKEN at Wako has opened a research center for the oxygen evolving system of photosynthesis involving Mn complexes (Inoue et al. 1983).

From our theoretical viewpoint (Yamaguchi 1975a), molecular oxygen (O = O) in Eq. (1), together with atomic oxygen (O), were interesting species because of their ground triplet state (spin *S* = 1) (Yamaguchi et al. 1988c), suffering the triplet-instability of its closed-shell singlet configuration (Yamaguchi 1985). Hydroxyl (OH) and hydroperoxyl (OOH) obtained by hydrogen atom addition to them are free radicals with spin *S* = 1/2. Superoxide anion radical (O–O^−^) and oxygen anion radical (O^−^) generated by one-electron reduction are also *S* = 1/2 (Yamaguchi 1984). These active oxygen and oxy-radicals (O, O^−^, OH, O = O, O–O^−^, OOH, etc.) are harmful for living things such as PSII. Therefore, unveiling efforts of the secrets of effective utilization of these oxygen radicals in biological systems have been performed extensively (Solomon et al. 1983; Tabushi 1988).

The earth-abundant open-shell 3d transition metals (*S* = *n*/2; *n* = number of unpaired electrons) in metalloenzymes are often exchange-coupled with active oxygen and oxy-radicals, providing M–O–O (Yamaguchi 1983; Yamaguchi et al. 1988a, b), M = O and M–O–M (Yamaguchi et al. 1986), MO_2_M (Yamaguchi et al. 1988b), M-OOH (Yamaguchi et al. 1992). BS theoretical methods were applied to elucidate the nature of chemical bonds of these exchange-coupled systems embedded in protein matrix, providing fundamental information for experimental investigations (Calvin 1974; Otsuji et al. 1977; Sauer 1980; Tabushi 1988). Indeed, basic concepts obtained by BS computations are isolobal and isospin analogies between organic and inorganic oxides (peroxide) with common oxyl-radical character, providing guiding principles for understanding of complex mechanisms of mono- and di-oxygenations and water oxidation in PSII (Yamaguchi 1984, 1985; Yamaguchi et al. 1986, 1992, 2010, 2021) and oxygen reduction in cytochrome *c* oxidase (C*c*O) (Yoshioka et al. 2003).

**Fig. 3:**
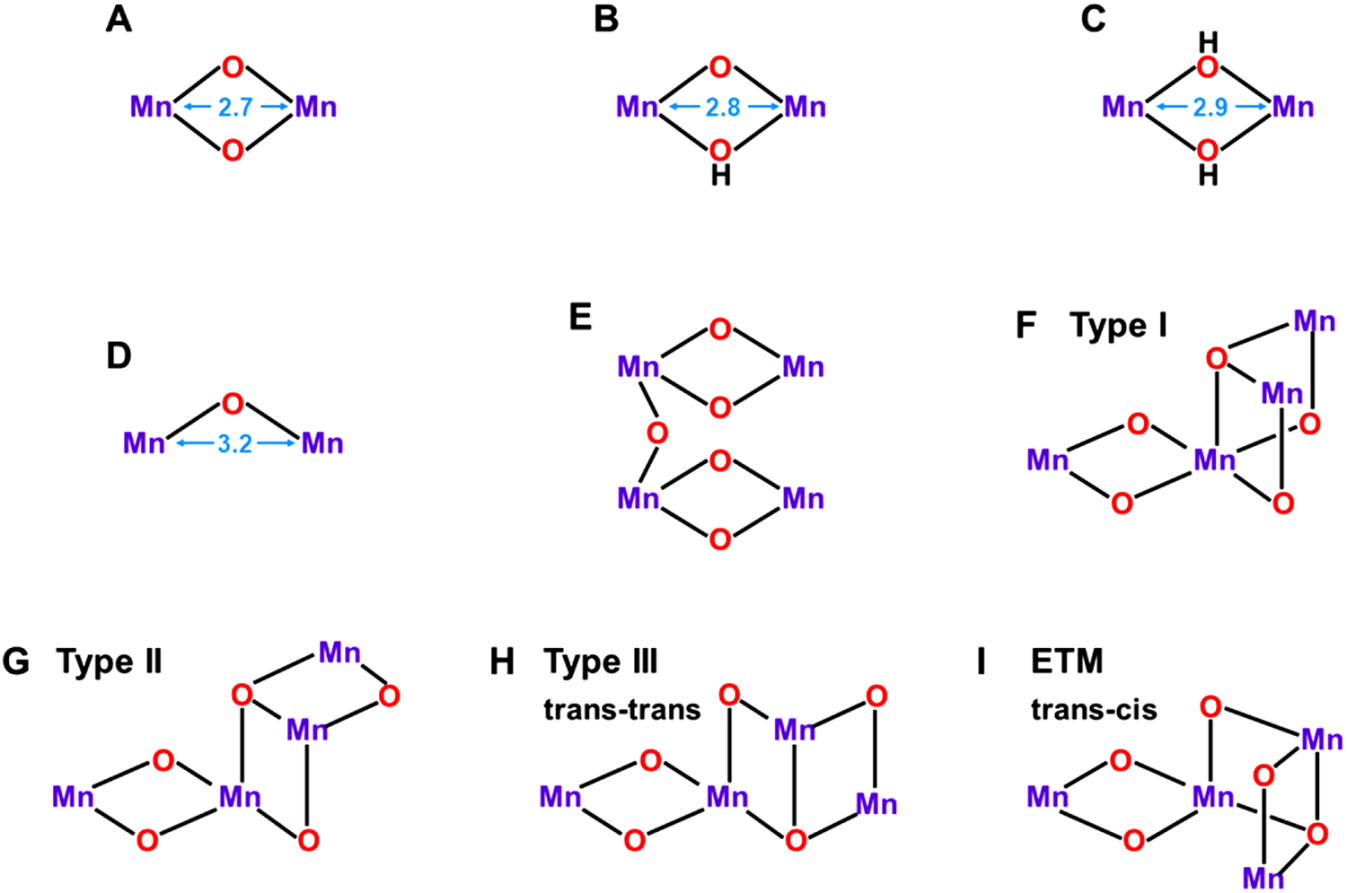
The Mn–Mn distances for the binuclear Mn complexes (**A**–**D**). These distances were used to construct the dimer of dimer model (**E**). The trimer models consisted of the three MnO_2_Mn units are also proposed (Yano et al. 2005a), providing the so-called Berkeley models F (Type I), G (Type II), and H (Type III). Extended trimer model (ETM) (Shoji et al. 2019a) based on HR XRD (Umena et al. 2011) is a trans–cis isomer as shown in I

The M = O and M–O–M units have been regarded as building blocks for construction of early Mn oxide clusters models for water oxidation in OEC of PSII. Dimerization of the formal Mn(X–2) = O^2–^ unit (X = II, III) provides binuclear Mn(X−2)(μ−O)_2_Mn(X−2) complexes as illustrated in Fig. 3A (Cooper et al. 1978; Limburg et al. 1999; Soda et al. 2000; Lubitz et al. 2008; Young et al. 2015). The oxygen dianion site(s) are often protonated, providing protonated dimer structures as shown by Mn(X–2)(μ-O)(μ-OH)Mn(X–1) in Fig. 3B and Mn(X−1)(μ−OH)_2_Mn(X–1) in Fig. 3C. On the other hand, the Mn(X–1)-(μ−O)-Mn(X–1) unit (Yamaguchi et al. 1986) is also obtained by addition of Mn(X) to Mn(X–1)-O· bond as shown in Fig. 3D. The Mn(μ−O)_2_Mn and Mn-(μ−O)-Mn complexes with octahedral ligand fields have been extensively investigated as model complexes of OEC of PSII, providing the reference Mn–Mn and Mn–O distances on the experimental grounds (Sinnecker et al. 2004).

Accumulation of oxidizing equivalents (four holes) is necessary for water oxidation Eq. (1). To this end, many Mn oxide complexes have been proposed as models for the catalytic site of OEC of PSII. For example, several three-dimensional models of the tetrahedral Mn_4_O_x_ clusters (Rüttinger and Dismukes 1997) have been formally constructed with the unit structures of Fig. 3A-D as shown in Fig. 3. EXAFS experiments (Grundmeier and Dau 2012) have been performed to elucidate geometric parameters of Mn oxide complexes for OEC of PSII. Early Berkeley model based on EXAFS by Yachandra was the dimer (2.7 Å) of dimer model in Fig. 3E (Yachandra et al. 1993). Refined EXAFS results (Yano and Yachandra 2014) have elucidated that there are two short (2.7 Å), one long (2.8 Å) and one extra-long (3.2 Å) Mn–Mn distances in the S_1_ state of OEC of PSII, providing important information for analysis of the geometric structure of OEC of PSII.

Sauer, Yachandra, and their collaborators have proposed many Mn oxide cluster models for OEC of PSII (Sauer 1980; Yachandra et al. 1996). After 2000s, they proposed the so-called Berkeley models (I, II, and III) consisted of three Mn(μ-O)_2_Mn units as shown in F, G, and H of Fig. 3 (Yano et al. 2005a). The Mn–Mn distances by EXAFS have been used for constructions of the model structures. Three type I (I_x_; *x* = a, b, c) and II (II_x_; *x* = a, b, c) structures were proposed on the basis of the different assignments of the short Mn–Mn distance (2.7 Å). The type III structure is tentatively referred to as the trans–trans conformer to express a characteristic three-dimensional (3D) conformation of H. On the other hand, the extended trimer model (ETM) structure (Shoji et al. 2019a) is essentially regarded as a 3D trans–cis conformer as shown in I of Fig. 3. The ETM structure is similar to the four Mn oxide skeleton without the Ca ion by XRD (Umena et al. 2011). Thus, three short (2.7 ~ 2.8 Å) and one long (3.3 ~ 3.4 Å) {2, 1, 1} Mn–Mn distances revealed by EXAFS (Yano and Yachandra 2014) have provided a guiding principle for understanding and explanation of Mn oxide clusters such as **1** in OEC of PSII. Full geometry optimizations starting from the Berkeley structures (Zein et al. 2008) have provided the refined but topologically the same structures, indicating the importance of trial geometries for theoretical investigations of multi-nuclear metal clusters because of difficulty of the full search of the 3D cluster structure with the absolute minimum energy.

### Right- and left-opened extended trimer models for water oxidation

**Fig. 4:**
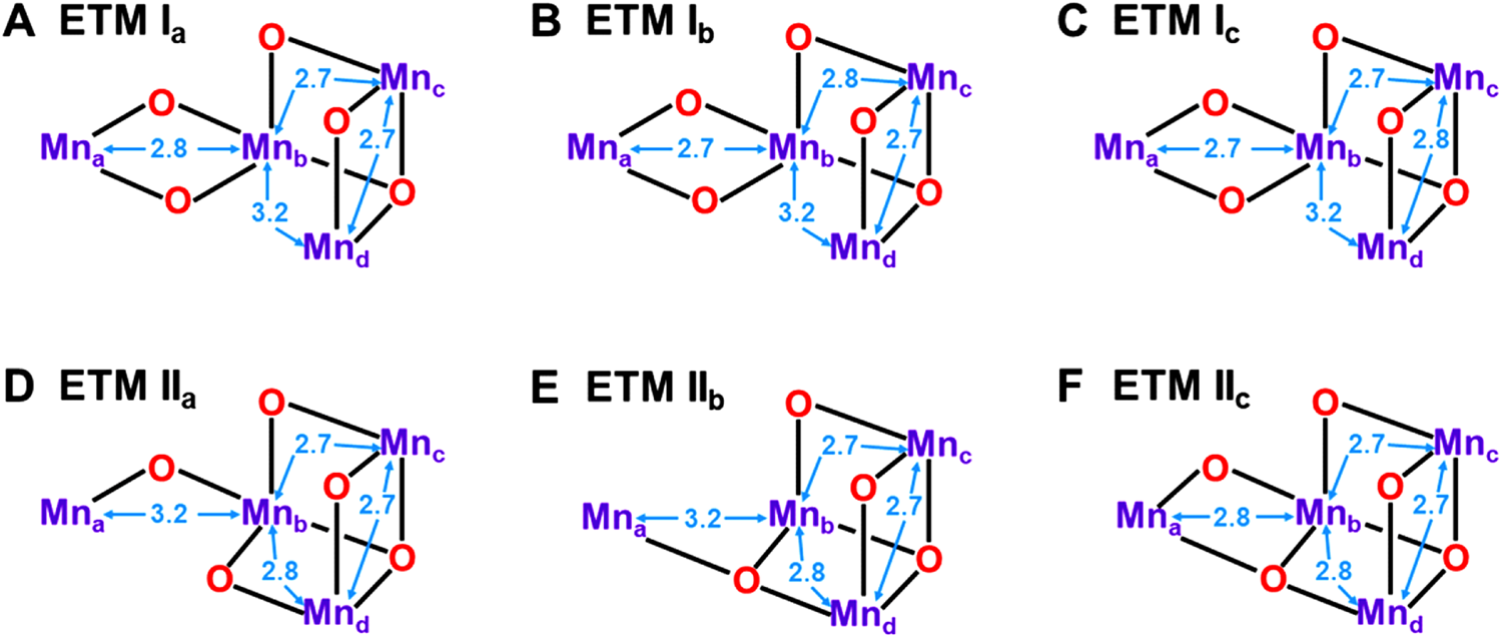
Extended trimer models for OEC of PSII. The assignments of two short and one long Mn–Mn distances in Fig. 3 provide Type I_x_ (x = a−c) and II_x_ (x = a−c) isomer structures (Shoji et al. 2019a), which are often referred to as right (open-cubane)- and left (closed-cubane)-opened structures, respectively

Discoveries of the cubane-like structures (Ferreira et al. 2004) have elucidated the global 3D structural information to modify the original Berkeley models (Yano et al. 2005a) for OEC of PSII. Type III structure, the trans–trans conformation in Fig. 3, was changed into the trans–cis conformer to obtain extended trimer models (ETM) (Shoji et al. 2019a) as illustrated in Fig. 4. From Fig. 4, the ETM I_a_ model with the right-elongated quasi-central (C_R_) structure is consistent with the HR XRD structure with the (3443) MV configuration. On the other hand, the ETM I_b_ model was consistent with the modified EXAFS model (see II_a_ with the (3344) MV configuration). Thus, the Mn_a_-O_(5)_-Mn_d_ bond was labile (Kanda et al. 2011a), providing the different topological structures depending on the motion of the O_(5)_ site. This in turn indicates that relative stabilities of these geometrical isomers seem variable, depending on coordination ligand fields and experimental conditions such as pH, etc. (Shen 2015; Yamaguchi et al. 2022a). The topological structures revealed by the HR XRD experiments (Umena et al. 2011) have provided structural foundations for understanding and explanation of early EXAFS results (Yano et al. 2005a) and other spectroscopic results (Peloquin et al. 2000; Cox et al. 2011; Pace et al. 2012).

The ETM II_a_ model in Fig. 4 was regarded as the left-opened (L) structure, where the Mn_a_-Mn_b_ distance became long (about 3.0 Å) (Guskov et al. 2009). On the other hand, the London model (Ferreira et al. 2004) was regarded as the ETM II_b_ model in Fig. 4. Thus, ETM models (Shoji et al. 2019a) are applicable to obtain a qualitative unified picture of several key structures revealed by experimental and theoretical investigations of OEC of PSII. An interesting final question is “*Where is the Ca ion in *Fig. 4* ?*” Barber, Iwata, and their collaborators have first proposed the closed-cubane structure (D, E) involving the Ca ion for active site of the CaMn_4_O_4_ as shown in Fig. 4 (Iwata and Barber 2004). On the other hand, HR XRD experiments (Umena et al. 2011) have elucidated the open-cubane structures A–C in Fig. 4. The A (ETM I_a_) model is consistent with the HR XRD structure and the A-monomer of the damage-free XRD structures in the S_1_ state (Tanaka et al. 2017). On the other hand, the B (ETM I_b_) and F (ETM II_c_) models are found to be consistent with the EXAFS results by Berkeley group (Yano and Yachandra 2014).

### Electronic and spin structures of cubane-like clusters

**Fig. 5:**
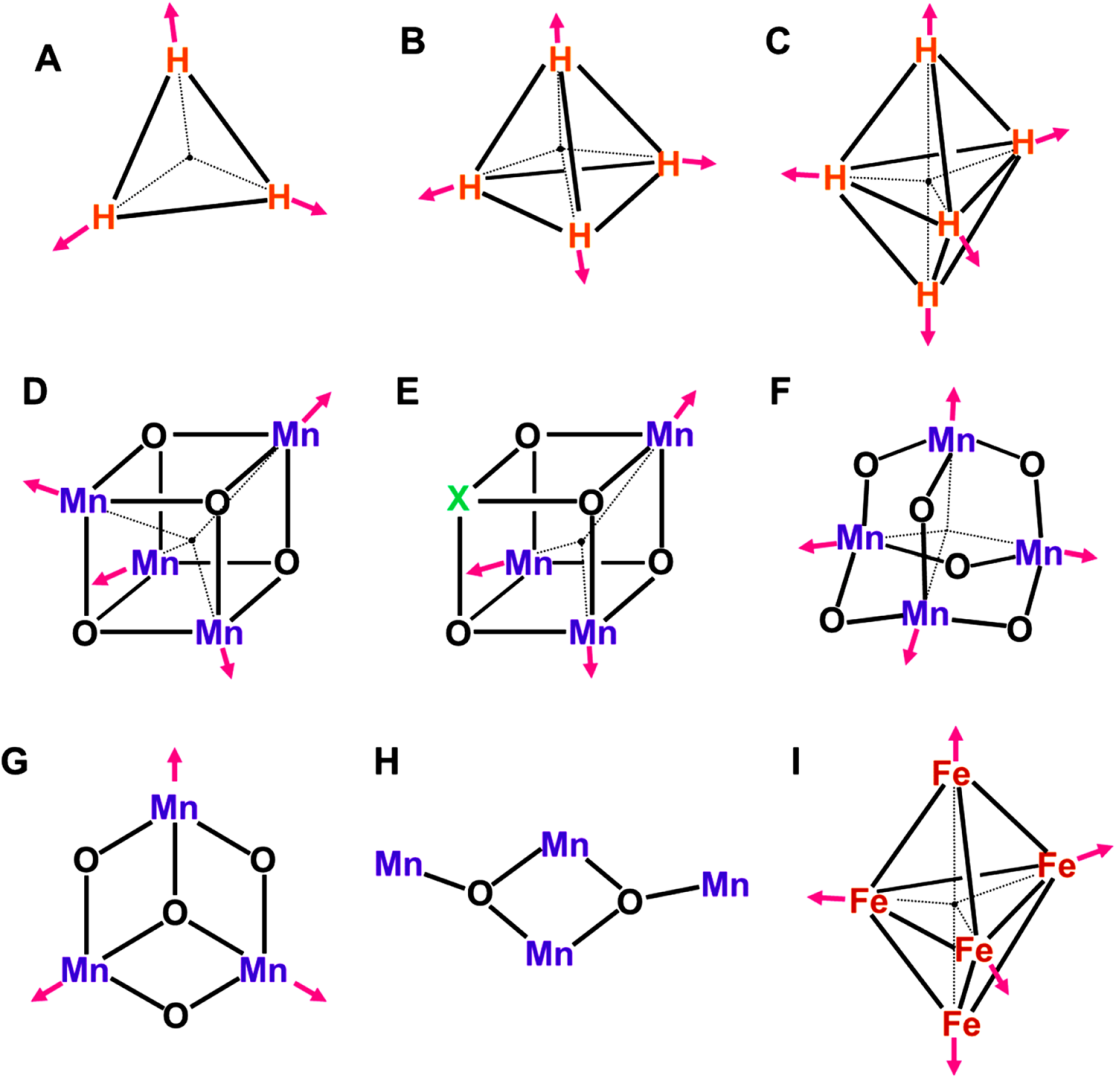
Triangular, tetrahedral, and trigonal bipyramidal clusters (**A**, **B**, **C**) with spin frustration (SF) and non-collinear spin structures (Yamaguchi 1975b). Cubane-like (**D**, **E**), adamantane-like (**F**), butterfly-type (**H**) and triangular type (**G**) Mn oxide clusters are the model complexes of catalytic sites for water oxidation. The SF trigonal bipyramidal Fe cluster (**I**) is an example of the earth-abundant artificial 3d metal (Fe) catalytic clusters for water oxidation. The SF clusters (D, G) modes are useful for qualitative understanding of artificial catalytic sites for water oxidation involved in spinel SF 3d transition metal oxides (Robinson et al. 2010) (see details in SI Part III)

A possible evolutional origin for the CaMn_4_ cluster for water oxidation has been an interesting question since the discovery of four Mn ions by EPR in OEC of PSII (Dismukes and Siderer 1981). Sauer and Yachandra have examined Mn minerals with spinel structures such as hollandite lattice, proposing many possible 3D structures of CaMn_4_ cluster (Sauer and Yachandra 2002), which involve a spin frustrated (SF) triangle Mn_3_ cluster core described by the model A in Fig. 5. The SF Mn_4_ cluster expressed by the model B in Fig. 5 (Yamaguchi 1975b) has been also an important core for explanation of peculiar magnetism of Mn spinel oxides. Thus, Mn oxides with SF structures in Fig. 5 have been accepted a double interest as the magnetic materials and effective catalysts for water oxidation.

In fact, Mn clusters have been accepted great interest in the field of the molecular magnetism in relation to quantum magnets and single molecule magnets (Hendrickson et al. 1992; Sessoli et al. 1993). Theoretically, in 1970s, triangular (Anderson 1973) and cubane-like exchange-coupled radical clusters (Yamaguchi 1975b) were already investigated as theoretical models of SF and non-collinear magnetism. The key concept to obtain the non-collinear spin structures such as triangular and tetrahedral spin structures of A, B, and C in Fig. 5 is the HOMO-SOMO-LUMO mixing because of their near orbital degeneracies (Yamaguchi 1980, 1990; Yamanaka et al. 2003a), entailing general spin orbitals (GSO) involving both up and down spin components, namely two component spinor. The non-collinear spin structures were also expected in the regular cubane structures as shown in Fig. 5D-F and the equilateral triangle in Fig. 5G. Developments of the BS computational methods for SF systems are given in the supporting section II.

As mentioned above, Hendrickson and Christou et al. have synthesized Mn oxide model complexes for OEC of PSII; for example, the Mn–O–Mn in Fig. 3, butterfly structures of the Mn_4_O_2_ complexes in Fig. 5H (Vincent et al. 1989), triangular Mn_3_O complexes in Fig. 5G (Jang et al. 1989) and distorted cubane-type Mn_4_O_3_Cl complexes in Fig. 5D (X = Cl) (Hendrickson et al. 1992). They have performed the XRD experiments of these complexes, elucidating the elongations of the Mn–O bond lengths via the Jahn–Teller (JT) effects of the Mn(III) ions. The magnetic susceptibility experiments and EPR spectroscopy have elucidated the valence states of the Mn ions and spin structures responsible for SF phenomena expected from the theoretical models in Fig. 5. Thus, early spectroscopic experiments of the Mn oxide clusters by the Hendrickson and Christou group have elucidated the important roles of spin, charge, and orbital (JT) degrees of freedom (Anderson 1973; Yamaguchi 1975b) to understand the nature of chemical bonds of strongly correlated high-valent 3d electron systems in Fig. 2.

In 1980s, Brudvig and Crabtree have already proposed the cubane-like Mn_4_O_4_ in Fig. 5E as a possible structure of the catalytic site for water oxidation (Brudvig and Crabtree 1986). They have also assumed an adamantane-like Mn_4_O_6_ clusters (Wieghardt et al. 1983) consisted of six Mn–O–Mn units as a possible precursor structure for oxygen evolution as illustrated in Fig. 5F. Dismukes et al. have extensively investigated the structure, bonding and reactivity of the Mn_4_O_4_ clusters for artificial catalysts of water oxidation, elucidating important roles of confinement materials for the stabilization of the catalytic sites (Dismukes et al. 2009). Similarly, the Mn_12_O_12_ complexes (Sessoli et al. 1993) consisted of the Mn(IV)_4_O_4_ cubane core surrounded by the Mn(III)_8_O_8_ magnetic ring have been also converted into active catalysts by electrocatalytic procedures (Yan et al. 2015; Maayan et al. 2018). Ishikawa et al. have investigated the low-spin (*S* = 0) SF trigonal bipyramid structures of M_5_ (M = Cu, Fe)-clusters in Fig. 5C and I (Ishikawa et al. 2010). Okamura et al. have discovered that Fe_5_-cluster in I of Fig. 5 becomes an active catalyst for artificial water oxidation (Okamura et al. 2016). Thus, SF 3d transition metal oxides in Fig. 5 are found to be useful catalysts for water oxidations.

In 2004, Ferreira et al. (2004) have reported the XRD structure of OEC of PSII at 3.5 Å resolution, elucidating the CaMn_4_O_4_ cluster involving the CaMn_3_O_4_ cubane core in Fig. 5D that is formally obtained by substitution of one of four Mn ions with X = Ca in a regular cubane Mn_4_O_4_ formed by the dimerization of the MnO_2_Mn unit as shown in Fig. 5E (Kanda et al. 2011a). This substitution picture has provided our notation of the CaMn_4_O_x_ structure (Sauer and Yachandra 2002) of **1** instead of the common Mn_4_CaO_x_ structure for OEC of PSII (Miyagawa et al. 2020). Therefore, the London XRD structure (Ferreira et al. 2004) involves a first biological CaMn_3_O_4_ cubane-like core, providing the structural foundation to examine possible roles of the cubane-like core and Ca ion (Isobe et al. 2005; Yamaguchi et al. 2007).

### Regular cubane-like clusters in the London XRD structure

**Fig. 6:**
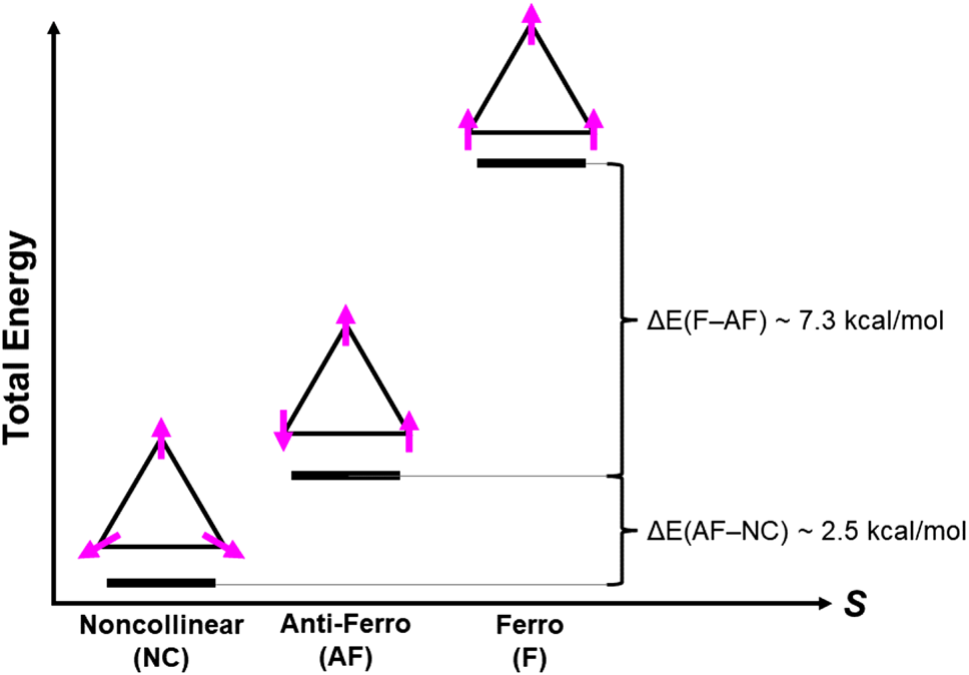
The energy levels of the low-spin non-collinear and collinear spin structures and high-spin collinear structure by hybrid DFT (Isobe et al. 2005) for the CaMn_3_O_4_ core of the CaMn_4_O_4_ cluster of the London XRD structure (Ferreira et al. 2004). The ground state was calculated to be the low-spin (*S* = 0) state. On the other hand, the CaMn_3_O_4_ core of the CaMn_4_O_5_ cluster by HR XRD (Umena et al. 2011) was high-spin, indicating the different spin state (see Fig. 9)

In 2003, we have reported both collinear and non-collinear spin structures of the regular cubane-like Mn_4_O_4_ cluster in D of Fig. 5 (Yamanaka et al. 2003b). Surprisingly, the XRD results by Barber and his collaborators (Ferreira et al. 2004) have disclosed the regular cubane-like structure CaMn_3_O_4_ (X = Ca) with almost equilateral triangular Mn_3_O_4_ fragment with about 2.7 Å Mn–Mn distance in Figs. 5A and G, indicating no significant structural distortion arising from the JT effect of the Mn(III) ion. Therefore, the Mn_3_ cluster in the CaMn_3_O_4_ cluster of the London XRD structure (Ferreira et al. 2004) was an interestingly rare example of the regular triangle with the triangle spin structure in Fig. 5G, indicating an important target for elucidation of chemical bonds by HDFT computation by general spin orbital (GSO) (Yamanaka et al. 2003b). The GSO HDFT computations (Isobe et al. 2005) have indeed provided the well-known spin alignment with 120° of spin rotation in Fig. 6 (Anderson 1973; Yamaguchi 1975b).

In 1990s, experimental studies on the cubane-like complexes have been performed in the field of the molecular magnetism. Available experiments for cubane-like Mn_4_X_4_ clusters have elucidated subtle relative stabilities between low-spin non-collinear and highest spin collinear (parallel spin) structures because of structure deformations (Yamaguchi et al. 1999) (see details in Table S1). Judging from the spin structure (Isobe et al. 2005), the London cubane-like cluster (Ferreira et al. 2004) does not involve the Mn(III) ion, indicating uniform valence states, CaMn(Y)_3_O_4_ (Y = II, IV). This means the ground state of this cluster is singlet as in the case of the cluster I in Fig. 5. Therefore, the valence state of the London structure obtained in the dark stable state was different from the mixed valence Mn(III)_2_Mn(IV)_2_ structure revealed for the valence state by EXAFS (Dau et al. 2008). The London XRD structure may be regarded as the closed-cubane CaMn(IV)_3_O_4_ structure with the (4442) valence state. However, the Mn(II) ion is hardly acceptable in the S_1_ state (Dau et al. 2008). The magnetism and chemical bonds of SF materials such as pyrochlore crystals are discussed in SI Part III in detail.

### Jahn–Teller distortions of the cubane structures

According to the guiding principle in Fig. 2, the JT effects were considered to be operative for distortions from regular triangle and regular tetrahedral structures of Mn oxides clusters involving Mn(III) ion in Fig. 5. We have performed full geometry optimizations of cubane-like CaMn(III)Mn(IV)_2_ and Mn(III)_2_Mn(IV)_2_ clusters to elucidate possible JT distorted structures (Kanda et al. 2011a). The JT effects were indeed found to be significant for Mn clusters involving the Mn(III) ion (Hendrickson et al. 1992), providing distorted cubane structures that imply conversion from the low-spin non-collinear to collinear spin state such as the highest spin state (Yamaguchi et al. 1999). BS computational results have suggested that the London structure with the non-collinear spin state in Fig. 6 does not involve the Mn(III) ion, like the L-opened S_2_ structure with the closed-cubane, CaMn(IV)_3_O_4_, in Fig. 4E.

Dau et al. and Siegbahn performed theoretical investigations of the geometric structures of the CaMn_4_O_4_ cluster (Ferreira et al. 2004) assuming the (3344) valence state revealed by the EXAFS (Yano et al. 2005a). They have obtained the open-cubane structures in accord with the Jahn–Teller effect of the Mn(III) ion (Dau et al. 2008; Siegbahn 2008). The JT effect of the Mn(III) ion, the orbital degree of freedom in Fig. 2, is certainly one of the important factors for understanding of distorted structures of Mn oxide clusters in the S_1_ state. However, the optimized geometries by them were different from the XFEL SFX structures (Suga et al. 2015) because of the wrong assumption of the (3344) valence state. Thus, the four degrees of freedom for SCES in Fig. 2 are crucial for theoretical investigations of structure and bonding of redox-active 3d metalloenzymes as shown later. In fact, the “distorted chair” structure of the HR XRD (Umena et al. 2011) suggested the JT effect of the Mn(III) ion in a sharp contrast to the regular cubane structure by the London XRD (Ferreira et al. 2004).

### Shortened distances between oxo bonds for the O–O bond formation of water oxidation

The spin-frustrated cubane structures with physical interest are also chemically important because of structural reasons for reaction fields of the O–O bond formations. Four holes are doped in catalytic clusters for water oxidation in Eq. (1) by photochemical and electrochemical methods. Therefore, distances between doped two holes are one of the important factors for the O–O bond formations, for example, as shown in the cis-cis blue dimer, *L*-Ru–O-Ru-*L* (Gersten et al. 1982), Mn-porphyrin dimer, Mn-Por-O-Por-Mn (Naruta et al. 1994) and Ru-dimer, *L*-Ru-X-Ru-*L* (X = anthracene), (Wada et al. 2000) for water oxidation. Similarly, distances between oxygen sites with doped holes (Yamaguchi et al. 2010) can become short in compact shape clusters such as dimer (McEvoy and Brudvig 2006; Kärkäs et al. 2014), triangular (Jang et al. 1989; Isobe et al. 2005), cubane (Dismukes et al. 2009), and trigonal bipyramid (Okamura et al. 2016) clusters in Fig. 5. We have examined the radical coupling (RC) mechanism (Yamaguchi et al. 2010) for the O–O bond formation, employing cubane-like clusters in Fig. 5, namely Berkeley (Yano et al. 2005a), Berlin (Guskov et al. 2009), and London (Ferreira et al. 2004) structures under the assumption of formation of high-valent Mn-oxo bonds with the oxyl-radical character Mn–O· (Yamaguchi et al. 1986) without participation of the Ca ion for the O–O bond formation (Yamaguchi et al. 2007).

The spin correlation diagrams (Yamaguchi et al. 2010) are useful for derivations of selection rules for radical coupling reactions by the cluster catalysts examined above. The oxygen radical pair of [Mn–O· …·O-Mn] becomes singlet or triplet, depending on total spin structures of Mn oxide clusters. These pairs are referred to as the local singlet diradical (LSD; ↑↓) and local triplet diradical (LTD; ↑↑) configurations, respectively (Yamaguchi et al. 2010, 2012; Tanaka et al. 2012). The O–O bond formation is facile for LSD, whereas spin inversion from LTD to LSD is necessary for RC. The situation is the same for hole-doped single molecule magnet Mn_12_O_12_(H_2_O)_4_ that has been found to become a catalyst for water oxidation (Yan et al. 2015; Maayan et al. 2018) via the L-opened transition structure for the O–O bond formation because of its rigid structure (Yamaguchi et al. 2021). Interplay between theory and experiment have provided a guiding principle based on the RC mechanism for design of structure, bonding, and reactive sites of effective artificial catalysts consisted of 3d transition metal oxides belonging to SCES in Fig. 2.

### Reduction of the oxyl-radical character by participation of the Ca(H_2_O)_n_ ion

BS computations (Yamaguchi et al. 1986) have elucidated different electronic characters between high-valent 3d metal-oxo and 4d (5d) metal-oxo bonds. The high-valent 4d (5d) transition metal M = O bonds usually exhibit large HOMO–LUMO gaps in Fig. 1, indicating no significant oxyl-radical character. The high-valent M(V) = O bonds (M = Ru, Ir, etc.) have lower-lying LUMO (Concepcion et al. 2009; Kärkäs et al. 2014) responsible for electrophilic attack by water (or hydroxy anion) group, undergoing the O–OH bond formation:

2 $${\text{M}}\left( {\text{V}} \right) = {\text{O }} + {\text{ H}}_{2} {\text{O }} + {\text{ B}} \to {\text{M}}\left( {{\text{III}}} \right) - {\text{O}} - {\text{OH }} + {\text{ BH}}^+$$

where B denotes a base for proton trapping. This type of the reaction mode with the two-electron V-III valence transition at a high-valent M site is often referred to as the acid–base (AB) mechanism. Iwata and Barber have also proposed the AB mechanism for water oxidation (Iwata and Barber 2004) on the basis of their London model (Ferreira et al. 2004). Sproviero et al. have also proposed the same mechanism based on their QM/MM computational results starting from the London model (Sproviero et al. 2008; Vinyard et al. 2015). However, Siegbahn has presented a DFT computational result against the O-OH bond formation for water oxidation (Siegbahn 2017).

Our BS computations have elucidated that the Ca ion, a Lewis acid, play important role for reduction of the oxyl-radical character of the high-valent Mn(V) = O bond; namely Mn(X–1)-O· + Ca(II) → Mn(X) = O…Ca(II) (Yamaguchi et al. 2007). We have therefore proposed the Ca-assisted O-OH bond formation under the assumption of reduction of the oxyl-radical character by the participation of the Ca(H_2_O)_n_. The Ca cubane structure in Fig. 5E is very important because of the coordination structure of the high-valent Mn = O bond to the Ca(II) ion with water molecules in Eq. (3) (Yamaguchi et al. 2007).

3 $${\text{Mn}}\left( {\text{V}} \right) = {\text{O}} \ldots {\text{Ca}}\left( {{\text{H}}_{2} {\text{O}}} \right)_{\text{n}} + {\text{ H}}_{2} {\text{O }} + {\text{ B}} \to {\text{M}}\left( {{\text{III}}} \right) - {\text{O}} - {\text{OH}} \ldots {\text{Ca}}\left( {{\text{H}}_{2} {\text{O}}} \right)_{\text{n}} + {\text{ BH}}^+$$

Participation of the Ca ion may indicate that too much oxyl-radical character of the high-valent Mn = O bond might be harmful for living things (Gray and Winkler 2018). As shown later, the high-valent Mn = O bond formed in the S_3_ state is indeed coordinated to the Ca ion (Kern et al. 2018; Suga et al. 2019), supporting our early insight.

Thus, before the discovery (Umena et al. 2011) of the HR XRD structure of **1**, fundamental concepts and computational methods in Fig. 2 have been developed for elucidation of the nature of chemical bonds of high-valent transition metal oxides in general (see supporting material SI Part III). Therefore, after the discovery, the prepared concepts and methods for redox-active SCES have been successfully applied to investigate the catalytic water oxidation by **1** (Kanda et al. 2011a; Yamanaka et al. 2011; Isobe et al. 2012), providing molecular and quantum insights into the O–O bond formation in OEC of PSII.

## Structure and bonding of possible intermediates in the Kok cycle

### High-resolution XRD structure of the CaMn_4_O_5_ cluster

**Fig. 7:**
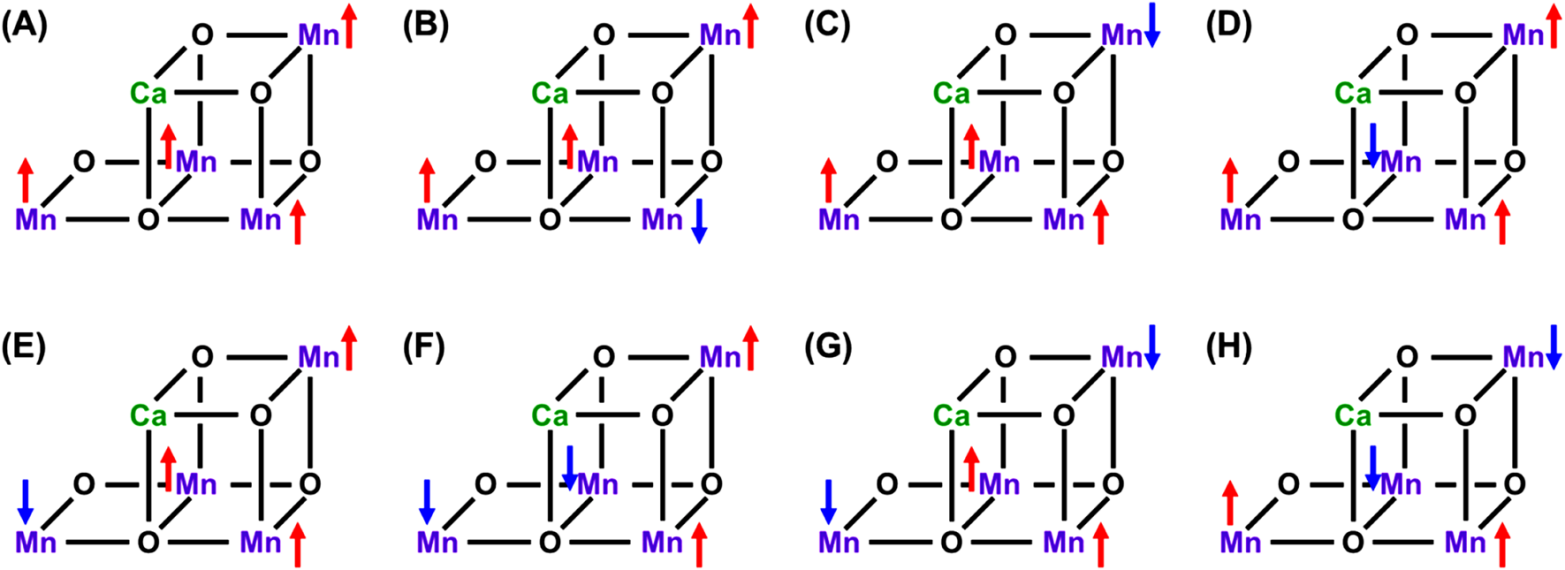
Eight spin structures (**A**–**H**) for the CaMn_4_O_5_ cluster for OEC of PSII (Kanda et al. 2011b)

In 2011, Umena et al. have reported the HR XRD structure (Umena et al. 2011) for crystals of *Thermosynechococcus vulcanus* (cyanobacteria) obtained by the elaborated chemical procedure of Keisuke Kawakami et al. (unpublished yet). As mentioned above, the HR XRD for dark stable S_1_ state has elucidated the three-dimensional “*distorted chair-like*” structure of the CaMn_4_O_5_ cluster (**1**) in OEC of PSII. The distorted open-cubane structure has been regarded as a MV complex with the JT distortion of the Mn(III) ion (Kanda et al. 2011a), for which four degrees of freedom are essential from our theoretical viewpoint as illustrated in Fig. 2. Therefore, first of all, we have initiated theoretical investigation of spin degree of freedom of **1**. Judging from the distorted chair-like structure of **1**, the non-collinear spin state of the regular cubane in the London model (Ferreira et al. 2004) was converted into the collinear spin state via Jahn–Teller effects, indicating the applicability of the conventional BS up- and down-spin structure models. Eight different axial (collinear) spin structures expressed by the Mn_1_Mn_2_Mn_3_Mn_4_ alignments were indeed feasible for **1** in OEC of PSII at the BS level of computational model as shown in Fig. 7 (Kanda et al. 2011b).

**Fig. 8:**
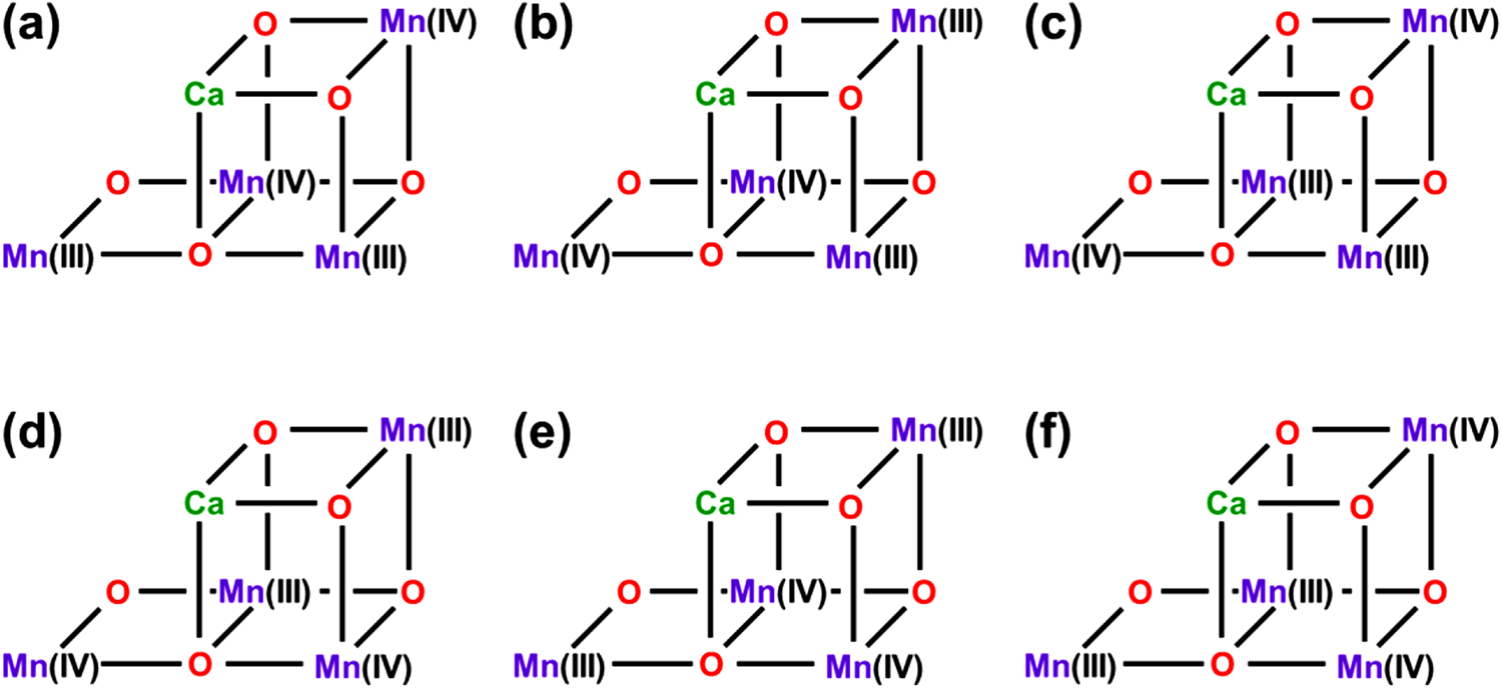
Six mixed-valence (MV) configurations of the Mn(III)_2_Mn(IV)_2_ high-oxidation S_1_ state of the CaMn_4_O_5_ cluster in OEC of PSII (Yamanaka et al. 2012) Fig. 9 Energy levels (cm−1) of the total 48 (= 8 ×6) spin-charge configurations in the S1 state of the CaMn4O5 cluster by UB3LYP computations, providing the ground and lower-lying states (Yamanaka et al. 2012)

Concerning with the charge degree of freedom in Fig. 2, the high-oxidation scenario (Yachandra et al. 1996) was assumed for the S_1_ state of the Kok cycle instead of the low-oxidation scenario (Pace et al. 2012), providing the mixed-valence (MV) configuration Ca(II)Mn(III)_2_Mn(IV)_2_ of **1**. Six different MV configurations are feasible for **1** under the high-oxidation assumption at the BS level of computational model as shown in Fig. 8. Therefore, the 8 × 6 = 48 different BS configurations were possible from spin and charge degrees of freedom in the S_1_ state of OEC of PSII (Yamanaka et al. 2012). The 48 BS configurations for the CaMn_4_O_5_(H_2_O)_4_ cluster have been constructed to elucidate the most stable ground MV configuration (Yamanaka et al. 2012) under the assumption of the HR XRD structure (Umena et al. 2011). The BS solutions were constructed by using our own program systems for rapid conversions in Fig. S2. The relative energies of the ground and lower excited configurations revealed by our BS computations were depicted in Fig..

**Fig. 9:**
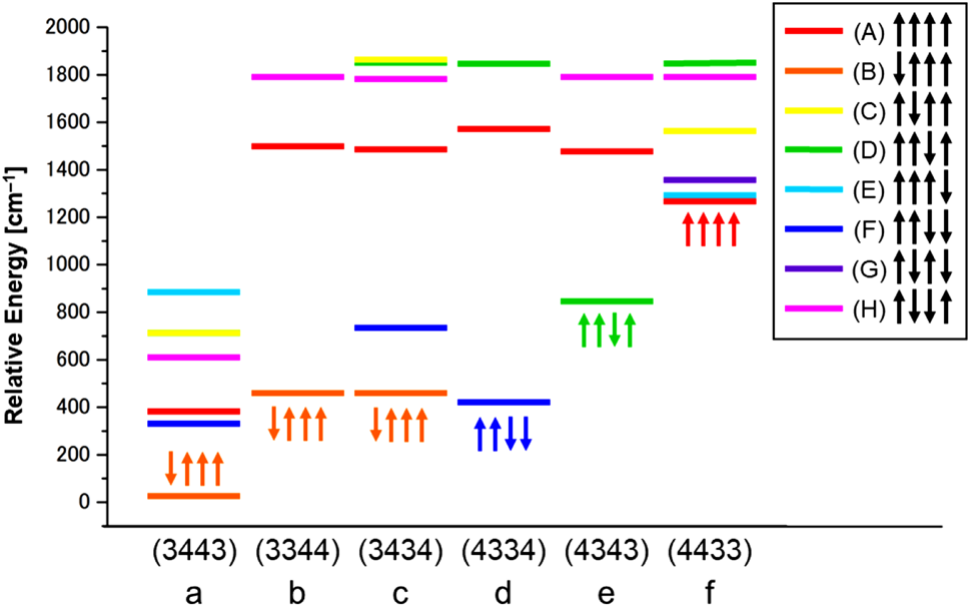
Energy levels (cm^−1^) of the total 48 (= 8 ×6) spin-charge configurations in the S1 state of the CaMn4O5 cluster by UB3LYP computations, providing the ground and lower-lying states (Yamanaka et al. 2012)

From Fig. 9, the Ca(II)Mn(III)_1_Mn(IV)_2_Mn(IV)_3_Mn(III)_4_ MV configuration was the most plausible for the CaMn_4_O_5_(H_2_O)_4_ cluster (**1**) in the S_1_ state. This MV configuration was abbreviated as the (3443) structure. The (3443) MV ground configuration in the S_1_ state was found to be consistent with the proposal based on the HR XRD structure (Umena et al. 2011). However, it was different from the (3344) MV structure proposed by EXAFS experiment by Berkeley group (Yano et al. 2005a) and early BS computation (Siegbahn 2006). Therefore, we must perform a number of full geometry optimizations by changing trial geometries of **1** for elucidation of the most plausible MV configuration in the S_1_ state of OEC of PSII (Isobe et al. 2012; Shoji et al. 2015a, b). Nowadays, the (3443) valence state in Fig. 9 is settled in the S_1_ state of the Kok cycle, supporting the early conclusion (Yamanaka et al. 2012).

### Refinements of the HR XRD structure by the full geometry optimizations

From our guiding principles in Fig. 2, spectroscopic experimental results are crucial and very important for examination of scope and reliability of HR XRD structure (Umena et al. 2011). Before the discovery of HR XRD structure, available EPR experimental results for **1** were elucidated to be the total ground singlet state (*S* = 0) with the low-lying excited triplet state (*S* = 1) in the S_1_ state (Yamauchi et al. 1997). On the other hand, the intermediate spin ground state (*S* = 3) was obtained by the HDFT computation (Yamanaka et al. 2012) under the assumption of the geometry revealed by the HR XRD structure in contradiction to the EPR results (Yamauchi et al. 1997) as shown in Fig. . This discrepancy clearly indicated the necessity of further refinements of the geometrical structure of the CaMn_4_O_5_(H_2_O)_4_ cluster with the (3443) valence state by the HR XRD. In fact, several papers (Galstyan et al. 2012; Askerka et al. 2017) against the HR XRD structure (Umena et al. 2011) pointed out that the X-ray damage (Yano et al. 2005b; Holton 2009) was the origin of the discrepancy.

According to the guiding principles in Fig. 2, possible reductions of high-valent Mn ions are classified into three categories on the theoretical grounds; (a) internal reduction, (b) semi-internal reduction, and (c) external reduction (Yamaguchi et al. 2014). The internal reduction means the reduction of high-valent Mn(IV) ions via the internal partial charge transfers (CT) from the oxygen dianions (O^2–^) to the Mn(IV) ions. The spin densities are induced on the oxygen dianion sites in the case (a) in conformity with our early BS studies on high-valent transition metal-oxo compounds (Yamaguchi et al. 1986). Indeed, negative spin densities (Kanda et al. 2011a, b; Yamanaka et al. 2012) were induced on the oxygen sites of **1** under the assumption of HR XRD structure, indicating that some of the Mn(IV)-O bonds were labile as compared with those of the inorganic Mn model complexes (Yamaguchi et al. 2014). Thus, one of the origins of the internal reduction was considered to be the experimental uncertainty (0.16 Å) in the HR XRD structure at 1.9 Å resolution. Therefore, to eliminate this error, we have performed full geometry optimizations of **1** starting from HR XRD under the assumption of the trial (3443) valence state revealed by the EXAFS experiments (Dau et al. 2008).

The partial elongation of some of the Mn–O bonds furthermore entailed the semi-internal reduction of the high-valent Mn(IV) ions via the charge transfers from anionic amino acid residues such as Asp61 anion to the Mn(IV) ions as revealed by large-scale QM(380 atoms)/MM calculations (Shoji et al. 2015a, b) under the assumption of the HR XRD structure. Additions of invisible hydrogen atoms by HR XRD (Umena et al. 2011) were necessary for construction of water molecules in hydrogen bonding networks around **1**. Thus, to eliminate the semi-internal reduction, we must perform the full geometry optimizations using the large-scale QM model under the QM/MM approximation. Full geometry optimizations by the QM/MM computations (Shoji et al. 2015a, b) were indeed effective for elimination of the semi-internal reduction (b) for refinement of the HR XRD structure of **1**. Judging from the QM/MM results, back electron transfer from quinone (Q_A_) site to the CaMn_4_O_5_(H_2_O)_4_ cluster was also regarded as a semi-internal reduction of **1**. The meta-stable S_2_ and S_3_ states are also going back to the stable S_1_ state via the semi-internal reduction. Thus, full geometry optimizations were feasible under the assumption that topological structure of **1** by HR XRD is reliable enough for theoretical refinements.

The reduction of the high-valent Mn ions by electrons generated by decompositions of water molecules and other amino acid residues in OEC of PSII by strong X-rays was classified as the external reduction (c) in our terminology (Yamaguchi et al. 2014). Yano et al. have elucidated that the external reductions are highly dependent on the experimental conditions such as dose and flux of the X-ray radiation, together with temperature, pH, etc. in the XRD experiments (Yano et al. 2005b). Therefore, the (3443) valence configuration in the S_1_ state was supposed to be converted into several lower oxidation states in the case (c). Galstyan et al. indeed performed theoretical computations for elucidation of degree of the external reduction that was highly dependent on the valence states and protonation states of oxygen dianions involved in **1** (Galstyan et al. 2012).

Unfortunately, hydrogen atoms were invisible by HR XRD, providing no information for the protonation states, which are closely related to the valence states of the Mn ions of **1**. Therefore, to avoid the external reduction, we have performed the constructive refinement of the HR XRD structure (Umena et al. 2011) by the QM (Isobe et al. 2012) and QM/MM (Shoji et al. 2015a, b) by fixing the high-oxidation valence state of Mn(III)_2_Mn(IV)_2_ in the S_1_ state under the assumption of the parent structure of CaMn_4_O_5_(H_2_O)_4_ of **1**.

The structural parameters for the refined “distorted chair structure” of **1** by the theoretical computations were indeed found to be compatible with those of EXAFS experiments (Yachandra et al. 1996). The QM (Isobe et al. 2014) computations also provided the *S* = 0 ground state observed by EPR (Yamauchi et al. 1997) after the structure refinements. This finding indicated the utility and applicability of the QM and large-scale QM/MM computations for refinements of the HR XRD structure (Umena et al. 2011). Interplay between theory and experiment was effective for elimination of a possible uncertainty of HR XRD at 1.9 Å resolution. Moreover, theoretical computations are in turn applicable to full geometry optimizations of possible intermediates obtained by proton shifts from the parent structure of CaMn_4_O_5_(H_2_O)_4_ of **1** in the S_i_ (*i* = 0 ~ 3) states (see Table 1). Later, the refined structure of **1** under the assumption of the (3443) valence state was indeed found to be compatible with the damage-free structure (A-monomer) by the extra low-dose (0.03 MGy) XRD experiment (Tanaka et al. 2017).

**Table 1:** The Mn_a_-Mn_b_ distances of the CaMn_4_O_5_ cluster in the S_i_ (*i* = 0 ~ 3) states of OEC of PSII by large-scale QM/MM and estimation (in parentheses) procedure

Type^a)^	Structures^b)^	Mn_a_-Mn_b_^c)^	Mn_a_-Mn_b_	Mn_a_-O_(5)_^d)^	SSB^e)^	Topology^f)^
		QM/MM	Estimation	QM/MM		
I	3ARC(XRD)^g)^	2.97	2.96	2.50	0.05	C
	3WU2(XRD)^g)^	2.93	2.91	2.40	0.15	C_R_
	XFEL SFX^g)^	2.87	2.86	2.30	0.20	C_R_, {2, 1, 0}
II	S_0ac_(QM/MM)	2.71(2.71)	2.72 (2.72)	1.88 (1.87)	0.59 (0.61)	R, {2, 1, 0}
	S_0bc_(QM/MM)	2.92(2.91)	2.84 (2.81)	2.26 (2.20)	0.25 (0.33)	C_R_, {1, 2, 0}
	S_0bc_(Estimation)^h)^	–	3.10 (3.14)	2.78 (2.86)	− 0.25 (− 0.33)	C_L_, {1, 2, 0}
	S_0bb_(QM/MM)	3.00(2.96)	3.11 (3.01)	2.79 (2.60)	− 0.27 (− 0.13)	C_L_, {1, 1, 1}
	S_0bb_(Estimation)^h)^	–	2.84 (2.91)	2.26 (2.40)	0.27 (0.13)	C_R_, {1, 2, 0}
	S_0cc_(QM/MM)	3.10	3.08	2.73	0.01	C, {1, 1, 1}
III	S_1ac_(QM/MM)	2.72 (2.71)	2.74 (2.71)	1.93 (1.82)	0.54 (0.71)	R, {2, 1, 0}
	S_1ab_(QM/MM)	2.78	2.77	2.06	0.37	R, {1, 2, 0}
	S_1ab_(QM/MM)	2.7	2.8	1.9	> 0.5	R, {1, 2, 0}
	S_1bb_(QM/MM)	3.08	3.10	2.77	− 0.19	C_L_, {1, 1, 1}
	S_1bb_(Estimation)^h)^	–	2.89	2.34	0.19	C_R_, {2, 1, 0}
	S_1bb_(QM)	2.91	2.88	2.34	0.20	C_R_, {2, 1, 0}
	S_1bb_(QM/MM)	3.03	3.04	2.64	− 0.11	C_L_, {1, 1, 1}
	S_1bc_(QM/MM)	3.05	3.08	2.74	− 0.14	C_L_, {1, 1, 1}
	S_1bc_(Estimation)^h)^	–	2.91	2.39	0.14	C_R_, {2, 1, 0}
IV	S_2ac_(QM/MM)	2.73	2.70	1.80	0.69	R, {3, 0, 0}
	S_2ab_(QM/MM)	2.75	2.72	1.82	0.65	R, {3, 0, 0}
	S_2ac_’(QM/MM)	3.12	3.04	3.13	− 0.64	L, {2, 0, 1}
	S_2ab_’(QM/MM)	3.14	3.04	3.14	− 0.65	L, {1, 1, 1}
V	S_3ab_(QM/MM)^i)^	2.78	2.73	1.92	0.75	R, {0, 3, 0}
	S_3ab_’(QM/MM)^i)^	3.20	3.12	3.46	− 0.81	L, {1, 1, 1}
	S_3ab_(QM/MM)^j)^	3.14	3.05	3.18	− 0.67	L, {1, 1, 1}
	S_3ab_(Estimation)^j)^	–	2.72	1.86	0.67	R, {0, 3, 0}

^a)^Type: I experiments, II S_0_ state, III S_1_ state, IV S_2_ state, and V S_3_ state; ^b)^Structures S_iXY_ (*i* = 0 ~ 3) in Fig. 10; ^c)^The optimized Mn_a(4)_-Mn_b(3)_ distance by QM/MM (Shoji et al. 2015a, b) and that by QM (Isobe et al. 2012) is given in parentheses; ^d)^The estimated Mn_a(4)_-Mn_b(3)_ distance using the optimized Mn_a(4)_-O_(5)_ by QM/MM (Shoji et al. 2015a, b) and that by QM (Isobe et al. 2012) is given in parentheses; ^e)^Structural symmetry breaking (SSB) parameter; ^f)^The numbers of the shortest (2.7 Å), short (2.8 Å), and longer (3.0 Å) Mn–Mn distances are given by {d, e, f}; ^g)^The Mn_a(4)_-Mn_b(3)_ distance was estimated by using the observed Mn_a(4)_-O_(5)_ distance; ^h)^No QM/MM(QM) results and estimated values by using EXAFS and others; ^i)^W (= OH^−^) was inserted in the S_3_ state (see Fig. 2). ^j)^W (= OH^–^) was not inserted in the S_3_ state

### Jahn–Teller distortion by the Mn(III) ion of the CaMn_4_O_x_ cluster

**Fig. 10:**
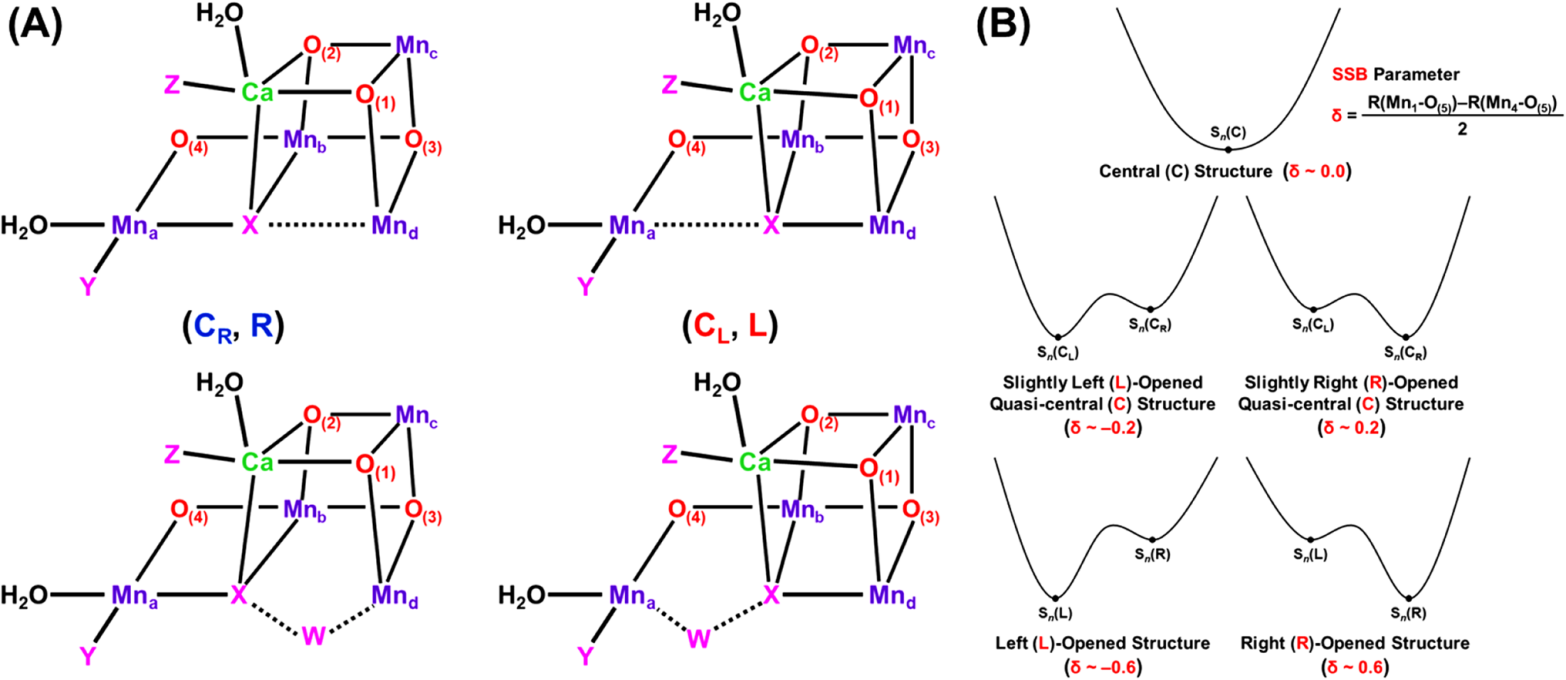
**A** The O_(5)_ (= X) site in the symmetrical center (C) structure is unstable, converting into the quasi-central right (R) and left (L)-elongated structures (C_R_, C_L_) and R- and L-opened structures. Water (W) is inserted for the R- and L-opened structures, providing (R-W, L-W) structures. **B** Potential curves for symmetry breaking structures, where *δ* denotes the SSB parameter

According to our principle in Fig. 2, the orbital degree of freedom, namely JT effect of the Mn(III) ion is important for geometry changes of **1** with the (3443) MV configuration in the S_1_ state. Full geometry optimizations of the S_1_ cluster with (3443) by QM (Isobe et al. 2012) and QM/MM (Shoji et al. 2015a, b) indeed revealed the right (R)-opened (open cubane) and left (L)-opened structures because of the JT effect of the Mn(III) ion as illustrated in Fig. 10A. The Mn_a(4)_-O_(5)_ and Mn_d(1)_-O_(5)_ distances were variable via the JT effect because of the labile nature of the Mn_a(4)_-O_(5)_-Mn_d(1)_ bond (Kanda et al. 2011b). Therefore, the structural symmetry breaking (SSB) parameter was defined by using their distances; SSB = [R(Mn_d(1)_-O_(5)_) – R(Mn_a(4)_-O_(5)_)]/2 to elucidate its magnitude. Thus, different geometrical structures via the JT effects are feasible even for **1** with the same spin and charge structures in other S_i_ states.

As mentioned above, hydrogen atoms were invisible by the HR XRD experiment, providing no experimental information for discrimination between O_(5)_ = O^2−^ and O_(5)_ = OH^−^. Therefore, full geometry optimization was performed under the assumption that the O_(5)_ site was protonated in the S_1_ state, providing slightly right-elongated quasi-central (C_R_) structure with small SSB parameter SSB = (2.7–2.3)/2 = 0.2 as illustrated in Fig. 10. The C_R_ structure was consistent with the refined HR XRD structure (Isobe et al. 2014) and SFX XFEL structure (Suga et al. 2015). On the other hand, the optimized Mn_a(4)_-O_(5)_ and Mn_d(1)_-O_(5)_ distances (Isobe et al. 2012) were about 1.9 and 3.0 Å, respectively, under the assumption that the O_(5)_ site was oxygen dianion in the S_1_ state. The SSB parameter was calculated to be 0.55 Å, indicating the right-opened (R) structure. The R-structure was later found to be consistent with the S_1_ structure by the refined EXAFS (Yano and Yachandra 2014).

The QM (Isobe et al. 2012) and QM/MM (Shoji et al. 2015a, b, c) computations were performed for possible intermediates in the S_i_ states (*i* = 0, 1, 2, 3) of the Kok cycle under the high-oxidation valence state. The R-structure was obtained for the optimized S_2_ structure with the (3444) valence state by the d(1)c(2)b(3)a(4) notation in accord with the JT effect of the Mn(III)_1(d)_ ion as shown in Fig. 10. On the other hand, the left (L)-opened structure was obtained for the optimized S_2_ structure with the (4443) valence state in accord with the Jahn–Teller effect of the Mn(III)_4(a)_ ion, providing the negative SSB parameter (− 0.55). Pantazis et al. have also elucidated both R- and L-structure of the 3H structure model: CaMn_4_O_4_(OH)(H_2_O)_4_ in the S_2_ state (Pantazis et al. 2012). Guidoni group also obtained the same conclusion by QM/MM computations (Bovi et al. 2013; Narzi et al. 2014), proposing the water insertion at the L-opened (closed-cubane) conformation (see also Capone et al. 2016). Thus, the JT effects of the Mn(III) ion are crucial for understanding and explanation of structural distortions of **1** in the S_2_ state of the Kok cycle.

Because of the SSB structures in the S_2_ state, water insertion into the reaction site of **1** was feasible via the proton-coupled electron transfer (PCET) reaction in the S_2_ to S_3_ transition, providing the early computational result of hydroxy anion insertion in the S_3_ state as shown in Fig. 10A (Isobe et al. 2012, 2014; Shoji et al. 2015c). Past decade, many experimental and theoretical investigations have been performed to elucidate mechanisms of a water insertion in the S_2_–S_3_ transition. To avoid elongation of the review, we have summarized essential results of these important investigations (Umena et al. 2011; Retegan et al. 2016; Askerka et al. 2015; Wang et al. 2017; Capone et al. 2015, 2016; Siegbahn 2018; Okamoto et al. 2021; Hussein et al. 2021) in the supporting section (SI Part IV). Available spectroscopic results (Renger 2012) have suggested multiple S_3_ intermediates, and EXAFS results (Yachandra et al. 1996) have indicated multiple valence configurations Mn(IV)_4_ and Mn(III)Mn(IV)_3_-O· in the S_3_ state of the Kok cycle. Therefore, these experimental and theoretical results urged us to perform successive detailed examinations of all possible S_3_ structures of **1** after water insertion by the QM methods (Isobe et al. 2016).

Surprisingly, damage-free XFEL experiments (Young et al. 2016) have however reported no structural information of the water insertion in the S_3_ state. Therefore, we have further performed full geometry optimizations of possible S_3_ intermediates before and after water insertion by the large-scale QM/MM method (Shoji et al. 2017), indicating that the Mn–Mn distances observed by the SFX XFEL (Young et al. 2016) are compatible with those of the R-opened hydroxide-inserted S_3_ structure by QM/MM. On the other hand, the XFEL results by Okayama group (Suga et al. 2017) have indicated the water insertion (H_2_O_(6)_) in the S_3_ state, reporting the short O_(5)_–O_(6)_ distance responsible for the O–O bond formation. Indeed, the reported O_(5)_–O_(6)_ distance (Suga et al. 2017) was consistent with that of the peroxide structure by QM (Isobe et al. 2016).

Serious discrepancy between the XFEL results (Young et al. 2016; Suga et al. 2017) has urged us to examine the four degrees of freedom for **1** in Fig. 2, namely guiding principle of our theoretical investigation (Yamaguchi et al. 2018). The mixed-valence configuration of the optimized geometry of peroxide intermediate in the S_3_ state by large-scale QM/MM (Shoji et al. 2017) was (3443) in contradiction to the (4444) valence state by EXAFS (Yano and Yachandra 2014), indicating discrepancy from the charge degree of freedom. On the other hand, no water insertion entailed the L-opened structure with *S* = 6 in the S_3_ state in contradiction to the EPR result (Cox et al. 2014), namely spin degree of freedom. Therefore, further theoretical investigations were indispensable for the S_3_ state at that time (see later).

### Estimation formula for Mn–O bond lengths of the CaMn_4_O_x_ cluster in the Kok cycle

The low-dose XRD structures (Tanaka et al. 2017) have provided important information of geometrical parameters of **1** in the S_1_ state. Judging from the low-dose results, the uncertainty of the Mn–Mn distances of **1** by HR XRD structures was found to be relatively small because of several reasons. Similar situations are conceivable even for the XFEL results in the S_2_ and S_3_ states. On the other hand, Mn–O distances of **1** might involve non-negligible experimental uncertainty as compared with the Mn–Mn distances. This might be an origin of the uncertainty of the O_(5)_-O_(6)_ distance. Therefore, practical theoretical model is desirable for elucidation of key Mn–O distances of **1**.

Our QM and QM/MM computational results of **1** have revealed that the Mn_a_-Mn_b_ distance is qualitatively correlated with Mn_a_-O_(5)_ distance of **1**. An estimation equation of the Mn_a_-O_(5)_ distance by the use of Mn_a_-Mn_b_ distance was derived as follows (Yamaguchi et al. 2014; Shoji et al. 2015a, 2019a),

4 $${\text{R}}\left( {{\text{Mn}}_{{\text{a}}({4})} - {\text{Mn}}_{{\text{b}}({3})} } \right) \, = { 2}.{8}0 \, +x/{2}n$$

where the parameter *x* is defined by

5 $${\text{R}}\left( {{\text{Mn}}_{{\text{a}}({4})} - {\text{O}}_{({5})} } \right) \, = { 2}.{18 } + x$$

6 $${\text{R}}\left( {{\text{Mn}}_{{\text{d}}({1})} - {\text{O}}_{({5})} } \right) \, = { 2}.{88 }-x.$$

The *n*-values were taken to be 1 for O_(5)_ = OH^−^ and 2 for O_(5)_ = O^2−^, respectively. The *x*-value was determined by using the calculated Mn_a_-Mn_b_ distances by our QM and QM/MM methods and by the observed distances. The structural symmetry breaking (SSB) parameter was also given by

7 $${\text{SSB }} = \, {{\left[ {{\text{R}}\left( {{\text{Mn}}_{\text{d}} - {\text{O}}_{({5})} } \right) \, -{\text{ R}}\left( {{\text{Mn}}_{\text{a}} - {\text{O}}_{({5})} } \right)} \right]} / 2} \, = \, 0.{35 }-x.$$

The *x*-value and R(Mn_a_-Mn_b_) distance were in turn estimated by using the R(Mn_a_-O_(5)_).

Table 1 summarizes the estimated R(Mn_a_-Mn_b_) and R(Mn_a_-O_(5)_) distances and SSB parameters that are used for elucidation of topological structures of **1** in the S_i_ (*i* = 0 ~ 3) states of OEC of PSII. From Table 1, characteristic features of the structures of **1** were elucidated in a qualitative manner under the estimation procedures. The Mn_a_-Mn_b_ distances of 3ARC and 3WU2 by HR XRD (Umena et al. 2011) were about 3.0 Å in contradiction to the EXAFS (Yachandra et al. 1996) and low-dose XRD (Tanaka et al. 2017) results, namely R(Mn_a_-Mn_b_) = 2.7 ~ 2.8 Å for the S_1_ structure. The O_(5)_ site was assumed to be O^2–^ (= a) or OH^–^ (= b) and W2 was assumed H_2_O (= c) and OH^–^ (= b) in the S_iXY_ structures (X = O_(5)_ and Y = W_2_). The Mn_a_-O_(5)_ distances were in the range 2.4 ~ 2.5 (Å) by HR XRD (Umena et al. 2011) in contradiction to 1.9 Å by EXAFS (Yachandra et al. 1996). Therefore, the refinements of the HR XRD structure by the full geometry optimization of the QM and QM/MM methods were crucial to eliminate errors arising from several origins. The calculated and estimated geometrical parameters for R, C_R_, and C_L_ structures in the S_1_ state are summarized in Table 1 (Shoji et al. 2015a, b).

The one-electron reduction and oxidation of the dark stable S_1_ state provide transient S_0_ and S_2_ states in the Kok cycle. The QM (Isobe et al. 2012) and QM/MM (Shoji et al. 2015b) computations have elucidated the labile nature of the Mn_a_-O_(5)_-Mn_d_ bond in the case of the S_0_ state, providing C (central), R, C_R_, and C_L_ structures, depending on the protonation states of the O_(5)_ and W2 sites (S_iXY_). The S_0bc_(QM/MM) structure was concluded to be the most plausible. On the other hand, S_2ac_(QM/MM) and S_2ab_(QM/MM) structures were consistent with the EXAFS structure (Yachandra et al. 1996) in the S_2_ state. Interestingly, two-left (L) opened (S_2_(L), S_2_(L)’) structures were obtained for the S_2_ state by our QM and QM/MM computations (Isobe et al. 2012). The Mn_a_-O_(5)_ distances were estimated by using the Mn_a_-Mn_b_ distances obtained by full geometry optimizations as shown in Table 1.

Theoretical computations were also extended to the S_3_ intermediates after water insertion in the S_2_ to S_3_ transition (Isobe et al. 2012; Shoji et al. 2015b, c). The structural symmetry breaking (SSB) for the R- and L-opened structures obtained by the insertion of W (= OH^−^) in Fig. 10B was also remarkable in the S_3_ state as shown in Table 1. Importantly, the O_(5)_-O_(6)_ distance estimated by using the observed Mn_a_-Mn_b_ distance (Suga et al. 2017) in eqs. (4–6) was elongated to be 1.9 Å, indicating no O–O bond formation in the S_3_ state (Yamaguchi et al. 2018). The uncertainty of the Mn_a(4)_-O_(5)_ and Mn_d(1)_-O_(5)_ distances was remarkable for **1** in the S_3_ state, suggesting the necessity of further refinement of the XFEL result (Suga et al. 2017). The refined results (Suga et al. 2019) indeed elucidated no OO bond formation, which is consistent with SFX XFEL (Kern et al. 2018).

Thus, the QM (Isobe et al. 2012) and QM(380 atoms)/MM computations ( Shoji et al. 2015a, b) starting from the HR XRD results (Umena et al. 2011) have provided geometrical structures of whole possible intermediates in the Kok cycle before the SFX XFEL experiments. The refined HR XRD structure (Isobe et al. 2012) was indeed compatible with the damage-free XFEL structure in the S_1_ state (Suga et al. 2015). Krewald et al. have also performed BS QM computations coupled with EPR results for whole possible intermediates under both high- and low-oxidation assumptions for the Kok cycle (Krewald et al. 2015). Thus, guiding principles for strongly correlation electron systems (SCES) in Fig. 2 are applicable to elucidation and explanation of the structure and bonding of **1** in the Kok cycle for water oxidation. Interplay between theory and experiment has provided important and reliable structural information for native water oxidation as summarized in a recent review article (Yamaguchi et al. 2022a).

### Theoretical investigations of EPR results to elucidate spin states

Spectroscopic experiments such as EPR (Boussac et al. 1990; Peloquin et al. 2000; Lubitz et al. 2008) provide important information for assignments of reasonable acceptable structures among theoretically possible structures of **1** as shown in Fig. 2. Historically, magnetic susceptibility measurements were available experimental procedures to examine structure and bonding of binuclear transition metal complexes in Figs. 3A-D (Sauer 1980), elucidating effective exchange coupling constants (*J*_ab_) between local spins (***S***_a_) of Mn ions. The spin Hamiltonian models given by *H* = –2*J*_ab_ ***S***_a_·***S***_b_ (Yamanaka et al. 2012) were employed for analysis of the observed temperature dependent paramagnetism even for transition metal complexes with the total singlet ground state (Yamaguchi 1990b). The observed paramagnetism was qualitatively compatible with the BS model (Yamaguchi et al. 1986). Therefore, we derived computational schemes of *J*_ab_ parameters by using total energies (.^X^*E*) of BS solutions for the low-spin (LS) and high-spin (HS) configurations as follows (Yamaguchi et al. 1986)

8 $$J_{{\text{ab}}} = [^{{\text{LS}}} E-^{{\text{HS}}} E\left] { \, / \, } \right[^{{\text{HS}}} < S^{2} > -^{{\text{LS}}} < S^{2} > ].$$

**Fig. 11:**
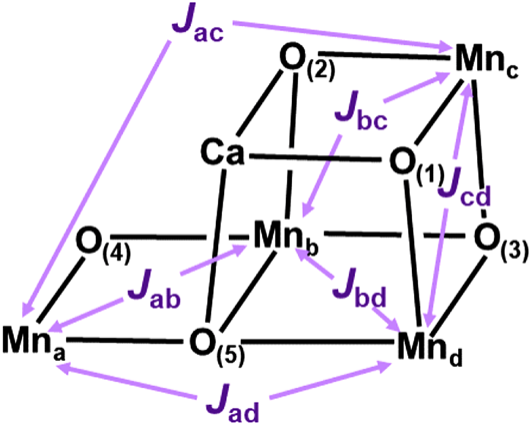
Effective exchange integrals between Mn ions of the CaMn_4_O_5_ cluster in the Heisenberg spin Hamiltonian model (notations are a = 4, b = 3, c = 2, and d = 1 in the text)

The ^X^ < ***S***^2^ > denotes total spin eigenvalue for the spin state X. The *J*_ab_ parameters calculated for the M-(μ-O)-M and M(μ-O)_2_M (Yamaguchi et al. 1988a) were compatible with available experimental results (Peloquin et al. 2000; Lubitz et al. 2008). The *J*_ab_ parameters for the Mn–O-Mn with shorter Mn–Mn distances than 3.0 Å were negative in sign, indicating the antiferromagnetic (AF) exchange interaction (Yamaguchi et al. 1988a). On the other hand, spin crossover from AF to ferromagnetic (FR) ground state for longer Mn–Mn distances than 3.0 Å, indicating similar behavior of the *J*_bd_ parameter of **1** in Fig. 11 (Yamanaka et al. 2012).

The BS computational scheme in Eq. (8) has been extended to elucidate *J*_pq_ parameters for multi-nuclear systems (Shoji et al. 2006) such as the CaMn_4_O_x_ clusters in Fig. 11. Six *J*_pq_ values in Fig. 11 can be determined by using total energies of the eight BS solutions in Fig. 7 (Kanda et al. 2011a). Three different methods were used for determinations of *J*_pq_ values (Isobe et al 2014); (a) vertical approximation where the geometry of the highest spin (HS) configuration was assumed for other seven configurations, (b) adiabatic approximation where full geometry optimizations of eight different spin configurations were performed to obtain total energies, and (c) zero-point energy (ZPE) corrections for eight different solutions were added for refinements of adiabatic total energies.

**Fig. 12:**
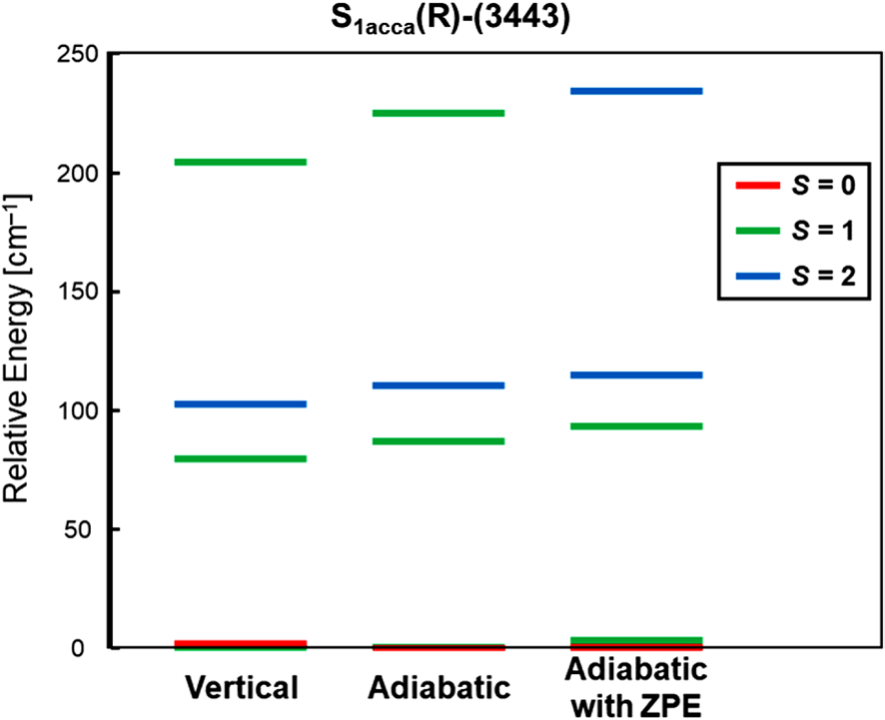
Energy levels of the S_1_ state of PSII by the exact diagonalization of the spin Hamiltonian consisted of *J*_ab_ values obtained by three computational method (Isobe et al. 2014)

As an example, the energy levels of the eight spin configurations for the S_1_ state were depicted under the assumption of O_(5)_ = O^2–^ (Isobe et al. 2014). All three methods indicated that the low-spin configuration with the up-down-down-up (↑↓↓↑) spin alignment was the ground state, indicating the triplet state (*S* = 1). However, the EPR experiments (Yamauchi et al. 1997) revealed the ground singlet state instead of the thermally excited triplet state in contradiction to the BS computational results (Isobe et al 2012). The exact diagonalization of the Heisenberg spin Hamiltonian was necessary to obtain the full energy levels of the ground and lower excited states detected by EPR at a low temperature. Figure 12 shows the energy levels in the S_1_ state of the R-structure, indicating that the ground singlet state with low-energy triplet state was obtained under the adiabatic + ZPE correction (Isobe et al. 2014) in accord with the EPR result (Yamauchi et al. 1997). Thus, very careful examinations were necessary for the EPR results for **1** in OEC of PSII.

A number of EPR experimental results have been published for the S_2_ state after early experiments (Sauer 1980; Dismukes and Siderer 1981). The full geometry optimization of the R-opened structure with the (3444) valence state provided the up-down-down-up (↑↓↓↑) spin alignment, indicating the doublet (*S* = 1/2) ground state (Isobe et al. 2012; Pantazis et al. 2012; Narzi et al. 2014) in agreement of available EPR results (Peloquin et al. 2000; Lubitz et al. 2008; Cox et al. 2011). On the other hand, full geometry optimizations of the L-opened structure elucidated two different structures with the same up-up-up-down (↑↑↑↓) spin structure (Yamaguchi et al. 2016), indicating the ground septet state (*S* = 5/2) states with different g-values in accord with the EPR experiments (Boussac et al. 2018; Boussac 2019). The SSB parameters were small and large for these L-opened structures; the latter structure with the notation’ was shown in Table 1. Interestingly, the R-opened proton-shifted isomer S_2abcb_(R) was also calculated to be the ground septet state (*S* = 5/2) (Corry and O'Malley 2019; Miyagawa et al. 2022a).

**Fig. 13:**
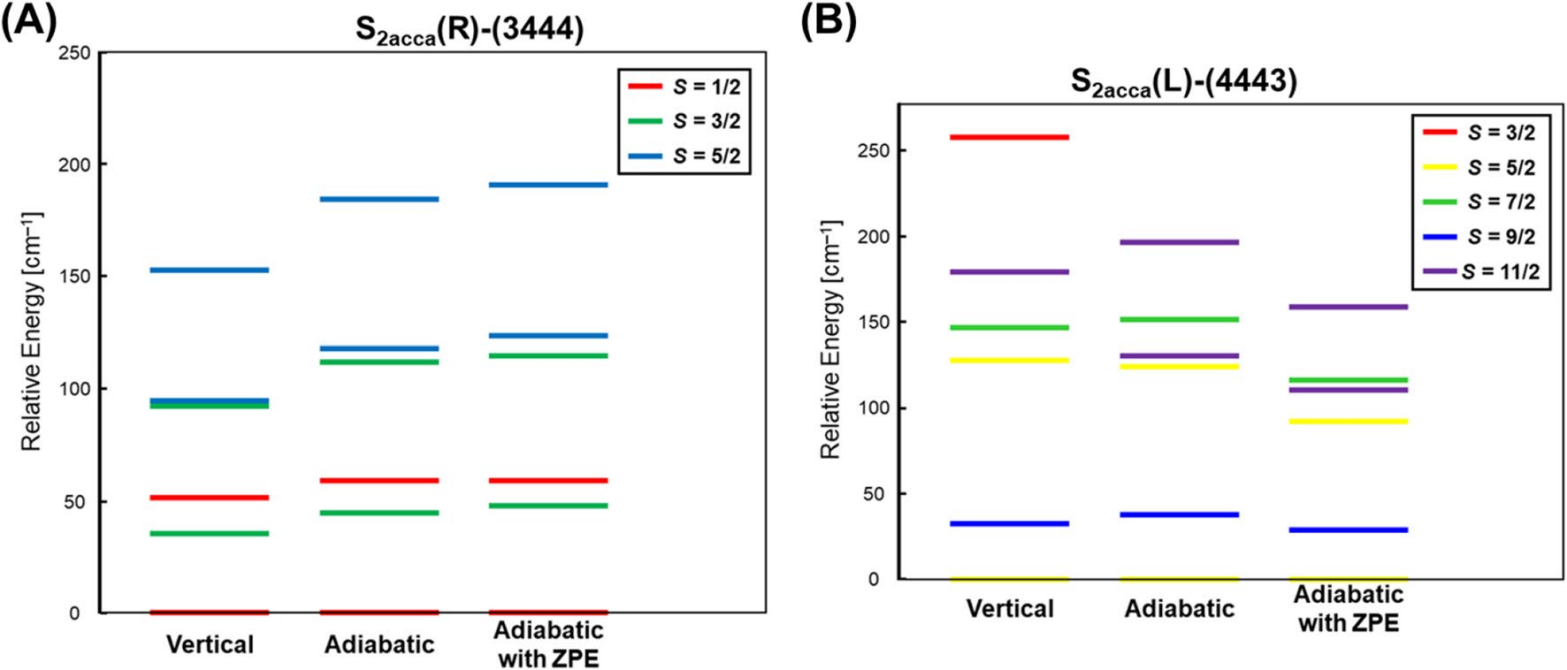
Energy levels for the right (R)- and left (L)-opened structures in the S_2_ state by the exact diagonalization of the spin Hamiltonian model consisted of *J*_ab_ values obtained by three different computational methods (Isobe et al. 2014; Yamaguchi et al. 2016)

Figure 13 illustrates the energy levels of the S_2acca_(R)-(3444) and S_2acca_(L)-(4443) structures by the exact diagonalization of the spin Hamiltonian matrices constructed with *J*_pq_ parameters of the three methods. A number of computations of *J*_pq_ parameters (Miyagawa et al. 2021) for different proton-shifted S_2_ isomers from the parent S_2_ CaMn_4_O_5_(H_2_O)_4_ cluster in Table 1 were also necessary for whole understanding of the EPR experiments (Boussac et al. 2018; Boussac 2019; Miyagawa et al. 2022a). The low-spin (*S* = 1/2, *g* = 2.0) and intermediate spin (*S* = 5/2, 7/2; *g* = 4.1, *g* > 4.1, *g* = 5) structures were obtained, depending on the R- and L-open structural topologies of **1** and proton shifts from H_2_O at W1 or W2 to the O_(4)_ or O_(5)_ site in the S_2_ state (Yamaguchi et al. 2016; Miyagawa et al. 2021). Thus, available EPR results on the proton shifts (freedom of dynamical nuclear motion) have urged us to examine possible important roles of hydrogen bonding networks around **1** in OEC of PSII. EPR results (Cox et al. 2014; Boussac et al. 2018; Chrysina et al. 2019; Boussac 2019) for the S_3_ state were also complex. Extensive BS computations of *J*_pq_ parameters followed by the exact diagonalization of the spin Hamiltonian (Shoji et al. 2006) were crucial to explain the EPR results of **1** in the S_3_ state of Kok cycle for water oxidation (Isobe et al. 2019; Miyagawa et al. 2022b). Therefore, these were abbreviated in this review.

The EPR spectroscopies have been successfully used to elucidate the populations (missing factors) of the S_i_ configurations in each flash (first to sixteen) in the Kok cycle (Han et al. 2022), showing subtle temperature dependence. The populations of the S_3_ configuration in the second flash were 62 (38) and 79 (21) % at – 10 °C and 10 °C, where the missing factors for S_2_–S_3_ transition were given in parentheses. Other missing factors also decrease with the temperature except for the S_1_–S_2_ transition. The missing factors (Han et al. 2022) have been used for analysis of the weights of the S_i_ configurations of SFX XFEL results in the first to third flashes (Kern et al. 2018).

## Biomolecular system structure of oxygen evolving complex of photosystem II

### Large-scale QM/MM approach to oxygen evolving complex of photosystem II

**Fig. 14:**
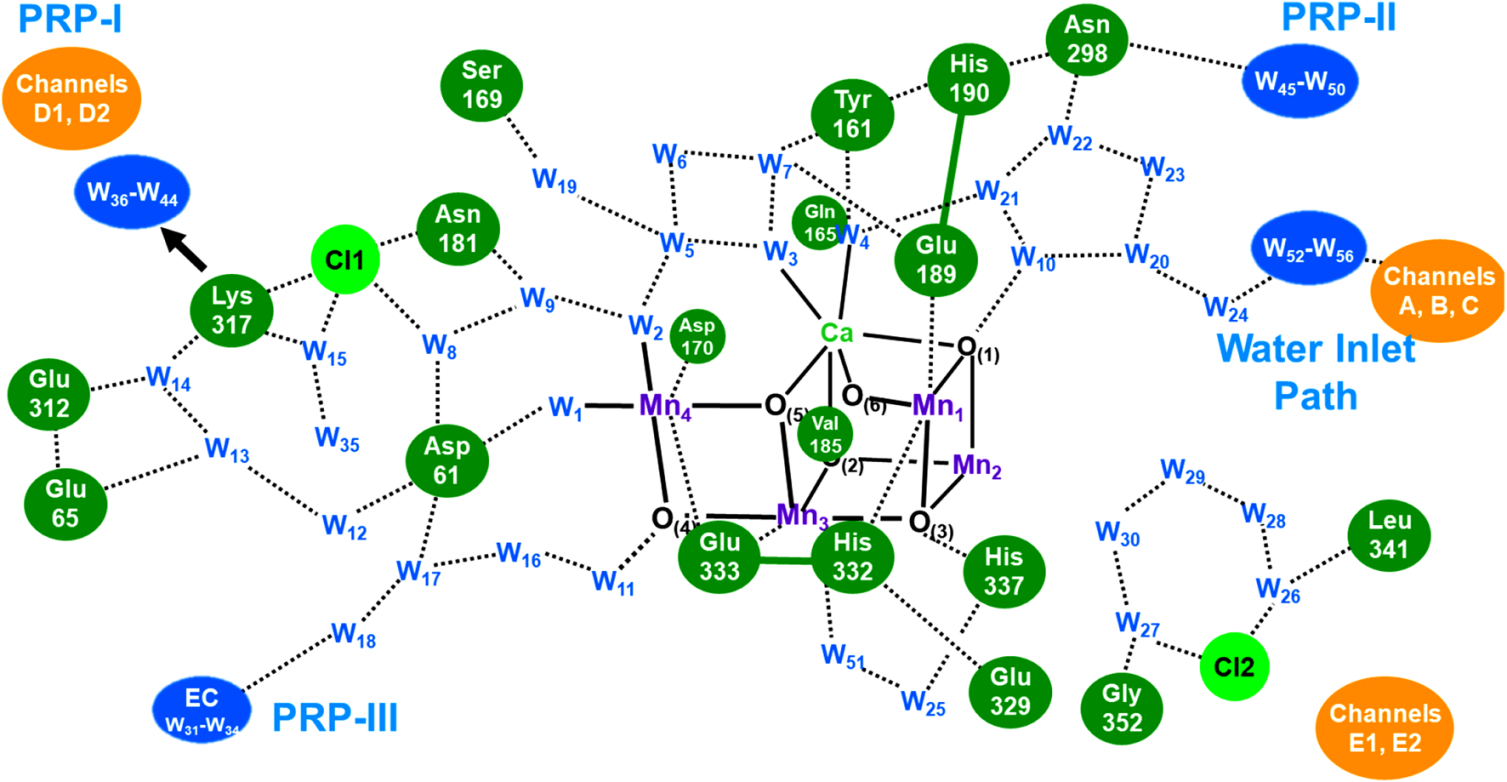
Hydrogen bonding networks around the CaMn_4_O_5_ cluster (**1**) which are linked with the water inlet channels A, B, and C, proton release pathways (PRP) I, II, and III revealed by HR XRD (Umena et al. 2011). The water inlet channels A, B, and C is often referred to as the O_(1)_ channel. PRP I is referred to as channels D1 and D2, which are often denoted as Cl-I and Cl-II channels, respectively. PRP II is referred to as Tyr161 (Y_z_)-channel. PRP III is often referred to as the O_(4)_ channel (see Table 2)

In this section, the biomolecular system structures of OEC of PSII are examined in relation to water inlet, proton shift and proton release pathways for water oxidation in Eq. (1). Our theoretical view of OEC of PSII is that the CaMn_4_O_x_ cluster (**1**) with the strong correlation electron system (SCES) is embedded in peripheral membrane proteins (PsbO, PsbU, and PsbV) and lipids (Isobe et al. 2012) as illustrated in Fig. 2. The HR XRD structure (Umena et al. 2011) disclosed a secret of the biomolecular system structure of which is consisted of the distorted chair-like CaMn_4_O_5_ cluster, amino acid residues, hydrogen bonding networks of waters, Cl1, Cl2, etc. as illustrated in the Fig. 14. The HR XRD structure provided a structural foundation for constructions of our several biomolecular system models of OEC of PSII although it might be suffered from the X-ray damage (Yano et al. 2005b; Galstyan et al. 2012). Therefore, refinements of the biomolecular system structures around **1** were crucial.

Large-scale QM (380 atoms)/MM computations (Shoji et al. 2015a, b) were performed for confirmation and refinement of the hydrogen bonding networks around **1** revealed by the HR XRD structure (Umena et al. 2011). As mentioned above, additions of hydrogen atoms were necessary to construct water molecules and amino acid residues to complete hydrogen bonding networks around **1** because they were invisible by HR XRD at the 1.9 Å resolution. The hydrogen bonding distances and angles optimized by the QM(380 atoms)/MM calculations (Shoji et al. 2015a, b) are partly summarized in Table S2, indicating no significant X-ray damages of the hydrogen bonding networks around **1**. The refined distances between heavy atoms are later found to be consistent with the corresponding values observed by low-dose XRD (Tanaka et al. 2017). Key water molecules assigned as W1 ~ W27 in Fig. 14 have been included in the QM(380 atoms)/MM model and other water molecules (W28 ~ W56) are modeled by the MM model (Shoji et al. 2015a, b) (see supporting Table S2).

Two substrate water molecules are necessary for oxygen evolution in Eq. (1). The catalytic cluster (**1**) for water oxidation is constructed of the Mn4 and Ca sites coordinated with water molecules, which are linked with the hydrogen bonding networks (Saito et al. 2012). The refined hydrogen bonding structures were useful for understanding of water exchange experiments using H_2_^18^O/H_2_^16^O isotopes in combination with mass spectroscopic detection of the isotopologues of O_2_, elucidating slow (W_s_) and fast (W_f_) molecules among these key waters in each S_i_ state of OEC of PSII (Nilsson et al. 2014; de Lichtenberg et al. 2021). The water exchange experiments have elucidated that the central oxo bridge O_(5)_ was assigned as W_s_, while terminal water ligands W2 and W3 are regarded as W_f_ (Nilsson et al. 2014; de Lichtenberg et al. 2021). The substrate-water exchange has been dramatically slowed in the early transient S_4_ [S_3_ + Tyr-O radical] state, indicating strong hydrogen bonding interaction with the Ca(II) ion (Yamaguchi et al. 2019).

The HR XRD structure (Umena et al. 2011) has disclosed the water inlet pathway (WIP) connected with channels A, B, and C as shown in Fig. 14. In fact, glycerol molecules, as one of the crystallized materials for PSII, were observed in these channels, suggesting the large channel for water inlet in OEC of PSII. The damage-free low-dose XRD results (Tanaka et al. 2017) also elucidated the inclusion of glycerol molecules in WIP. A large-scale QM(380 atoms)/MM computations (Shoji et al. 2015a, b) have confirmed these channels which are often referred to as the O_(1)_ channel because of the hydrogen bonding between O_(1)_ and W10. The water inlet pathway (WIP = O_(1)_) provides a new water molecule at the W3 position through WIP-W24-W20-W10-W21-W4-W3 network. The hydrogen- bonding network (water wire); W3-W5-W2-W9-W8-Asp61-W12-W13-W14-PRP I has been effective for proton release as shown in Figs. 14. Interplay between QM/MM (Shoji et al. 2015a, b) and HR XRD (Umena et al. 2011) has elucidated the details of PRP I.

From Fig. 14, water molecules forming the hydrogen bonding networks connecting with W1 ~ W4 are named as W5 ~ W27 after confirmations and refinements of the hydrogen bond distances and angles by the QM (380 atoms)/MM computations as shown in Tables S2. The optimized QM/MM structure at the hydrogen atomic level resolution have eliminated the semi-internal reduction of HR XFD (Umena et al 2011), elucidating the biomolecular system structure of OEC of PSII. W19 is linked with W5 and Ser169 connected with PRP III. Thus, the water inlet pathway (WIP), PRP I, PRP II, and PRP III in our notation (Shoji et al. 2015a, b) are linked with the CaMn_4_O_5_ core complex (**1**) as shown in Fig. 14. They are often referred to as O_(1)_, O_(4)_, Tyr161, and Cl1-I(II) channels, respectively (Hussein et al. 2021).

SFX XFEL results (Hussein et al. 2021) have shown that the average B-factors for the waters up to approximately 15 Å from **1** in the O_(1)_, O_(4)_, and Cl1-I channels are about 38, 31, and 27 Å^2^, respectively, indicating the greater mobility of the O_(1)_ channel than that of others. These results by SFX XFEL are compatible with the WIP (O_(1)_ channel) revealed by the interplay between QM/MM and HR XRD (Shoji et al. 2015b). Recent SFX XFEL experiments (Kern et al. 2018; Suga et al. 2019; Hussein et al. 2021) have elucidated the dynamical mechanism of a water insertion (O_X_ or O_(6)_) in the S_2_ to S_3_ transition. Table 2 summarized several notations of important pathways (channels) in OEC of PSII.

**Table 2:** Notations of channel structures

QM/MM^a)^	SFX-XFEL^b)^	R(X–Y)^c)^	R(X–Y)^d)^
WIP	O(1)-channel	A, B, C	Yz-O1 path
PRP I_a_, I_b_	Cl1-channel	D1, D2	Cl-path
PRP II	Y_Z_-channel	PsbV	Yz-N298
PRP III	O(4)-channel	PsbU	O4 path

^a)^Shoji et al. (2015b), ^b)^Hussein et al. (2021), ^c)^Umena et al. (2011), and ^d)^Nagao et al. (2017)

Computational results by the large-scale QM/MM (Shoji et al. 2015a, b) have provided important information for understanding and explanation of several mutation experiments for amino acid residues of protein matrix of OEC of PSII by several pioneering groups (Debus 1992; Philbrick et al. 1991). They are also useful enough for explanation and confirmation of recent mutation experiments (Dilbeck et al. 2013; Nagao et al. 2017). Interestingly, Sugiura et al. have elucidated that proton release occurs in the S_1_ to S_2_ transition by the mutation of Val185 with threonine (Thr) (Sugiura et al. 2018). The S_3_ intermediate has been observed even for the mutant, however indicating dramatic slowdown for the S_3_-Tyr-O radical to the S_0_ transition in accord with variation of the hydrogen bonding network. Hydrogen bonding networks (see Table S2) consisted of key amino acid residues and water molecules (W1 ~ W56) investigated by the interplay between HR XRD and the QM/MM (Shoji et al. 2015b) are indeed crucial for understanding and explanation of the mutation results (see also supporting materials SI. Part V).

### Proton release pathway I (PRP I)

**Fig. 15:**
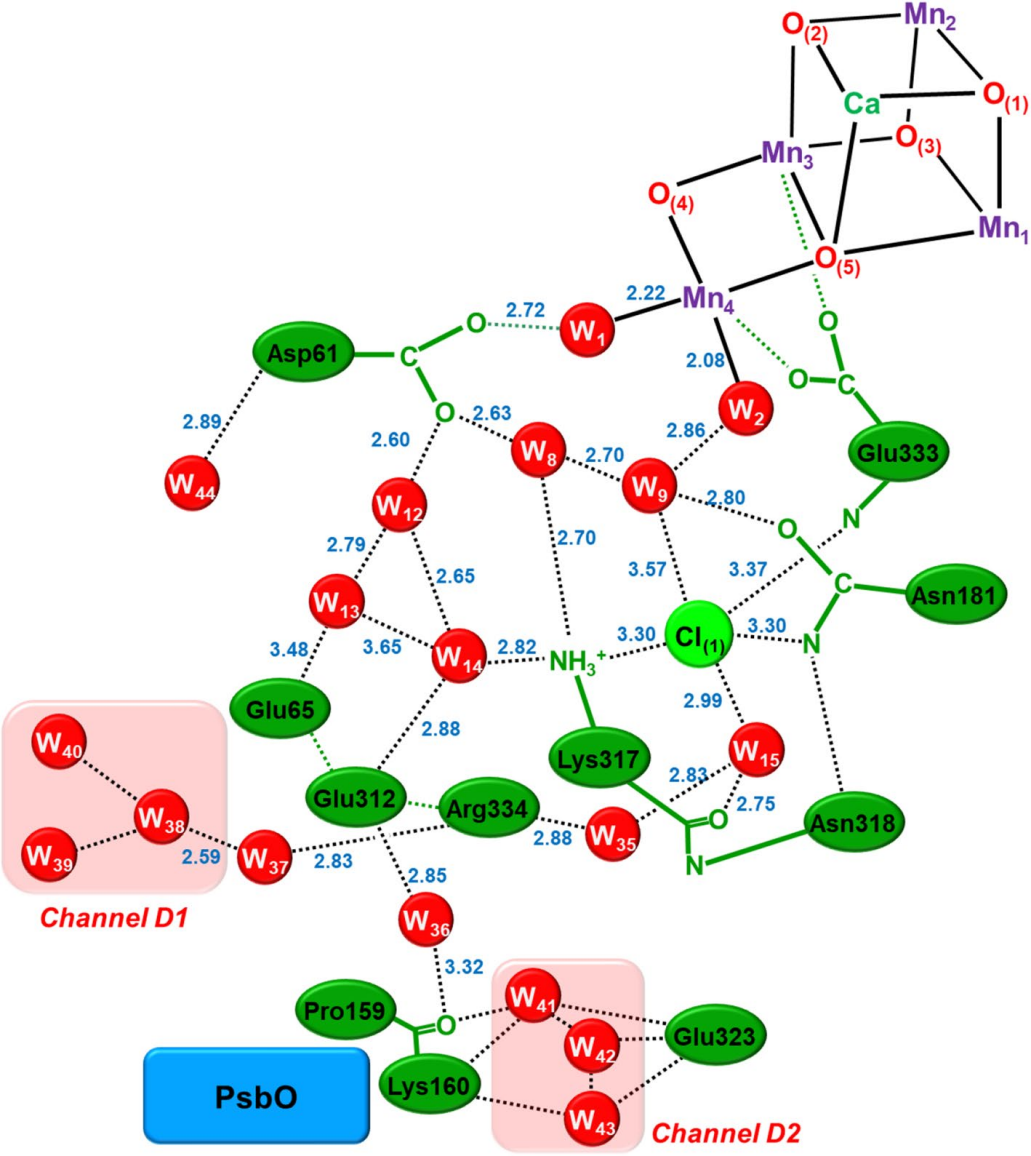
Hydrogen bonding networks for proton release pathway I (PRP I) proposed by HR XRD results (Umena et al. 2011). The large-scale QM(380 atoms)/MM computations (Shoji et al. 2015b) have been performed for refinements and confirmations of the HR XRD structure adding invisible hydrogen atoms. Water molecules by QM (380 atoms)/MM are denoted as W37 ~ W40 for the D1 and as W41 ~ W43 for D2 channels. D1 and D2 channels are often referred to as Cl-I_a_ and Cl-I_b_ channels, respectively

Proton release is a fundamental process for water oxidation in the Kok cycle in Fig. 14. Water chains observed by HR XRD (Umena et al. 2011) have indeed provided structural foundations for proton release pathways via the so-called Grotthuss mechanism. Asp61 has been regarded as a key amino acid residue in the proton release pathway I (PRP I) since it linked with W1 and W2−W9 (W22)-W8 (W21) molecules as shown in Fig. 15 (Shoji et al. 2015b; Greife et al. 2023; Bhowmick et al. 2023), where the numbers of water molecules in the S_3_ state by others (Hussein et al. 2021) are given in parentheses. Water molecules W12, W14 connected with Asp61, Glu65, and Glu312 have been included in the QM (380 atoms) part as shown in Fig. 15. On the other hand, the MM model has been used for modeling of the biomolecular systems consisted of water molecules W35 and W36, Arg344, D1 channel with W37 ~ W40, and D2 channel with W41 ~ W43 as shown in Fig. 15. W44 is linked with W12, W13, W14, which is connected with Asp61 and Glu65. Chloride anion (Cl1) is fixed with the hydrogen bonds with the amide backbone NH of Glu333, NH_2_ group of Asn181, NH_3_^+^ of Lys317, W9, and W15. Therefore, the coordination structure for Cl1 is different from Cl2, which is not connected with proton release channels E1 and E2 (Shoji et al. 2015b) in Fig. 14. On the other hand, Cl1 is connected with the proton release channels D1 and D2, which are often referred to as the Cl1-Ia and Cl1-Ib channels. Several different notations have been proposed for the same proton release pathways at the present stage as summarized in Table 2.

Recently, several experiments such as FTIR, EPR, etc. (Zaharieva and Dau 2019; Chrysina et al. 2019; Okamoto et al. 2021) have been performed extensively to elucidate proton release through PRP I (Shoji et al. 2015a, b) in the S_2_ to S_3_ transition. The FTIR experiments have revealed that proton release occurs before the OET to Tyr-radical in the S_2_ to S_3_ transition, providing crucial information to timing of proton-coupled electron transfer (PCET) process. Very recent XFEL SFX results (Hussein et al. 2021) have observed subtle movements of the W12 (W40)-W13 (W42)-W14 (W41) network (notations in parentheses are given by Hussein et al. (2021)), and Arg344, Lys317, and Glu312 in the S_2_ to S_3_ transition, elucidating the active biochemical control of proton release in the PRP I pathway. The PRP I is also proposed in the S_3_ to S_0_ transition (Greife et al. 2023; Bhowmick et al. 2023). Thus, water wires discovered by HR XRD (Umena et al. 2011) in the S_1_ state are indispensable for understanding of dynamical variations of the bio-molecular system structures for proton release in the S_2_ and S_3_ states at room temperature revealed by SFX XFEL (Okamoto et al. 2021; Hussein et al. 2021).

Several theoretical studies (Sakashita et al. 2017; Pantazis 2018; Greife et al. 2023) have been also performed to elucidate dynamical motions of the amino acid residues such as Asp61, Glu65, etc. in the S_2_ to S_3_ transition. Theoretical investigations of proton dynamics in PRP I (Greife et al. 2023) were crucial for understanding and explanation of the gateway for proton release in the S_2_ to S_3_ transition. Mutations of key amino acid residues have been performed to elucidate structure and function relationships in PRP I consisted of two channels D1 (Cl1-I_a_) and D2 (Cl1-I_b_). Large-scale QM(380 atoms)/MM computational results (Shoji et al. 2015b) have been discussed in relation to available experimental results, elucidating important roles of PRP I for proton release for water oxidation in OEC of PSII. PRP I is connected with the peripheral membrane protein PsbO of PSII of cyanobacteria (Umena et al. 2011). Figure 15 illustrates structural foundations of the channels D1 and D2 in Fig. 14.

### Proton release pathway II (PRP II)

**Fig. 16:**
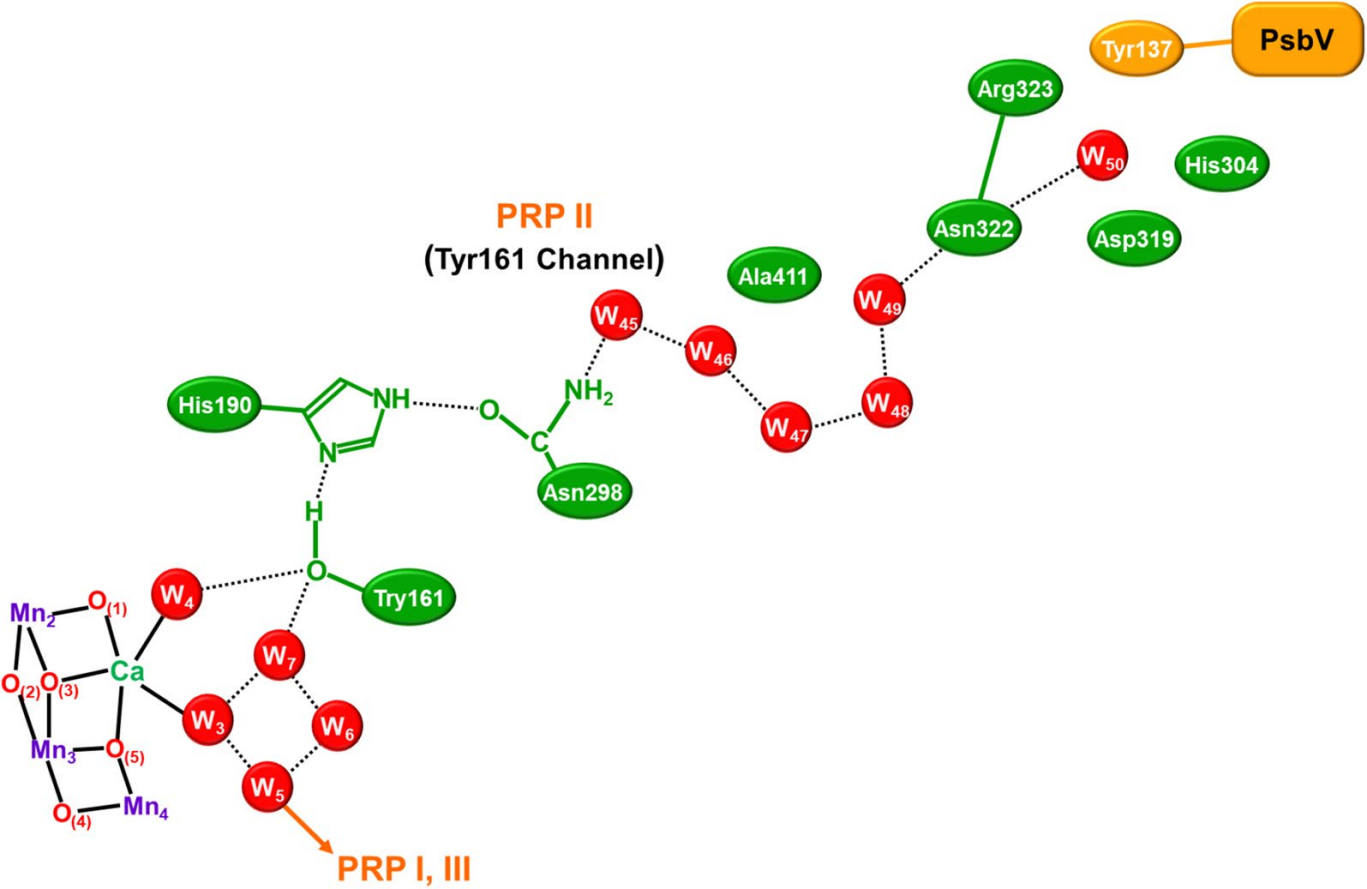
Hydrogen bonding networks for proton release pathway II (PRP II) proposed by HR XRD results (Umena et al. 2011), which is connected with the peripheral membrane protein PsbV. The large-scale QM(380 atoms)/MM computations (Shoji et al. 2015b) have been performed for refinements and confirmations of the HR XRD structure adding invisible hydrogen atoms in PRP II (Y_Z_-channel). Details of amino acid residues in PRP II are given in the text

The HR XRD result (Umena et al. 2011) has elucidated the proton release pathway II (PRP II) relating to Tyr161-OH as illustrated in Fig. 14. Tyr161-OH is hydrogen bonded with W4 and W7, indicating the connection with PRP I and III through the four-membered ring (W7, W6, W5, and W3). Tyr161-OH is also hydrogen-bonded with His190 as shown in Fig. 16. The strong hydrogen bond between Tyr161-OH and His190 provides the transient proton-transferred complex, Tyr161-O· (H^+^-His190) formed with light irradiation in the initial step of each Kok cycle in Fig. S3. His190 is further hydrogen-bonded with Asn298, which is connected with the water wire (W45, W46, W47, W48, C = O (backbone (BB) of Ala411)-W49-C = O (BB of Asn322)-W50 which is connected with the peripheral membrane protein PsbV in the case of cyanobacteria (Shen 2015; Shoji et al. 2015b).

Importantly, the water chain is linked with the charged amino acid residues, (Asn322)- W50-Asp319-Arg323-Asp315, indicating proton release pathway II (PRP II) protected with PsbV- Tyr137, namely peripheral membrane protein PsbV (Umena et al. 2011; Shen 2015; Shoji et al. 2015b). Recent mutation experiments of these residues elucidated the decrease of oxygen evolution activities: 63 ~ 91% as compared with that of the wild type (Zhu et al. 2022). On the other hand, Phe300, Asn301, Phe302, Asn303, His304, and Ala318 form a hydrophobic pocket for protection of release of proton (see Fig. A9-b in the paper by Shoji et al. (2015b)). The water wire is also protected by the α-helix structure consisted of His332, Met331, Val330, Glu329, Met328, Gly327, Leu326, and Asn325 (see Fig. A9-c by Shoji et al. 2015b).

Thus, the hydrogen bonding networks consisted of water molecules (W1 ~ W56) first observed by HR XRD (Umena et al. 2011) in the dark stable state of OEC of PSII exist commonly in the S_3_ state revealed by XFEL SFX experiments (Kern et al. 2018; Suga et al. 2019; Hussein et al. 2021). The original notations of water molecules (W5 ~ W27) around **1** confirmed by the large-scale QM(380 atoms)/MM computations are applicable to lucid understanding and natural explanations of the hydrogen- bonding networks connecting with W1 ~ W4 and the O_(1)_ and O_(4)_ sites defined by HR XRD (Umena et al. 2011), which are commonly observed in the S_1_, S_2_, and S_3_ states. We have used our notations of water molecules in PRP II (Shoji et al. 2015b).

### Proton storing and/or proton release pathway III (PRP III)

**Fig. 17:**
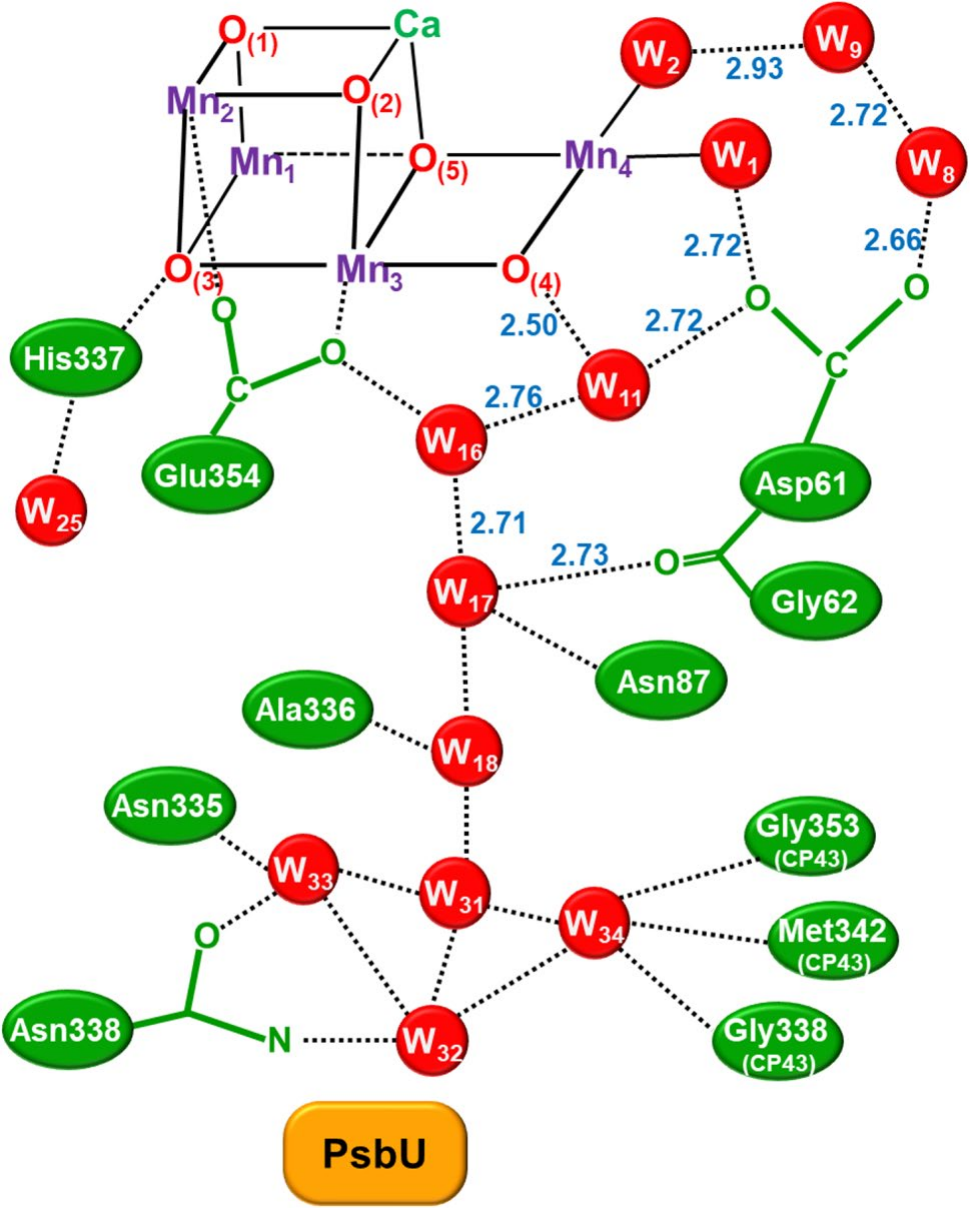
Hydrogen bonding networks for the proton storing and/or proton release pathway III (PRP III) by the large-scale QM(380 atoms)/MM computations (Shoji et al. 2015a, b). Eigen complex (EC) consisted of W31-W34 may be regarded as a proton storing complex after closing hydrogen bonding network linked with PsbU (Shoji et al. 2019a)

HR XRD results (Umena et al. 2011) have elucidated the water wire connecting with the O_(4)_ site of **1**, which is hydrogen-bonded with W11. Asp61 in PRP I is also linked with W11, which is linked with proton storing (PSP) and/or proton release pathway III (PRP III) as shown in Fig. 17 (Shoji et al. 2015b, 2021). Therefore, the Asp61 site has been assumed to be a base (B) for proton trapping in the deprotonation of W2 via the W9-W8-Asp61-W11-PRP III as shown in the QM/MM modeling (Shoji et al. 2015b). W11 is linked with the network; W16-W17-W18-Eigen complex (EC) consisted of four water molecules (H_9_O_4_)^+^ (W31,W32,W33, and W34) (Shoji et al. 2021). We have examined the HR XRD structure in detail, elucidating that the reaction pathway behind EC is closed for proton release in the S_1_ state. Therefore, the Eigen complex (EC; O_4_H_9_^+^) linked with Asp61 is supposed to be used for storing of proton arising from deprotonation of W2 in the S_1_ to S_2_ transition (Yamaguchi et al. 2022a). Ab initio molecular dynamics (Nakamura et al. 2019) using force fields obtained by UCC SD(T) (Schran et al. 2020) will be desirable for quantitative investigation of quantum shift of proton nuclei (nuclear motion in Fig. 2).

XEEL SFX experiments (Kern et al. 2018; Suga et al. 2019) have elucidated that W16 becomes invisible after the S_1_ to S_2_ transition, indicating enhanced dynamical motions of W16 in the linked hydrogen bonding network protected by Asp87-Arg-357-Gln165-W4 skeleton. Ser167-Ser169-Asp170 part is also linked with W11-W16 and W19-W5, suggesting complex dynamical behaviors in the S_2_ ~ S_4_ states for water oxidation. Recent XFEL SFX (Hussein et al. 2021) has elucidated the strong hydrogen bonding between Ser169 and W11 and between Ser169 and W1. Therefore, PSP/PRP III is often referred to as the O_(4)_ channel. Saito et al. have regarded the O_(4)_ channel as the proton release pathway in the S_0_ to S_1_ transition since PRP III with structural deformation for proton release can be connected with the peripheral membrane protein PsbU (Saito et al. 2015).

### Possible role of Channels E1 and E2

The HR XRD results have suggested the E1 and E2 channels near Cl2 ion as illustrated in Fig. 2. The hydrogen bonding networks, E1 and E2, near Cl2 were also examined by the QM/MM optimizations. Proton transfers via proton jump mechanism from the CaMn_4_O_5_ cluster to channels E1 and E2 through hydrogen bonding networks were prevented by the existence of Phe339 and Pro340 (see Fig. A10A in Shoji et al. (2015b)). Moreover, the hydrogen bonding network starting from W11 (PSP or PRP III) is not connected to the channel E1. However, the structure of His337-Asn338-Phe-Pro340 chain is similar to that of proton moving channel in cytochrome *c* oxidase (C*c*O) (Wikström et al. 2018; Shimada et al. 2023). The QM computation (Yoshioka et al. 2003) indicate an important role of water molecule hydrogen bonded with Tyr244 in C*c*O, indicating the necessity of QM/MM/MD modeling for conformational changes of the chain for the proton transfer. Recently, QM/MM/MD computations (Nakamura et al. 2019) were performed to elucidate the dynamical structural change of Asn338 for proton release via E1 channel. Thus, several conformational gateways may be conceivable in OEC of PSII.

### Classical and quantum MD simulations

The HR XRD (Umena et al. 2011) have elucidated that the OEC of PSII exhibits a multi-layer structure consisted of the catalytic CaMn_4_O_5_ core (**1**), first coordination shell consisted of seven amino acid residues, hydrogen bonding networks, etc. and peripheral membrane proteins, PsbO, PsbV, and PsbU, as shown in Figs. 14, 15, 16, 17. Large-scale QM(380 atoms)/MM calculations (Shoji et al. 2015a, b) starting from HR XRD have been effective and powerful for static geometry optimizations of the hydrogen bonding networks of OEC of PSII in the dark stable state. The WIP and three PRP investigated by the QM/MM (Shoji et al. 2015a,b) are very important for understanding of several proposals for water insertion in the S_2_ to S_3_ transition (Askerka et al. 2015; Capone et al. 2015, 2016; Retegan et al. 2016; Wang et al. 2017; Siegbahn 2018; Okamoto et al. 2021; Hussein et al. 2021). These proposals are summarized in the supporting section SII Part III in this review.

Classical molecular dynamics (CMD) simulations of OEC of PSII have been performed to elucidate functional water networks in OEC of PSII (Vassiliev et al. 2012; Ogata et al. 2013; Guerra et al. 2018; Sirohiwal and Pantazis 2022). The all-atom CMD simulations have indicated that the channel structures around the CaMn_4_O_5_ cluster in OEC of PSII are stable, elucidating three important channel structures; (1) proton release pathway 1 from Tyr161 to PsbV, (2) proton release pathway 2–1 and 2–2 from Asp61 to PsbO, and (3) proton release pathway 3 from W11 to PsbU (Ogata et al. 2013). Therefore, these pathways 1, 2–1 and 2–2, and 3 are consistent with the PRP II (Y_z_-channel) in Fig. 16, PRP I (D1 and D2 in Cl1 channel) in Fig. 15 and PRP III (O4 channel) in Fig. 17. The CMD simulations have indeed elucidated a dynamic proton transfer coupled with the molecular deformations via the PRP I (Guerra et al. 2018). Very recently, the CMD simulations (Sirohiwal and Pantazis 2022) have elucidated five water inlet pathways, indicating that the pathways I and V revealed are consistent with the WIP (A, B, and C) in Fig. 14. Thus, the all-atom CMD simulations have provided both static and dynamical information on the molecular-machine architecture of OEC of PSII (Ferreira et al. 2004; Umena et al. 2011), supporting the QM/MM results around the CaMn_4_O_5_ cluster in Figs. 14–17 (Shoji et al. 2015a, b).

Over past decades, classical and quantum nature of proton and electron transfers in the hydrogen bonding networks have been investigated by the QM/MM/MD computations (Marcus 1956; Moser et al. 1992; Marx et al. 1999; Shigeta et al. 1999; Nakamura et al. 2019; Capone et al. 2020; Saito and Takano 2021). Excellent reviews have also been published to summarize recently developed QM/MM/MD methods (Curchod and Martinez 2018; Crespo-Otero and Barbatti 2018). However, applications of these advanced methods to OEC of PSII remain as future interesting tasks (see SI Part IX).

## Computational results for the S_3_ intermediates by beyond hybrid DFT results

### Computational models for geometry optimizations of the CaMn_4_O_5_ cluster in the Kok cycle

**Fig. 18:**
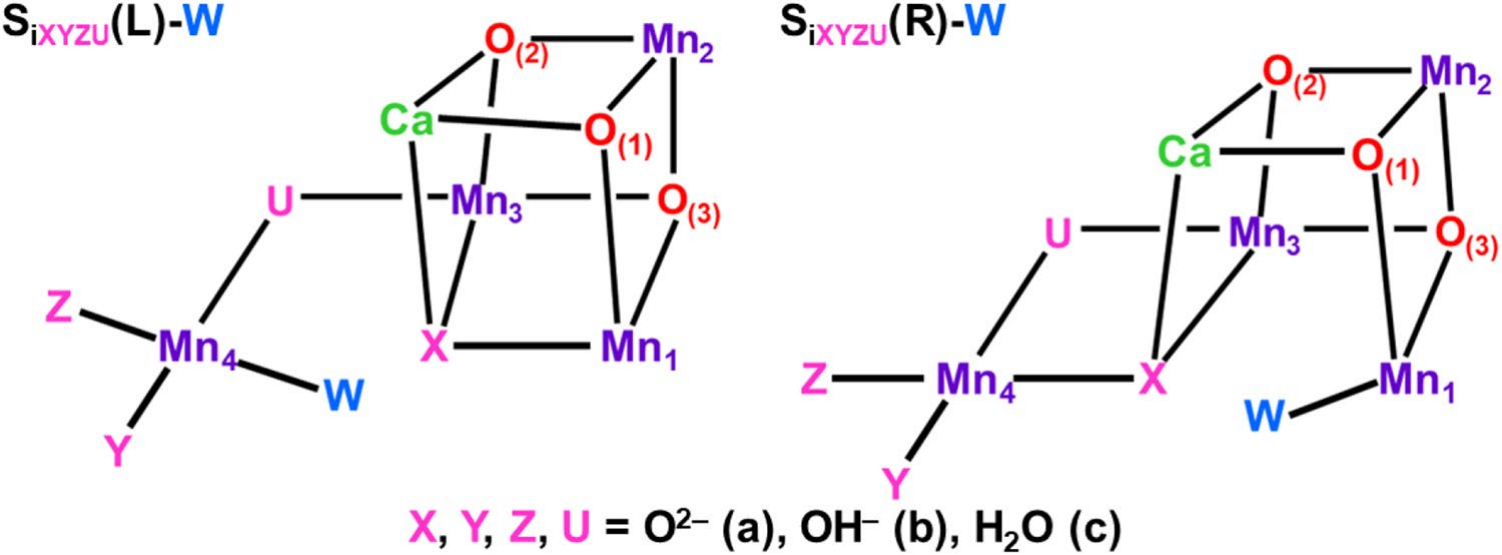
Computational models for the left (L)- and right (R)-opened structures of the CaMn_4_O_x_ cluster in the S_i_ (*i* = 1 ~ 3) state, where variations of oxidation states of water molecules at X = O_(5)_, Y = W2, Z = W1, and U = O_(4)_ sites are denoted as a = O^2−^, b = OH^−^, and c = H_2_O, respectively. W denotes the inserted water molecule (a, b, or c) in the S_3_ and S_4_ states of the Kok cycle

Our computational schemes for theoretical investigation of **1** in the S_i_ (*i* = 1 ~ 3) state of the Kok cycle are consisted of three procedures: (I) Full geometry optimizations of possible intermediates in each Kok cycle by the UB3LYP-D3 method, (II) Elucidation of relative energies among the possible intermediates by several hybrid DFT (HDFT) methods, and (III) Examination of the computational results in the step II by beyond HDFT methods. Beyond HDFT methods are classified into the couple cluster (CC) and configuration interaction (CI) types, considering single (S), double (D) and triple (T) excitations for electron correlation corrections (see details in SI Part I). In this review, we have performed CCSD(T_0_) computations using domain-based local pair natural orbitals (DLPNO) (Riplinger and Neese 2013; Saitow et al. 2017; Neese 2018). To this end, DLPNO have been constructed with natural orbital (UNO) and/or localized natural orbitals (ULO) of the UB3LYP solution (Yamaguchi 1979; 1980; 1990a). As shown in Table 1, the QM (380 atoms)/MM computations (Shoji et al. 2015b) have been performed for full geometry optimizations of whole proton-shifted isomers from a parent structure in each S_i_ (*i* = 1 ~ 3) state as illustrated by X, Y, Z, and U of **1** in Fig. 18 since the oxidation states of the water molecule (oxygen dianion O^2−^ (= a), hydroxide anion OH^−^ (= b), and H_2_O (= c)) in **1** have not been discriminated on the experimental grounds.

We have assumed the parent neutral structure CaMn_4_O_5_(H_2_O)_4_ for **1** in the S_1_ state that is expressed as S_1acca_, namely X = U = O^2−^ and Y = Z = H_2_O in Fig. 18 (Kanda et al. 2011b). The oxygen dianions of O_(i)_ (*i* = 1 ~ 5) of **1** are possible candidates for proton acceptor from the proton donor of H_2_O at the W2 (= Y) or W1 (= Z) site, providing several proton-shifted structures from the parent structure as shown in Table 1. The QM(HDFT) level of theory was effective and reliable for geometry optimizations (step I), providing important information for structure assignments. However, relative energies among them (step II) are highly dependent on the weight of the Hartree–Fock (HF) exchange term in the HDFT model. Therefore, beyond HDFT computations (step III) become necessary for refined relative energies (Kawakami et al. 2018). Relative energies among possible intermediates in the S_i_ (*i* = 1, 2, 3) were indeed investigated by DLPNO CCSD(T_0_) methods (Miyagawa et al. 2019, 2020, 2021, 2022a, b). In this review, we mainly review the computational results for the S_3_ state on the basis of two different QM (103; 227 atoms) models because of the current interest on the S_3_ → [S_4_] → S_0_ transition (Bhowmick et al. 2023; Greife et al. 2023).

### Computational methods of relative energies in the S_3_ intermediates

The DLPNO CCSD(T_0_) computations are time-consuming for **1**. Therefore, they have been performed for small QM models (103 atoms; QM-I) because extended basis sets such as def2-TZVP are necessary for reliable CC computations (Miyagawa et al. 2022a, b). Here, DLPNO-CCSD(T_0_)/def2-TZVP computational results based on an extended QM model (227 atoms; QM-II) are examined for quantitative purpose. Relative stabilities among the S_3_ intermediates are also examined with four HDFT methods (step II); UB3LYP/def2-TZVP with the standard weight (*w*) of the Hartree–Fock (HF) exchange term: *w* = 20%, UB3LYP* with *w* = 15%, UB3LYP** with *w* = 10%, and UB3LYP*** with *w* = 5%. Double hybrid DFT (UB2PLYP) is also used as an extended HDFT model. Solvation effects for **1** were included by the CPCM empirical model (Tomasi et al. 2005) for all the computations instead of the MM model for the computational economy. Before the CC computations, we have performed full geometry optimizations (step I) of key S_3_ intermediates by UB3LYP-D3 using the extended def2-TZVP basis set for each S_3_ structure.

### Examinations of the optimized geometrical parameters

Scope and reliability of the optimized geometrical parameters are examined on the basis of the EXAFS (Yano and Yachandra 2014) results and experimental results for model complexes (Dau et al. 2008). The Mn–Mn and Mn–O distances for the model complexes in Figs. 3A-D and cubane-like clusters in Fig. 5 have been reported, providing important information for comparisons with geometrical parameters of the R-opened S_3_ intermediates summarized in Table 3. The optimized Mn_1_-Mn_2_, Mn_2_-Mn_3_, Mn_3_-Mn_4_, Mn_1_-Mn_3_, and Mn_1_-Mn_4_ distances for R-opened S_3_ intermediates are 2.76 ~ 2.80, 2.81 ~ 2.86, 2.73 ~ 2.95, 3.27 ~ 3.55, and 5.23 ~ 5.53 Å, respectively. The Mn_1_-Mn_2_ and Mn_2_-Mn_3_ distances are not so different among them, indicating the Mn(IV)-Mn(IV) distance of di-μ-oxo Mn dimer (Yachandra et al. 1996). On the other hand, the Mn_3_-Mn_4_ distances are variable, depending on the JT effect of the Mn(III) ion (Isobe et al. 2012; Shoji et al. 2019a). The Mn_3_-Mn_1_ distances are longer than 3.2 Å, indicating the R-opened structures. The Ca-Mn_i_ (*i* = 1 ~ 3) distances are 3.20 ~ 3.57 Å, whereas the Ca-Mn_4_ distance is 3.90 ~ 4.23 Å.

**Table 3:** The optimized geometric distances (Å) for several R-opened (open-cubane) water (W) inserted CaMn_4_O_x_-W intermediates in the S_3_ state by UB3LYP/def2-TZVP and by SFX-XFEL^a)^

Types	Exp.^b)^	OH	OH_glu_	Oxo	Oxyl	OH_w_	Perox
Mn_1_-Mn_2_	2.75	2.76	2.79	2.80	2.78	2.79	2.77
Mn_2_-Mn_3_	2.86	2.86	2.85	2.82	2.83	2.85	2.81
Mn_3_-Mn_4_	2.77	2.77	2.75	2.73	2.83	2.92	2.95
Mn_1_-Mn_3_	3.33	3.55	3.53	3.46	3.27	3.55	3.36
Mn_1_-Mn_4_	5.06	5.35	5.37	5.33	5.25	5.53	5.23
Ca-Mn_1_	3.37	3.34	3.37	3.20	3.27	3.46	3.39
Ca-Mn_2_	3.32	3.29	3.26	3.25	3.31	3.27	3.35
Ca-Mn_3_	3.52	3.51	3.30	3.37	3.35	3.38	3.57
Ca-Mn_4_	4.00	4.19	3.90	4.06	4.01	4.14	4.23
Mn_1_-O_(6)_	1.79	1.80	1.80	1.67	1.72	1.82	2.14
Mn_3_-O_(5)_	2.00	1.83	1.82	1.84	1.78	1.89	1.88
Mn_4_-O_(5)_	2.22	1.82	1.81	1.79	2.09	2.00	2.26
Ca-O_(5)_	2.60	2.85	2.56	2.67	2.55	2.61	2.97
Ca-O_(6)_	2.49	2.53	2.45	2.36	2.45	2.59	2.36
O_(6)_-O_(5)_	2.09	2.46	2.54	2.49	2.03	2.47	1.41

^a)^The molecular structures of the water (W) inserted CaMn_4_O_x_-W S_3_ intermediates are abbreviated as W in this table: OH = S_3abca_-OH(R), OH_glu_ = S_3abca_-OH_glu_(R), Oxo = S_3acca_-Oxo(R), Oxyl = S_3acca_-Oxyl (R), and Perox = S_3acca_-Peroxide(R). ^b)^The experimental values were taken from PDB ID (6DHO) (Kern et al. 2018)

The Mn(IV)_4_-O_(5)_ for the hydroxides and oxo intermediates is about 1.79 ~ 1.82 Å, whereas the Mn(III)_4_-O_(5)_ distance for the oxyl and peroxide intermediates is 2.09 ~ 2.26 Å, indicating the JT distortion. The observed distance of the Mn_4_-O_(5)_ bond by XFEL (Kern et al. 2018; Suga et al. 2019) is 2.22 Å, which accord with the JT effect of the Mn(III)_4_ ion, indicating that the XFEL structure is different from the Mn-hydroxide and Mn-oxo intermediates with the Ca(II)Mn(IV)_4_ clusters.

The Ca-O_(5)_ distance is 2.56 ~ 2.61 Å except for hydroxide (OH) and peroxide, indicating the coordination bond. The Ca-O_(6)_ distance is 2.36 ~ 2.59 Å for all the S_3_ intermediates, which accord with the strong coordination of the O_(6)_ site to the Ca ion, indicating the participation of the Ca ion for the O_(6)_-O_(5)_ bond formation (Yamaguchi et al. 2018, 2019; Shoji et al. 2019b). The O_(6)_-O_(5)_ distances are about 2.46 ~ 2.54 Å for all the intermediates except for the oxyl and peroxide intermediates, for which they are about 2.03 and 1.41 Å, respectively.

The observed Mn_4_-O_(5)_ distances by SFX XFEL results (Kern et al. 2018; Suga et al. 2019) are 2.26 (2.26, 2.28), 2.20 (1.93, 1.90), and 2.18 (1.80, 1.94) Å for the S_0_, S_1_, and S_2_ states, respectively, where the corresponding optimized vales by UB3LYP-D3 with def2-TZVP basis and the estimated values by the observed Mn_4_-Mn_3_ distances by SFX XFELs (Kern et al. 2018) are given in parentheses. No serious discrepancy is resulted for the S_0_ state. On the other hand, the observed values for the S_1_ and S_2_ states by SFX XFELs are longer 0.3 ~ 0.4 Å than the calculated and estimated values. The corresponding values for S_3acca_(R)-oxyl are 2.22 (2.09, 1.86) Å. The observed value is compatible with the optimized value but different from the estimated value using the observed Mn_4_-Mn_3_ distance, indicating that the estimated O_(6)_-O_(5)_ distance may become longer than 2.2 Å (Yamaguchi et al. 2022b).

Table 4 summarizes the optimized Mn–Mn, Ca-Mn, Mn–O, Ca-O, and O_(5)_-O_(6)_ distances for L-opened S_3_ intermediates. The optimized five Mn–Mn distances for them are 2.72 ~ 2.77, 2.76 ~ 2.77, 3.09 ~ 3.30, 2.87 ~ 3.31, and 4.98 ~ 5.44 Å, respectively. The Mn_1_-Mn_2_ and Mn_2_-Mn_3_ distances are not so different among them. On the other hand, the Mn_3_-Mn_4_ distances are longer than 3.1 Å for the L-opened structure. The Mn_3_-Mn_1_ distances are about 2.87 ~ 2.89 Å responsible for the closed-cubane structure in Fig. 3 except for the Mn-oxyl and peroxide intermediates, for which they are 3.07 and 3.31 Å, respectively. The Ca-Mn_i_ (*i* = 1 ~ 3) distances are 3.19 ~ 3.49 Å, whereas the Ca-Mn_4_ distance is 3.54 ~ 4.33 Å, indicating variations between the R- and L-opened structures.

**Table 4:** The optimized geometric distances (Å) for several L-opened (closed-cubane) water (W) inserted CaMn_4_O_x_-W intermediates in the S_3_ state by UB3LYP/def2-TZVP and by SFX-XFEL^a)^

Types	Exp.^b)^	OH	Oxo-L1	Oxo-L2	Oxyl-L1	Oxyl-L2	L-H_2_O	Perox
Mn_1_-Mn_2_	2.75	2.72	2.73	2.73	2.77	2.77	2.72	2.76
Mn_2_-Mn_3_	2.86	2.77	2.77	2.76	2.77	2.77	2.77	2.78
Mn_3_-Mn_4_	2.77	3.26	3.17	3.15	3.09	3.10	3.30	3.05
Mn_1_-Mn_3_	3.33	2.89	2.88	2.87	3.07	3.07	2.89	3.31
Mn_1_-Mn_4_	5.06	5.26	4.98	5.01	5.13	5.15	5.44	5.11
Ca-Mn_1_	3.37	3.39	3.35	3.43	3.43	3.43	3.37	3.51
Ca-Mn_2_	3.32	3.42	3.40	3.49	3.40	3.39	3.40	3.41
Ca-Mn_3_	3.52	3.31	3.19	3.29	3.30	3.31	3.30	3.48
Ca-Mn_4_	4.00	4.18	3.54	3.62	3.82	3.89	4.33	3.94
Mn_1_-O_(5)_	2.22	1.84	1.81	1.81	2.15	2.16	1.86	2.30
Mn_3_-O_(5)_	2.00	1.89	1.87	1.87	1.81	1.81	1.90	1.90
Mn_4_-O_(6)_	1.79	1.79	1.70	1.70	1.71	1.72	2.06	2.13
Ca-O_(5)_	2.60	2.49	2.47	2.55	2.48	2.49	2.49	2.75
Ca-O_(6)_	2.49	2.47	2.34	2.35	2.61	2.69	3.72	2.39
O_(6)_-O_(5)_	2.09	2.51	2.57	2.53	2.03	2.03	2.54	1.42

^a)^The molecular structures of the water (W) inserted CaMn_4_O_x_-W S_3_ intermediates are abbreviated as W in this table: OH = S_3abca_−OH(L), OH_glu_ = S_3abca_−OH_glu_(L), Oxo = S_3acca_−Oxo(L), Oxyl = S_3acca_−Oxyl (L), and Perox = S_3acca_−Peroxide(L). ^b)^The experimental values were taken from PDB ID (6DHO) (Kern et al. 2018)

The Mn(IV)_4_-O_(5)_ calculated for the Mn-hydroxides and -oxo intermediates is about 1.81 ~ 1.86 Å, whereas the Mn(III)_4_-O_(5)_ distances for the Mn-oxyl and -peroxide intermediates are 2.15 and 2.30 Å, respectively, which accord with the JT effect. The Ca-O_(5)_ distance is 2.47 ~ 2.55 Å except for peroxide, indicating the coordination bond. The Ca-O_(6)_ distance is 2.34 ~ 2.69 Å for all the S_3_ intermediates except for Mn-H_2_O, which accord with the strong coordination of the O_(6)_ site to the Ca ion, indicating particular importance of the cubane structure, CaMn_3_O_4_ (Yamaguchi et al. 2019). The O_(6)_-O_(5)_ distances are 2.51 ~ 2.55 Å for all the intermediates except for the Mn-oxyl and -peroxide intermediates, for which they are 2.03 and 1.42 Å, respectively. Judging from the Mn_3_-Mn_4_ and Mn_1_-Mn_3_ distances, the L-opened oxyl intermediate is different from the observed S_3_ intermediate for cyanobacteria by SFX XFEL (Kern et al. 2018; Suga et al. 2019). The UB3LYP-D3/def2-TZVP method works well for elucidation of geometric characteristics of the L-opened S_3_ intermediates in OEC of PSII. Thus, full geometry optimizations by UB3LYP-D3/def2-TZVP level of theory are handy and powerful for elucidation of geometric characteristics of both R- and L-opened S_3_ intermediates in OEC of PSII as shown in Tables 3 and 4.

### Relative stabilities among the S_3_ intermediates by HDFT and DLPNO CCSD(T_0_)

**Fig. 19:**
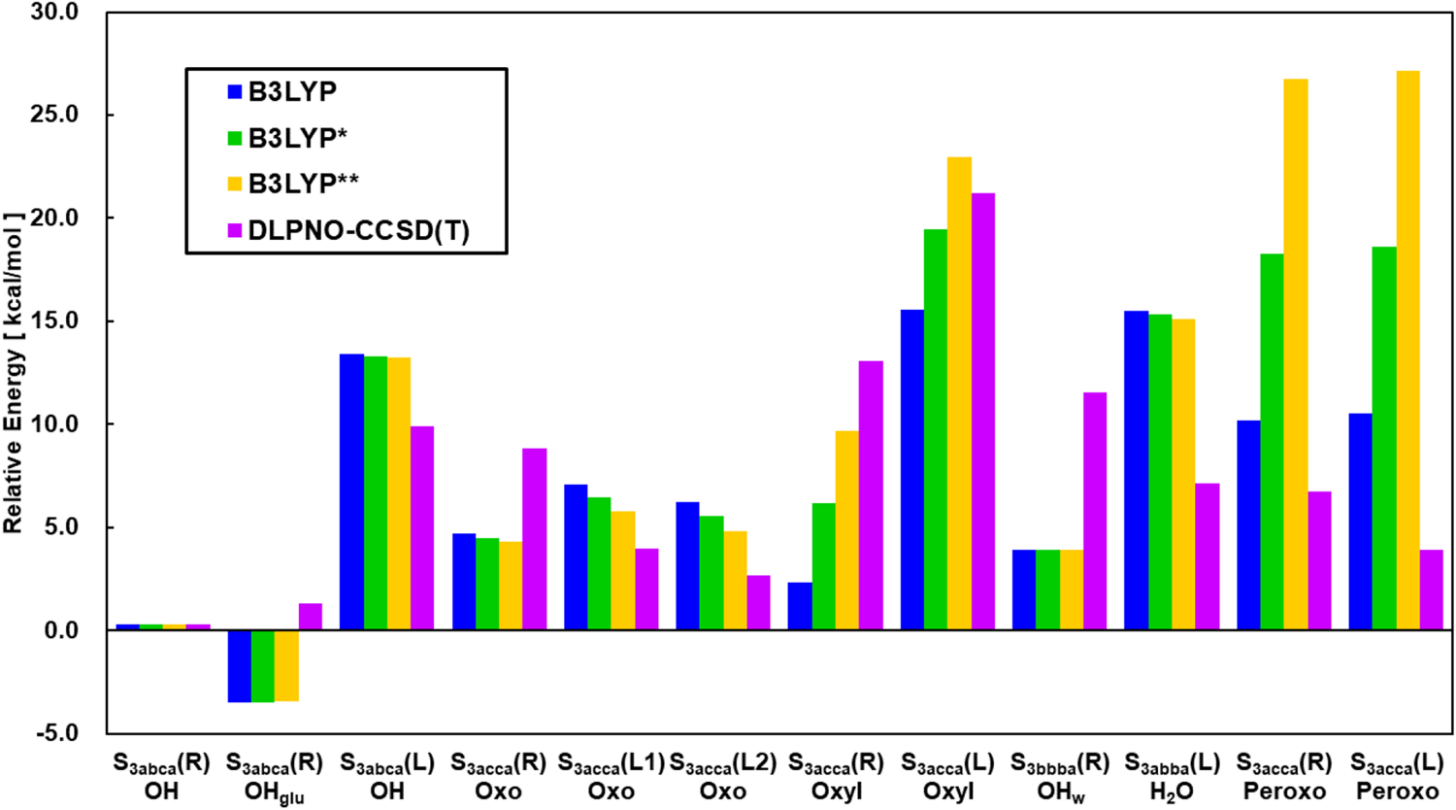
Relative energy among the twelve S_3_ intermediates by UB3LYP (*w* = 20%), UB3LYP* (*w* = 15%), UB3LYP** (*w* = 10%), and normal PNO (n-) DLPNO-CCSD(T) based on the intermediate QM (227 atoms) models

As mentioned above, DLPNO CCSD(T_0_) calculations have already been performed for elucidation of relative stabilities among possible intermediates on the basis of relatively small quantum mechanical (QM) model (103 atoms; QM-I model) for the S_3_ state (Miyagawa et al. 2022a, b) as shown in the supporting material. However, amino acid residues in the second coordination shell and key water molecules are not included in the small QM models (QM-I model). Here, the DLPNO CCSD(T_0_) computations are performed for the extended QM model of **1** involving partly the hydrogen bonding networks; 226 + O_(6)_ (or O_X_) atoms; QM-II model). DLPNO CCSD(T_0_) computations are performed for twelve S_3_ intermediates formed just before the O–O bond formation, elucidating relative energies among them. Relative energies between the reaction intermediates by DLPNO CCSD(T_0_) have provided important information for elucidation of scope and reliability of those by HDFT computations. Figure 19 illustrates the computational results. Table 5 summarizes relative energies of the S_3_ intermediates based on the QM-II model. Following conclusions are obtained from Fig. 19 and Table 5.

1. The S_3abca_(R)-OH_glu_ was the most stable among three hydroxide-inserted structures by the UB3LYP (*w* = 10 ~ 20%) and CC computations based on the QM-I. However, S_3abca_(R)-OH becomes more stable by a few kcal/mol than S_3abca_(R)-OH_glu_ by normal PNO- (*n*-) DLPNO-CCSD(T_0_) based on the QM-II although the tendency is not changed by the UB3LYP (*w* = 10 ~ 20%). The S_3abca_(L)-OH is unstable by both the computations based on QM-II in a sharp contrast with QM-I, for which the R- and L-S_3abca_(R)-OH isomers are nearly degenerated in energy, indicating the importance of a realistic QM model for OEC of PSII. The computational results by QM-II were indeed consistent with the EPR results for the R-opened hydroxide (Cox et al. 2014).
2. The energy gap between S_3abca_(R)-OH and S_3bbba_(R)-OH_w_ was smaller than 5 kcal/mol by both the calculations based on the QM-I. It remains to be smaller than 5 kcal/mol by UB3LYP (*w* = 10 ~ 20%)/QM-II. On the other hand, the gap becomes larger than 10 kcal/mol by *n*-DLPNO CCSD(T_0_)/QM-II.
3. The energy gap between S_3abca_(R)-OH and S_3abba_(L)-H_2_O was almost zero by tight PNO- (*t-*) DLPNO- CCSD(T_0_)/QM-I although it was larger than 5 kcal/mol by the UB3LYP (*w* = 10 ~ 20%). On the other hand, it becomes larger than 5 (15) kcal/mol by *n*-DLPNO-CCSD(T_0_) (UB3LYP (*w* = 10 ~ 20%)) based on the QM II model, suggesting that specific conditions such as the high pH are necessary for stabilization of S_3abba_(L)-H_2_O.
4. The energy gap between S_3abca_(R)-OH and S_3abca_(L1)-oxo (S_3abca_(L2)-Oxo) was very small by *t*-DLPNO CCSD(T_0_)/QM-I in contrast to the larger gaps than 5 kcal/mol by UB3LYP (*w* = 10 ~ 20%). It is still smaller than by 5 kcal/mol by *n*-DLPNO CCSD(T_0_)/QM-II, although UB3LYP (*w* = 10 ~ 20%)/QM-II predicts the larger gap than 5 kcal/mol.
5. The energy gap between S_3abca_(R)-OH and S_3acca_(R)-oxo was larger than 10 kcal/mol by both the calculations based on the QM-I. The gap also remains to be 9 kcal/mol by *n*-DLPNO CCSD(T_0_)/QM-II although it becomes smaller than 5 kcal/mol by UB3LYP (*w* = 10 ~ 20%)/QM-II.
6. The energy gap between S_3abca_(R)-OH and S_3abca_(R)-oxyl (S_3abca_(L)-oxyl) was larger than 15 kcal/mol by the calculations based on the QM-I. It remains to be larger than 10 kcal/mol by *n*-DLPNO CCSD(T_0_)/QM-II. On the other hand, it becomes smaller than 5 kcal/mol by UB3LYP (*w* = 20%)/QM-II although it increases with the decrease of the *w*-value. Therefore, the gap in turn becomes almost zero at UB3LYP (*w* = 25%). Thus, the relative stability of the S_3abca_(R)-oxyl is highly dependent on the w-value of HDFT, which accord with previous results (Isobe et al. 2016; Yamaguchi et al. 2018). On the other hand, the corresponding gap for S_3abca_(L)-oxyl is larger than 15 kcal/mol by all the computational methods based on QM-II.
7. The energy gap between S_3abca_(R)-OH and S_3acca_(R)-peroxide was larger than 10 kcal/mol by *t*-DLPNO CCSD(T_0_)/QM-I. It remains to be larger than 5 kcal/mol by *n*-DLPNO CCSD(T_0_)/QM-II. On the other hand, it is larger than 20 (10) kcal/mol by UB3LYP (*w* = 10 ~ 20%)/QM-I (QM-II), indicating a significant size effect. Formation of S_3acca_(R)-peroxide is hardly possible in the S_3_ state.
8. The energy gap between S_3abca_(R)-OH and S_3acca_(L)-peroxide was few kcal/mol by *t*-DLPNO CCSD(T_0_)/QM-I. It remains to be smaller than 5 kcal/mol by *n*-DLPNO CCSD(T_0_)/QM-II. On the other hand, it is larger than 20 (10) kcal/mol by UB3LYP (*w* = 10 ~ 20%)/QM-I (QM-II), indicating significant difference between the CC and HDFT methods.

**Table 5:** Relative energies (kcal/mol) among twelve S_3_ intermediates by DLPNO CCSD(T_0_)/ def2-TZVP methods

Types/Methods^a)^	B3	B3*	B3**	B3***	B3D3	B3*D2	B2P	DLPNO
S_3abca_(R)-OH	0.00	0.00	0.00	0.00	0.00	0.00	0.00	0.00
S_3abca_(R)-OH_glu_	− 3.49	− 3.48	− 3.44	− 3.42	− 1.32	− 0.23	− 0.74	1.32
S_3abca_(L)-OH	13.4	13.3	13.3	13.1	9.22	8.79	11.1	9.88
S_3abca_(R)-oxo	4.71	4.50	4.30	4.13	9.91	9.82	13.1	8.85
S_3abca_(L1)-oxo	7.06	6.44	5.78	5.09	9.84	8.76	11.6	3.97
S_3abca_(L2)-oxo	6.24	5.55	4.83	4.08	9.76	7.73	10.2	2.64
S_3abca_(R)-oxyl	2.33	6.19	9.65	12.5	11.6	17.8	16.0	13.1
S_3abca_(L)-oxyl	15.5	19.5	23.0	25.8	21.8	25.0	28.0	21.2
S_3bbba_(R)-OH_w_	3.89	3.89	3.90	3.89	4.28	5.99	9.73	11.5
S_3abba_(L)-H_2_O	15.5	15.3	15.0	14.8	7.97	7.55	12.8	7.16
S_3acca_(R)-peroxide	10.2	18.2	26.8	35.2	10.3	22.1	23.2	6.75
S_3acca_(L)-peroxide	10.5	18.6	27.1	35.5	10.6	21.5	23.0	3.90

^a)^The abbreviations of the method indicate *B3* B3LYP, B3* B3LYP*, B3** B3LYP**, B3*** B3LYP***, *B3D3* B3LYP-D3, B3*D2 B3LYP*-D2, *B2P* B2PLYP, *DLPNO* DLPNO CCSD(T_0_), respectively

From the conclusions (1)–(8), the relative stabilities among the S_3_ intermediates are highly dependent on the computational methods and sizes of the QM models employed. The S_3abca_(R)-OH was calculated to be the most stable among the S_3_ intermediates at *n*-DLPNO CCSD(T_0_)/QM-II level of theory. As mentioned above, this is consistent with the conclusion obtained by the EPR result (Cox et al. 2014) for the S_3_ state at a low temperature. In fact, the (4444) valence state and projection factors for S_3abca_(R)-OH (and/or S_3abca_(R)-OH_glu_) revealed by the exact diagonalization of the effective spin Hamiltonians (Isobe et al. 2014, 2019; Miyagawa et al. 2022b) are compatible with the observed EPR results (Cox et al. 2014). On the other hand, the S_3abca_(R)-oxyl intermediate is less stable by about 18 and 13 kcal/mol than S_3abca_(R)-OH by *t*-DLPNO CCSD(T_0_)/QM-I and *n*-DLPNO CCSD(T_0_)/QM-II, respectively, indicating the reduction of about 5 kcal/mol by the extension of the QM model from I to II. Therefore, formation of the Mn-oxyl intermediate is not favorable in the S_3_ state of the Kok cycle at present DLPNO CCSD(T_0_) level of theory.

## Discussions and concluding remarks

### Possible mechanisms of water oxidation in OEC of PSII

The DLPNO CCSD(T_0_) computations for the S_3_ intermediates have elucidated the greater stability of the Mn-hydroxide intermediate, S_3abca_(R)-OH with the uniform valence state (4444), than Mn-oxyl/oxo intermediate, S_3acca_(R)-O· with the formal valence state (4443). The exact diagonalization of the spin Hamiltonian models (Isobe et al. 2019; Miyagawa et al. 2022a, b) have elucidated the *S* = 3 and *S* = 6 ground states for these intermediates in accord with the EPR results (Cox et al. 2014; Boussac et al. 2018; Boussac 2019). Therefore, the O–O bond formation for water oxidation occurs after the third flash in the Kok cycle, namely S_3_ → (h*v*) → [S_4_] → S_0_ (Bhowmick et al. 2023; Greife et al. 2023).

Very recently, time-resolved (TR) XFEL SFX experiments (Bhowmick et al. 2023) have been performed to elucidate sequential steps of the above transition. According to the TR XFEL SFX results, the formation of Tyr-O radical occurs within 50 μs after the third flash as shown in Eq. (9a). However, its lifetime is considerably long. The next step observed (Bhowmick et al. 2023) is early proton release from **1** to lumen within 350–500 μs as shown in Eq. (9b). In our model, the proton source (Isobe et al. 2012; Shoji et al. 2015b, c, 2017) is considered as water molecule at the W1 (Z) site, which is connected with Asp61 and proton release pathway (PRP) I in Fig. 15 (Greife et al. 2023). TR XFEL SFX (Bhowmick et al. 2023) have revealed the dynamical mechanism of the proton release along the PRP I assisted with complex dynamical motions (conformational gating in our terminology) of Glu65, Glu312 and water molecules (W12–W14). Interplay between TR FTIR and QM/MM/MD computations (Greife et al. 2023) have also elucidated dynamical mechanisms of proton release in PRP I.

9a $${\text{Tyr}} - {\text{OH }} + {\text{ S}}_{{\text{3abca}}} \left( {\text{R}} \right) - {\text{OH }}\left[ {{4444}} \right] \to \left( {{\text{h}}v} \right) \to {\text{Tyr}} - {\text{O}} \bullet \, \left( {{\text{H}}^+ - {\text{His}}} \right) \, + {\text{ S}}_{{\text{3abca}}} \left( {\text{R}} \right) - {\text{OH }}\left[ {{4444}} \right]$$

9b $${\text{Tyr}} - {\text{O}} \bullet \, \left( {{\text{H}}^+ - {\text{His}}} \right) \, + {\text{ S}}_{{\text{3abca}}} \left( {\text{R}} \right) - {\text{OH }}\left[ {{4444}} \right] \to {\text{Tyr}} - {\text{O}} \bullet \, \left( {{\text{H}}^+ - {\text{His}}} \right) \, + {\text{ S}}_{{\text{3abba}}} \left( {\text{R}} \right) - {\text{OH }}\left[ {{4444}} \right] \, + {\text{ H}}^+_{{\text{lumen}}}$$

The next step is the O–O bond formation for water oxidation. We have classified the process into three cases (Isobe et al. 2016, 2021, 2022; Miyagawa et al. 2022b); (A) the O–O bond formation before the reduction of Tyr161-O radical, (B) the O–O bond formation coupled with the reduction of Tyr161-O radical, and (C) the O–O bond formation after the reduction of the Tyr161-O radical. The mechanism (A) is formally written by Eq. (9c), providing the Mn-peroxide with the O_(5)_-O_(6)X_ bond and the valence state (3443), followed by OET to Tyr-O radical as shown in Eq. (9d). Therefore, the Mn(V) site is not formed in the mechanism (A) (see details in supporting information SVI). Recent XES experiments (Davis et al. 2018) have indeed elucidated no formation of Mn(V) site, giving a proposal of the O–O bond formation before the OET to Tyr-O radical. Type A pathways were examined on the theoretical grounds (Isobe et al. 2021, 2022; Corry and O'Malley 2018). The very recent TR XFEL SFX experiments (Bhowmick et al. 2023) have elucidated that the O–O bond formation and the reduction of the Tyr-O radical occur in a parallel manner in the case of cyanobacteria at room temperature. Therefore, the formation step of the superoxide anion radical followed by rapid O_2_ evolution and one-electron reduction of Tyr-O radical may occur in almost parallel manner (Bhowmick et al. 2023) in the case of the A-mechanism.

9c $${\text{Tyr}} - {\text{O}} \bullet \, \left( {{\text{H}}^+ - {\text{His}}} \right) \, + {\text{ S}}_{{\text{3abba}}} \left( {\text{R}} \right) - {\text{OH }}\left[ {{4444}} \right] \to {\text{Tyr}} - {\text{O}} \bullet \left( {{\text{H}}^+ - {\text{His}}} \right) \, + {\text{ S}}_{{\text{3abca}}} \left( {\text{R}} \right) - {\text{Peroxide }}\left[ {{3443}} \right]$$

9d $${\text{Tyr}} - {\text{O}} \bullet \, \left( {{\text{H}}^+ - {\text{His}}} \right) \, + {\text{ S}}_{{\text{3abca}}} \left( {\text{R}} \right) - {\text{Peroxide }}\left[ {{3443}} \right] \to {\text{Tyr}} - {\text{OH}}\left( {{\text{His}}} \right) \, + {\text{ S}}_{{\text{3abca}}} \left( {\text{R}} \right) - {\text{Supereroxide }}\left[ {{3443}} \right]$$

On the other hand, the Mn(V) site is formally formed in the mechanism (C), providing the high- valent Mn(V) = O bond of the S_4abca_(R)(5444) = O intermediate in Eq. (10a) under a condition that the coordination to the Ca ion plays an important role for quenching of the oxyl-radical character as shown in Eq. (3) (Yamaguchi et al. 2007, 2019; Miyagawa et al. 2022a, b). The electrophilic addition of second water (H_2_O_(7)_) (or HO_(7)_ anion) to the Mn(V)_1_ = O_(6)_ bond may become feasible, providing Mn-Hydroperoxide, S_4acca_(R)-O_(6)_O_(7)_H, as shown in Eq. (10b) (Yamaguchi et al. 2019; Miyagawa et al. 2022a, b) (see supporting information). It is noteworthy that this type of the OOH bond formation entails one-electron reductions of the Mn(V)_1_ and Mn(IV)_4_ sites, providing the (4443) configuration in a sharp contrast to two-electron reduction from Mn(V)_1_ to Mn(III)_1_ responsible for the AB mechanism in Eq. (3) (Iwata and Barber 2004).

The concerted bond switching (CBS) mechanism for the O_(5)_–O_(6)_ bond formation (Shoji et al. 2019b) is also feasible by the participations of the Ca ion and His332 to give Mn-Peroxide with the O_(5)_–O_(6)X_ bond and (4443) valence state via one-electron reductions from Mn(V)_1_ to Mn(IV)_1_ and from Mn(IV)_4_ to Mn(III)_4_ as illustrated in Eq. (10c) (see supporting information). On the other hand, the same peroxide is formed via the radical coupling (RC) mechanism of the Mn-oxyl/oxo intermediate, S_4abca_(R)-O· [4444] in Eq. (10a) under no participations of the Ca ion and His332 (Siegbahn 2006, 2009; Yamaguchi et al. 2010; Yamanaka et al. 2011; Greife et al. 2023).

10a $${\text{Tyr}} - {\text{O}} \bullet \, \left( {{\text{H}}^+ - {\text{His}}} \right) \, + {\text{ S}}_{{\text{3abba}}} \left( {\text{R}} \right) - {\text{OH }}\left[ {{4444}} \right] \to {\text{Tyr}} - {\text{OH}} + {\text{ S}}_{{\text{4abca}}} \left( {\text{R}} \right)\left( {{5444}} \right) = {\text{O }}\left( {{\text{S}}_{{\text{4abca}}} \left( {\text{R}} \right) - {\text{O}} \bullet \, \left[ {{4444}} \right]} \right)$$

10b $${\text{S}}_{{\text{4abca}}} \left( {\text{R}} \right)\left( {{5444}} \right) = {\text{O }} + {\text{ H}}_{2} {\text{O}}_{({7})} + {\text{Tyr}} - {\text{OH}} \to {\text{S}}_{{\text{4acca}}} \left( {\text{R}} \right) - {\text{Hydroperoxide }}\left( {{4443}} \right) \, + {\text{Tyr}} - {\text{OH}}$$

10c $${\text{S}}_{{\text{4abca}}} \left( {\text{R}} \right)\left( {{4444}} \right) - {\text{O}} \bullet \, + {\text{Tyr}} - {\text{OH}} \to {\text{S}}_{{\text{4abca}}} \left( {\text{R}} \right) - {\text{Peroxide }}\left( {{4443}} \right) \, + {\text{Tyr}} - {\text{OH}}$$

The mechanism (B) (Shoji et al. 2018a; Miyagawa et al. 2022a, b) becomes feasible if the one-electron transfer (OET) to Tyr-O radical is coupled with the O–O bond formation. For example, the intra cluster proton shift occurs to provide Mn-oxyl intermediate before the O–O bond formation as illustrated in Eq. (11a). S_3abca_(R)-Oxyl undergoes the O–O bond formation coupled with OET to Tyr-O radical, providing Mn-peroxide as shown in Eq. (11b). The potential curve crossing between the highest spin configuration of S_3abca_(R)-Oxyl (*S* = 6) and the intermediate spin state (*S* = 3) of S_4acca_(R)-Peroxide occurs at an intermediary configuration along the O–O bond formation pathway (Shoji et al. 2018a), indicating the non-adiabatic (NA) OET process involving the spin crossover for the O–O bond formation as shown in Eq. (11b). This process may be chemically regarded as Y_z_ participated O–O bond formation (Y_z_-OO) without the formation of Mn(V) site (see supporting information).

11a $${\text{Tyr}} - {\text{O}} \bullet \, \left( {{\text{H}}^+ - {\text{His}}} \right) \, + {\text{ S}}_{{\text{3abba}}} \left( {\text{R}} \right) - {\text{OH }}\left[ {{4444}} \right] \to {\text{Tyr}} - {\text{O}} \bullet \, \left( {{\text{H}}^+ - {\text{His}}} \right) \, + {\text{ S}}_{{\text{3abca}}} \left( {\text{R}} \right) - {\text{Oxyl }}\left[ {{4443}} \right]$$

11b $${\text{Tyr}} - {\text{O}} \bullet \, \left( {{\text{H}}^+ - {\text{His}}} \right) \, + {\text{ S}}_{{\text{3abca}}} \left( {\text{R}} \right) - {\text{Oxyl }}\left[ {{4443}} \right] \to {\text{Tyr}} - {\text{OH }}\left( {{\text{His}}} \right) \, + {\text{ S}}_{{\text{4abca}}} \left( {\text{R}} \right) - {\text{Peroxide }}\left( {{4443}} \right)$$

The parallel type B NA-OET (Shoji et al. 2018a) is compatible with the observed results by TR XFEL SFX experiment (Bhowmick et al. 2023). According to their experiment, the processes in Eq. (11a) and (11b) proceed during 500 (700) ~ 1200 μs because of the decrease of the electron density at the O_(6)X_ site, and the molecular oxygen release from the O_(5)_ site with second water insertion (H_2_O_(7)_) (Shoji et al. 2018b) starts after 1200 μs followed by release of second proton and molecular oxygen. The TR SFX XFEL experiments (Bhowmick et al. 2023) have revealed that the electron density of the O_(5)_ site decreases significantly at 1200 μs, which is accord with the oxygen release process. However, activation barriers for O_2_ release are highly dependent on samples under investigation, indicating 2 ~ 4 ms in some cases (Greife et al. 2023). Thus, the NA-OET is one of plausible processes compatible with the TR XFEL SFX experiment.

The Y_Z_-participated type B process is also feasible for the attack of the O_(6)_H anion to O_(5)_ = Mn(IV)_4_ coupled with OET to Tyr-O radical, affording S_3abca_(R)-O_(5)_O_(6)_H (Y_z_-OOH) with the (4443) valence state (see supporting information). The formation of the Mn(V) site is not necessary for the Y_Z_-assisted Mn-OOH production which is accord with the XES result (Davis et al. 2018). Successive deprotonation from the Mn-OOH occurs to provide Mn-peroxide (or superoxide) intermediate as shown in Eq. (12b). However, the proton transfer (PT) coupled with OET processes in Eq. (12a) and (12b) (Shoji et al. 2018b) proceed during 500 (700) ~ 1200 μs followed by the later release of molecular oxygen by the insertion of H_2_O_(7)_ as shown in Eq. (12c), which may be compatible with TR XFEL SFX (Bhowmick et al. 2023).

**Fig. 20:**
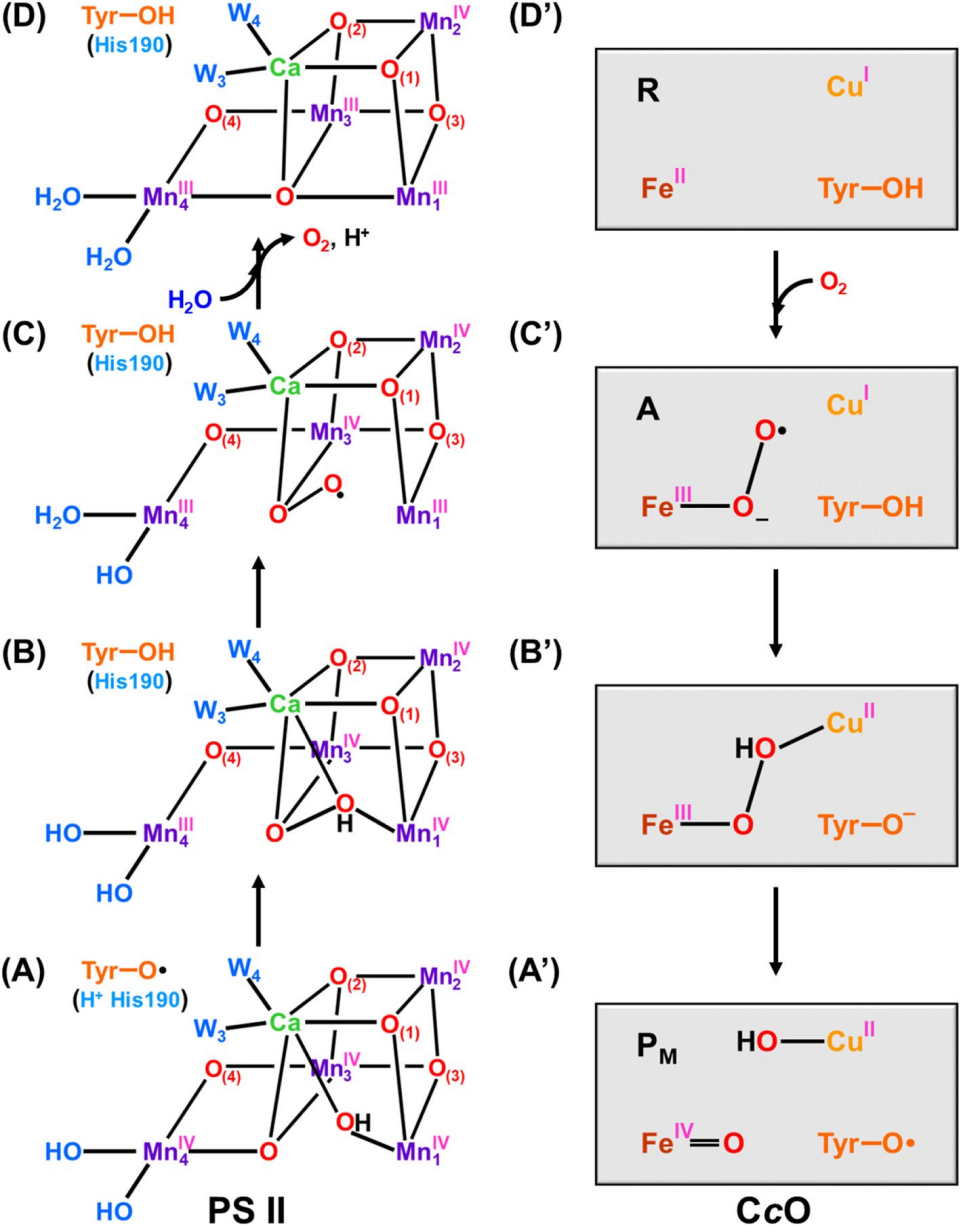
Reverse relationship between water oxidation by PSII and oxygen reduction by C*c*O; (**A**, **A**’). The P_M_ state of C*c*O corresponds to the S_3_-Tyr-O radical state of PSII, (**B**, **B**’). The O-OH bond formed state, (**C**, **C**’). Superoxide formed state and (**D**, **D**’). Final state (S_0_) of PSII and reactant state of C*c*O (see details in SVI)

The processes in Eq. (12) are formally regarded as the reverse process of oxygen reduction by cytochrome *c* oxidase (RC*c*O) as shown in Fig. 20 (see supporting information, SI Part VIII). Tyr244 is the fourth electron donor for oxygen reduction in the case of C*c*O, whereas Tyr161 is the fourth electron acceptor for oxygen evolution in OEC of PSII. The left configuration in Eq. (12a), namely S_3_-Tyr161-O radical, corresponds to the so-called P_M_ state of C*c*O (Yoshikawa and Shimada 2015; Wikström et al. 2018). The types A and B mechanisms in our classification do not require formation of Mn(V) (Fe(V)) site in a sharp contrast to the type C mechanism (Fe(V) in C*c*O).

12a $${\text{Tyr}} - {\text{O}} \bullet \, \left( {{\text{H}}^+ - {\text{His}}} \right) \, + {\text{ S}}_{{\text{3abba}}} \left( {\text{R}} \right) - {\text{OH }}\left[ {{4444}} \right] \to {\text{Tyr}} - {\text{OH }}\left( {{\text{His}}} \right) \, + {\text{ S}}_{{\text{4abba}}} \left( {\text{R}} \right) - {\text{OOH }}\left[ {{4443}} \right]$$

12b $${\text{Tyr}} - {\text{OH }}\left( {{\text{His}}} \right) \, + {\text{ S}}_{{\text{4abba}}} \left( {\text{R}} \right) - {\text{OOH }}\left[ {{4443}} \right] \to {\text{Tyr}} - {\text{OH }}\left( {{\text{His}}} \right) \, + {\text{ S}}_{{\text{4abca}}} \left( {\text{R}} \right) - {\text{OO}} \bullet \, \left[ {{3443}} \right]$$

12c $${\text{Tyr}} - {\text{OH }} + {\text{ S}}_{{\text{4abca}}} \left( {\text{R}} \right) - {\text{OO}} \bullet \, \left[ {{3443}} \right] \, + {\text{ H}}_{2} {\text{O}}_{({7})} \to {\text{Tyr}} - {\text{OH }} + {\text{ S}}_{0{\text{acca}}} \left( {\text{R}} \right) \, \left[ {{3433}} \right] \, + {\text{ O}}_{2} + {\text{ H}}^+_{{\text{lumen}}}$$

Thus, interplay between theory and experiments in Fig. 2 provides crucial information for understanding and explanation of plausible mechanisms of the O–O bond formation coupled with OET to Tyr161-O radical, which are compatible with the TR XFEL SFX results (Bhowmick et al. 2023). Recently, a seminal review article has been published to summarize present state of photosynthesis research (Shevela et al. 2023). Processes expressed by eqs. (9)-(12) are schematically illustrated in the supporting Figs. S7–S11. Re-examinations of potential energy diagrams for these processes by DLPNO CCSD(T_0_) are also under investigation.

### Concluding remarks

In this review, historical developments of the broken-symmetry (BS) methods for strongly correlated electron systems (Yamaguchi 1990a; Yamaguchi et al. 1986, 2022a) are revisited to explain prepared concepts and methods for theoretical investigation of water oxidation (see Fig. 2). Past decade, we have attempted to elucidate structure and bonding of whole intermediates in the S_i_ (*i* = 0 ~ 3) states of OEC of PSII by the interplay between theory and experiments (Yamaguchi et al. 2022a). Full geometry optimizations of the CaMn_4_O_x_ clusters (**1**) by the UB3LYP-D3/CPCM and QM(UB3LYP)/MM methods starting from HR XRD (Umena et al. 2011) have provided reasonable geometrical structures of the parent and proton-shifted isomers in each S_i_ step of the Kok cycle as compared with available geometrical parameters of Mn oxide model complexes (Mukhopadhyay et al. 2004) and EXAFS results (Yachandra et al. 1996; Yano and Yachandra 2014). The optimized geometries have provided several candidate structures for assignment of the most plausible structures for subtle different experimental structures even in the same S_i_ state by HR XRD and SFX XFEL. Therefore, UB3LYP-D3 level of theory has been applicable for geometry optimizations of possible intermediates structures of **1** on the theoretical grounds.

The large-scale QM(380 atoms)/MM computations (Shoji et al. 2015a, b) starting from HR XRD (Umena et al. 2011) have also refined hydrogen bonding networks around **1**, elucidating possible water inlet pathway (WIP) and proton release pathways (PRP) I, II, and III. The refined biomolecular system structures by the QM/MM are consistent with those of the low-dose (0.3 MGy) XRD structure (A momomer) (Tanaka et al. 2017). Thus, large-scale QM/MM computations have been effective for elucidation of reasonable static hydrogen bonding networks around **1** in the dark stable S_1_ state. QM/MM/MD computations are effective for elucidation of several dynamical processes such as extra water insertion and proton releases via conformational gateway in PRP I in the S_3_ to S_4_ transition (Greife et al. 2023).

DLPNO CCSD(T_0_) with def2-TZVP basis computations (Saitow et al. 2017) have been performed to elucidate relative stabilities among possible intermediates in each S_i_ state assuming the optimized geometries by UB3LYP-D3 method (Miyagawa et al. 2019, 2020, 2021, 2022a, b). For comparison, relative energies were also obtained by UB3LYP/def2-TZVP with different weights (10 ~ 20%) of the HF exchange term, which was used as a first step approach for transition metal oxides. Relative energies by DLPNO CCSD(T_0_)/def2-TZVP were found to be effective for elucidation of scope and reliability of the HDFT results for **1** in OEC of PSII. Particularly, DLPNO-CCSD(T_0_) was useful for elucidation of relative stability between the key S_3_ intermediates. DLPNO CCSD(T_0_) using ULO trials (Yamaguchi 1979) by UB3LYP is practical and handy as a refined step of beyond DFT approach for Mn oxides.

We have classified the O–O bond formation into three cases (Miyagawa et al. 2022a, b); (A) O–O bond formation before Tyr161-O radical reduction, (B) O–O bond formation coupled with Tyr161-O radical reduction, and (C) O–O bond formation after Tyr161-O radical reduction. Very recently, the TR XFEL SFX experiments (Bhowmick et al. 2023) have elucidated that the O–O bond formation and reduction of Tyr-O radical occur in parallel manner, supporting the mechanism (B). Our theoretical computations have elucidated two possible type B mechanisms: non-adiabatic (NA) one-electron transfer (OET) mechanism for the O–O bond formation (NA-OET) (Shoji et al. 2018a) and Y_Z_-participated O–OH bond formation, namely Y_Z_-OOH. The latter Y_Z_-OOH formation is formally regarded as a reverse process of oxygen reduction by cytochrome *c* oxidase (RC*c*O) as shown in Fig. 20. Further theoretical investigations based on the realistic models (> 227 atoms) by DLPNO CCSD(T_0_) remain for refinements of the mechanisms in relation to the TR XFEL SFX (Bhowmick et al. 2023) and TR FTIR (Greife et al. 2023).

## Supplementary Information

Below is the link to the electronic supplementary material.

Supplementary file1 (PDF 1998 kb)

## Abbreviations

AF: Antiferromagnetic
BB: Backbone
BS: Broken symmetry
CC: Coupled cluster
C*c*O: Cytochrome *c* oxidase
CCSD(T): Coupled cluster single double perturbative triple
CT: Charge transfer
DFT: Density functional theory
DLPNO: Domain based localized pair natural orbital
EC: Eigen complex
EPR: Electron paramagnetic resonance
ET: Electron transfer
ETM: Extended trimer model
EXAFS: Extended X-ray absorption fine structure
FR: Ferromagnetic
FTIR: Fourier transform infrared
GSO: General spin orbitals
HDFT: Hybrid density functional theory
HOMO: Highest occupied molecular orbital
HR: High resolution
HS: Highest spin
ICCMSE: International conference computational method in science and engineering
LUMO: Lowest unoccupied molecular orbital
JT: Jahn-Teller
MM: Molecular mechanics
NA: Non-adiabatic
OEC: Oxygen evolving complex
OET: One-electron transfer
PCET: Proton-coupled electron transfer
PNO: Pair natural orbital
PRP: Proton release pathway
PSII: Photosystem II
PT: Proton transfer
QM: Quantum mechanics
RHF: Restricted Hartree–Fock
RC*c*O: Reverse process of oxygen reduction by cytochrome *c* oxidase
SCES: Strongly correlated electron system
SFX: Serial femtosecond X-ray crystallography
SOMO: Singly occupied molecular orbital
SSB: Structural symmetry breaking
TR: Time-resolved
TZVP: Triple-zeta valence with polarization
UHF: Unrestricted Hartree–Fock
ULO: Localized natural orbitals
UNO: Natural orbital
XFEL: X-ray free electron laser
XRD: X-ray diffraction
WIP: Water inlet pathway
ZPE: Zero-point energy
