# Supplementary material for: Theoretical elucidation of the structure, bonding, and reactivity of the CaMn4Ox clusters in the whole Kok cycle for water oxidation embedded in the oxygen evolving center of photosystem II. New molecular and quantum insights into the mechanism of the O–O bond formation
Source: Photosynth Res. 2023 Nov 9;162(2-3):291–330. doi: 10.1007/s11120-023-01053-7 (PMC11614991; doi:10.1007/s11120-023-01053-7)
Supplement: Supplementary file 1 — Supplementary file1 (PDF 1998 kb) [file 11120_2023_1053_MOESM1_ESM.pdf]

Supporting Information (SI)

Theoretical elucidation of the structure, bonding, and reactivity of the $CaMn_4O_x$ clusters in the whole Kok cycle for water oxidation embedded in the oxygen evolving center of photosystem II. New molecular and quantum insights into the mechanism of the O-O bond formation

SI Part I Broken symmetry (BS) and beyond BS methods for open-shell systems

Broken symmetry (BS) and beyond BS (BBS) computations of strongly correlated electron systems

This review article summarizes fundamental principles and concepts for our theoretical investigations, understanding and explanation of oxygenic photosynthesis in eq. (1) in the main text. However, theoretical methods employed and computations performed are not descried in details in the main text. Therefore, supporting information (SI) on them are given to explain theoretical backgrounds of this review. Over past decades, we have been involved in quantum chemistry of strongly correlated electron systems (SCES), where the electron-electron repulsion effects are essential for elucidation of the nature of chemical bonds in a sharp contrast to the extended Hückel MO (EHMO) model. From our theoretical view, $CaMn_4O_5$ (**1**) in OEC of PSII is a typical example of SCES, for which four degrees of freedom are very important as illustrated in Fig. 2 in the main text. This means that more general molecular orbital models than EHMO are necessary for SCES. Figure S1 illustrates our basic MO-theoretical approaches to SCES: (1) Instability analysis, (2) constructions of generalized Hartree Fock (GHF) solutions responsible for types of the instability conditions at the BS level of QM computation, (3) natural orbital analysis of the GHF solutions to elucidate the natural orbitals (UNO) and their occupations numbers (*n*), and (4) single (SR) and multi-reference (MR) configuration interaction (CI) and/or coupled cluster (CC) computations as beyond BS level of QM computations (Yamaguchi 1980; Yamaguchi et al. 2022a).

The steps (1) and (2) are usually regarded as a mean-field theory in material science. For example, the restricted Hartree-Fock (RHF) solution is applicable to closed-shell molecules with stable chemical bonds. Scope and applicability of the RHF solution is usually checked by several instability conditions (Stability check in Gaussian Program), often indicating the triplet-type instability which entails the more stable unrestricted Hartree-Fock (UHF) solutions consisted of the different orbitals for different spins (DODS) configuration for diradical and polyradical molecules. On the other hand, the singlet-type instability of the RHF solution provides the charge density wave (CDW) solutions for polar bonds. The Haree-Fock-Bogoliubov (HFB) solution is responsible for the superconductivity. Thus, the instability

conditions are useful for understanding "What kind of the electron correlations is crucial for systems under examination among possible general Haree-Fock (GHF) solutions" as shown in Fig. S1. The above instability conditions are also applicable to the Kohn-Sham (KS) type density functional theory (DFT), providing the bifurcations of the KS DFT orbitals, for example unrestricted DFT (UDFT). Therefore, the instability condition is also applicable to the hybrid UHF plus UDFT models such as the UB3LYP model used in this review.

The step (3) provides the natural molecular orbitals (UNO) and their occupation numbers (n_i) which are used for constructions of single (SR) and multi-determinant (MR) references of the step (4), namely beyond mean-field approaches such as configuration interaction (CI) and coupled-cluster (CC) methods as illustrated in Fig. S1. In this review, SR CC single (S), double (D), and perturbative triple (T) approximation is used as one of the GHF CC approaches using the UNO trials MOs in Fig. S1. The UNO are obtained by the diagonalization of the first order density matrix of the hybrid DFT (HDFT) solutions. UNO are often transformed into the localized UNO (ULO) for selections of active ULOs. For example, UNO are reduced to the domain-based localized pair natural orbitals (DLPNO) in this review (see SI Part VI).

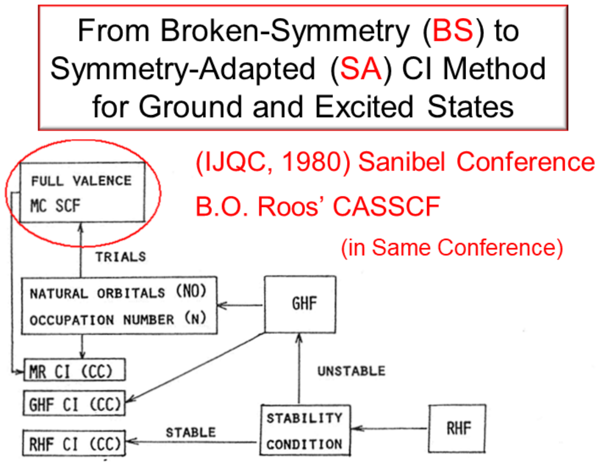

Fig. S1 Computational schemes for strongly correlated electron systems (SCES) (Yamaguchi 1975a, 1980). As a first step, broken symmetry (BS) computations (unrestricted Hartree Fock (UHF), generalized Hartree Fock (GHF), etc.) are performed to elucidate the ground electronic and spin structures of SCES. Beyond BS calculations such as multi-reference (MR) configuration interaction (CI), MR coupled cluster (CC) using the natural orbitals (UNO) or localized natural orbitals (ULO) of the BS solutions are performed for both ground and excited states of SCES. Scope and reliability of the BS computational results are examined by beyond BS computations in Fig. S1. The author (KY) is grateful to Professors R.J. Buenker and S.D. Peyerimhoff for their kind discussions on our approaches at that time (1980).

Instability, HOMO-LUMO mixing and diradical configuration (BS) : Ozone

In this section, the molecular orbital (MO) model is extended to diradical species such as ozone (Yamaguchi 1975a) instead of the valence bond (VB) model. First of all, ab-initio MO model is examined instead of the EHMO model. Stable closed-shell molecules with the large HOMO-LUMO gap are well described by the restricted Hartree-Fock (RHF) MO model as shown in Fig. 1 of the main text. The RHF and RHF based coupled cluster (RCC) models are useful enough for theoretical investigations of structure and bonding of closed-shell metal oxides as shown in the review article by Hoffmann (1982) and Holm (1987). On the other hand, the HOMO-LUMO gap becomes small for unstable molecules with nearly degenerated RHF orbitals, suffering the instability condition, which entails the more stable UHF solutions via the HOMO-LUMO mixing (Yamaguchi 1975a) as illustrated in Fig. 1 and Fig. S1. The resulting MOs by the mixing are spatially symmetry broken since HOMO and LUMO are usually different spatial symmetries. Thus, the concept of the orbital symmetry breaking is emerged for diradical reactions in sharp contrast to the orbital symmetry conservation for concerted reactions (Hoffmann 1982).

One of such examples is a harmful process in biology; namely the dissociation process of the O-O bond; for example, HO-OH into 2 OH radicals through diradical state [HO•…•OH] (Yamaguchi et al. 1992). The reverse process is well known the radical coupling (RC) process (Siegbahn 2006). Ozone may be another example for diradical state. The closed-shell singlet RHF solution (O=O=O) indeed is unstable than the UHF solution with the triplet spin state (↑•O-O-O•↑). In this situation, the singlet RHF solution exhibits the triplet instability, which entails its reorganization into the singlet UHF solution via the HOMO-LUMO mixing as shown in Fig. S1 (Yamaguchi 1975a). The resulting UHF molecular orbitals are localized mainly on the left and right oxygen atoms, respectively, providing different orbitals for different spins (DODS). The electrons with the up- and down-spins enter into the DODSs, providing the singlet diradical configuration (↑•O-O-O•↓), where up and down spins are expressed by allows ↑ and ↓, respectively. The singlet diradical configuration (↑•O-O-O•↓) becomes more stable than the triplet diradical configuration (↑•O-O-O•↑). The DODS models are applicable to the dissociation and/or recombination (radical coupling (RC)) reactions; [↑•O-O-O•↓] ↔ [↑•O-O•↑ + ↓•O•↓], which are also examples of the open-shell reactions in the ozone layer of the stratosphere. Thus, radical reactions can be described with an extension of the MO concept, the concept of the DODS, in Fig. S1.

Isolobal and isospin analogy between organic and inorganic radical species

A next step is how to understand chemical bonding of organic and inorganic open-shell molecules in a unified manner within the MO model. Hoffmann (1982) have presented the isolobal analogy between organic and inorganic molecules for qualitative understanding on the basis of the EHMO model,

elucidating the eight (8) and eighteen (18) electron rules for chemical bonding of organic and inorganic compounds. For example, $(CO)_5Mn(0)$-$Mn(0)(CO)_5$ molecule is a closed-shell molecule belonging to the 18-electron rule. However, the dissociation of the Mn-Mn single bond provides the metal diradical state; $(CO)_5Mn(0)$ •…• $Mn(0)(CO)_5$. The chemical bonding of many stable organometallic compounds is usually explained by the 18-electron rule, but it often dissociates into a metal radical state.

Redox-active metal oxides are often exceptions of the 18-electron rule. In 1970s, Calvin (1974) has already emphasized an important role of manganese oxo double bond ($L_5Mn(X)$=O, X = III, IV) with octahedral ligand field (L) for water oxidation as mentioned in the main test. This type of Mn complexes with the octahedral ligand filed does not satisfy the 18-electron rule, indicating the open-shell ground states in metalloenzymes. Formal organic analog to the M=O double bond of the Mn-oxides may be regarded as the C=O double bond of carbonyl oxides R_1R_2C=O. Nevertheless, the elongation of the X-O (X = C, M) bond entails the HOMO-LUMO mixing, providing the radical states with DODSs. According to this analogy, both orbital and spin concepts are necessary for open-shell species obtained by the dissociation of the double bonds. Over past decades, the concept of isolobal and isospin analogy in the MO model has been useful for understanding and explanation of radical reactions by both organic and inorganic radical species. As an example, RHF orbital instability is further examined in the dissociations of double bonds of organic (C=O)- and high-valent metal (M)-oxygen (M=O) compounds as described follows.

The singlet C=O double bond dissociates into triplet two fragments

In this section, the dissociation of the singlet double bond into two triplet diradicals is examined on the basis of the MO model. Bond lengths of C=O bonds of stable carbonyl compounds are around 1.23 Å, indicating no oxyl-radical character. However, the HOMO-LUMO gap of the π-type RHF orbitals becomes small at the elongated C-O bond, indicating the HOMO-LUMO mixing which provides the π-type BS orbitals localized mainly on the C and O sites, respectively, which accords with the organic π-diradical structure •H_2C-O• where • denotes the localized electron (spin) (Yamaguchi 1975a; Yamaguchi et al. 2021). The further bond-elongation entails the σ-type HOMO-LUMO mixing of •H_2C-O• provides the σ-type BS orbitals localized on the C and O sites, respectively, which accord with the organic tetra-radical structure ••H_2C…O••.

$$H_2C{=}O \quad \rightarrow \quad \uparrow\bullet H_2C{-}O\bullet\downarrow \quad \rightarrow \quad \uparrow\uparrow\bullet\bullet H_2C\ldots\ldots O\bullet\bullet\downarrow\downarrow \qquad \text{(s1a)}$$

Two triplet fragments are obtained at the dissociation limit of the singlet C=O double bond. Therefore,

this type of the dissociation reaction cannot be described by the coupling of two singlet pairs as shown in the perfect pairing (PP) generalized valence bond (GVB-PP) method. This is the reason why the broken-symmetry (BS) MO models are practically useful for locations of oxygenation reactions, for which triplet atomic oxygen and molecular oxygen play important roles as shown in the main text (Yamaguchi 1990a).

Oxyl radical character of M=O and MOO via the HOMO-LUMO mixing

In this section, the oxyl-radical character of the high-valent metal oxides is examined at the BS MO level. In 1980s, BS methods have been applied to MO descriptions of electronic and spin structures of high-valent metal-oxo (M=O) bonds as shown in Fig. 1 of the main text (Yamaguchi et al. 1986, 2021). The HOMO-LUMO gap of the high-valent M=O (M = 4d or 5d metals such as Ir, W) is large, indicating no instability; no oxyl-radical character in Fig. 1. On the other hand, the HOMO-LUMO gap of the high-valent M=O (M = 3d metal) often becomes small, indicating the HOMO-LUMO mixing which entails the π-type BS orbitals (•M-O•) as shown in Fig. 1 in the main text. The further elongation entails the σ-type HOMO-LUMO mixing of •M-O• provides the σ-type BS orbitals localized on the M and O sites, respectively, which accords with the tetra-radical structure ••M…O••. The oxygen atom O•• in these tetra-radical species has two local spins with radical reactivity as shown in eq. (s2). Therefore, triplet oxygen (^{3}O) model is often applicable to understand radical reactivity of oxygen site of the high valent M=O bonds with oxyl-radical character (•M…O•, ••M…O••) (Yamaguchi 1985; Yamaguchi et al. 1986).

$$\mathrm{M(X)=O} \;\rightarrow\; \uparrow\bullet\mathrm{M(X-1)\text{-}O}\bullet\downarrow \;\rightarrow\; \uparrow\uparrow\bullet\bullet\mathrm{M(X-2)....O}\bullet\bullet\downarrow\downarrow \qquad \text{(s2a)}$$

Atomic oxygen (O), molecular oxygen (O=O) and Fe(IV)-oxo (Fe=O) bond are the ground triplet species. The elongations of the O-O and Fe-O bonds entail localizations of radical orbitals, providing radical reactivity like atomic oxygen (Yamaguchi 1985). Therefore, these species are often regarded as isolobal and isospin from $S = 1$ state, exhibiting the oxyl-radical character of the oxygen site.

Similarly, the isospin analogy is feasible for organic 1,3-diradical and 1, 3-metal-diradical species: (a)-(d). The metal-peroxide radical often exhibit the radical reactivity (Yamaguchi 1983).

(a) ↑•O-O-O•↓ and ↑•Cu(II)-O-Cu(II)•↓,

(b) ↑•C-O-O•↓ and ↑•Cu(II)-O-O•↓,

(c) ↑•C-O-O•↓ and ↑•Fe(III)-O-O•↓.

(d) ↑•C-O-O•↓ and ↑↑↑↑•Mn(III)-O-O•↓.

Complex reactivity of carbonyl oxides R_1R_2C-O-O is indeed suggestive for understanding of chameleonic reactivity of L_1L_2Fe(III)-O-O compounds and related metal peroxides L_1L_2M-O-O, indicating the utility of the isolobal and isospin analogy (Yamaguchi 1983; Yamaguchi et al. 1988b, 1992). This in turn indicates important roles of ligands L_1 and L_2 for active controls of their reactivity.

Thus, BS MO methods have elucidated the isolobal and isospin analogy between oxygen radical sites of organic and inorganic oxides, providing guiding principles for understanding and explanation of chemical reactions by active oxygen and oxy-radicals on the common theoretical ground. The isolobal and isospin analogy is useful enough for systematic explanations of oxygen radical reactions such as mono-oxygenations by P450 involving Fe(IV)=O and radical coupling (RC) reactions of the O-O bond formation by Mn=O (Yamaguchi et al. 2010) as shown in eqs. (s3a and b). The isolobal and isospin analogy has been extensively described in our recent review article (Yamaguchi et al. 2021).

Local singlet and local triplet diradical configurations and spin correlation diagrams

In this section, the spin coupling for the O-O bond formation is revisited (Yamaguchi 1983). The radical coupling (RC) mechanism between the oxyl-radicals becomes popular after the proposal by Siegbahn (2006) for the O-O bond formation for water oxidation. The oxyl-radical character is responsible for many radical reactions such as the mono-oxygenations of alkenes and alkanes mentioned above. The RC reaction becomes feasible for the O-O bond formation as illustrated in eq. (s3) (see later). To this end, the local singlet diradical (LSD) configuration [O•↓…↑•O] is necessary for facile O-O bond formation as shown in eq. (s3a). On the other hand, the spin inversion (SI) is necessary if the local triplet diradical (LTD) configuration is formed during radical reactions as illustrated in eq. (s3b) (Yamaguchi et al. 2010, 2012).

↑•M(X–1)-O•↓ + ↑•O-M(X–1)•↓ → ↑•M(X–1)-O-O-M(X–1)-O•↓ (s3a)

↑•M(X–1)-O•↓ + ↓•O-M(X–1)•↑ → SI → ↑•M(X–1)-O-O-M(X–1)-O•↓ (s3b)

The fundamental problem is to elucidate formation of LSD and LTD configurations in the case of multi-center transition-metal clusters such as **1** for water oxidation. To this end, elucidation of spin structures of these clusters is crucial as shown in our papers (Yamaguchi et al. 2010, 2021). Indeed, BS methods provide spin correlation diagrams for theoretical investigations of RC mechanisms catalyzed by multi-nuclear complexes such as **1**. We have depicted spin correlation diagrams for elucidation of formations of LSD or LTD configurations to derive selection rules for possible RC reaction pathways by key Mn model clusters (Brudvig and Crabtree 1986; Yachandra et al. 1996; Ferreira et al. 2004; Yano et al.

2005a; Dismukes et al. 2009) (Yamaguchi et al. 2010) before the discovery of the HR XRD structure (Umena et al. 2011). Therefore, after the discovery, we have examined the spin structures of **1** revealed by HR XRD (Kanda et al. 2011b; Yamanaka 2011), proposing possible RC pathways for the high-valent HR XRD structure (Umena et al. 2011).

SI Part II Derivations of chemical indices for the high-valent metal oxo bonds

Triplet instability, HOMO-LUMO mixing and Orbital bifurcation

In this section, the orbital bifurcation via the HOMO-LUMO mixing is explained on the theoretical ground. To this end, several theoretical formulations have been derived for elucidation of the nature of the high-valent metal-oxo (M=O) bonds (Yamaguchi et al. 1986; Larson et al. 2020). The energy gap between the dπ-pπ bonding (HOMO) and dπ-pπ* anti-bonding (LUMO) orbitals is usually large for high-valent 4d (5d) transition metal-oxo species (Holm 1987; Meunier et al. 2004; Kärkäs et al. 2014). The dπ-pπ bond of M(V)=O is regarded as a closed-shell bond without the oxyl-radical character as shown in Fig. 1, providing the AB-type two electron (V-III; IV-II) process for the O-O bond formation in the text. However, the HOMO-LUMO energy gap given by $\Delta\varepsilon = \varepsilon_{\mathrm{LUMO}} - \varepsilon_{\mathrm{HOMO}}$ is often smaller than the on-site electron repulsion integral (U) in the case of high-valent 3d M=O bonds, indicating the instability condition as shown in eq. (s4) (Yamaguchi 1975a; Döhnert and Koutecký 1980).

$$\Delta\varepsilon \,/\, U = \varepsilon_{\mathrm{LUMO}} - \varepsilon_{\mathrm{HOMO}} \,/\, U < 1 \qquad \text{(s4)}$$

where the ε_X (X = HOMO, LUMO) denotes the orbital energy of X. The instability condition indicates that the closed-shell bond is reorganized into the more stable spin-polarized (SP) bond given by the HOMO-LUMO mixing procedure (Yamaguchi 1975a) of the unrestricted (U) Hartree Fock (HF), Kohn-Sham (UKS) DFT, and hybrid DFT (UHDFT) approximations based on the single Slater determinant model (Slater 1972). Figure 1 in the main text illustrates the orbital bifurcation responsible for the oxyl-radical character (Yamaguchi et al. 1986, 1990a). The resulting MOs at the UHF (UHFS, UHDFT) level of theory are given by the HOMO-LUMO mixing by the restricted (R) versions of these solutions (Yamaguchi 1975a):

$$\psi_i^{+} = \cos\theta\,\phi_i + \sin\theta\,\phi_i^{*} \qquad \text{(s5a)}$$

$$\psi_i^{-} = \cos\theta\,\phi_i - \sin\theta\,\phi_i^{*} \qquad \text{(s5b)}$$

where θ denotes the orbital mixing-parameter determined by the above methods. Since HOMO, ϕ_i and LUMO, ϕ_i^*, by RHDFT are symmetry-adapted and usually belong to different spatial symmetries (P_n), UHDFT MOs, ψ_i^+ and ψ_i^-, obtained by the HOMO-LUMO mixing are often spatially symmetry-broken as shown in Fig. 1. This is the origin of the common naming of "broken symmetry (BS)" in the Hartree-Fock (HF), DFT, and hybrid DFT methods.

The BS orbitals, ψ_i^+ and ψ_i^- are mainly localized on the M- and O-sites, respectively, as illustrated in Fig. 1. Therefore, the latter orbital is similar to the singly occupied orbital of oxygen atom, OH radical, etc., indicating the oxy-radical character (Yamaguchi et al. 1986, 1990a). The dσ-pσ bonding (σ-HOMO) and dσ-pσ* anti-bonding (σ-LUMO) orbital mixing also occurs under the condition that the energy gap between σ-HOMO and σ-LUMO is small as shown in eq. (s4), providing the BS orbitals. The resulting BS orbital at the O-site indicates the σ-type oxyl-radical character in this case in accord with the naming of the BS method in general.

The merit of the BS method is its applicability to large molecular systems involving SCES in both quantum mechanics (QM) and QM/molecular mechanics (MM) computations. In fact, BS HDFT method has been used for the QM part of the QM/MM computations. The natural orbital (NO) analysis of the first order density matrix by the BS method provides the natural orbitals (UNO, ULO) and their occupation numbers as shown in Fig. S1. UNO (ULO) is utilized for the beyond BS methods such as MR CI (CC), which also provide several useful chemical indices. Thus, the chemical indices are regarded as the conceptual bridges between BS and beyond BS methods as described below (see Fig. S2).

Orbital overlap between BS orbitals and effective bond order

In this section, an effective bond order is first derived among several chemical indices, which have been employed to investigate the nature of chemical bonds of open-shell systems such as •M-O• expressed by eq. (s5). To this end, the orbital overlap T_i between BS MOs obtained by the HOMO-LUMO mixing in eq. (s5) is defined as (Yamaguchi 1975a, 1990a).

$$T_i = \langle \psi_i^+ | \psi_i^- \rangle = \cos 2\theta \qquad \text{(s6)}$$

Therefore, T_i becomes 1.0 in the case of the closed-shell (restricted) case; $\psi_i^+ = \psi_i^- = \phi_i$, whereas T_i is 0.0 for the complete mixing case ($\theta = \pi/4$): complete SP split pair. In order to express the decrease of chemical bonding via orbital symmetry breaking, the Coulson's (first order) effective bond order is extended to as follows (Yamaguchi 1975a)

$$b_i = \frac{n_i - n_i^*}{2} = \frac{(1+\cos 2\theta)-(1-\cos 2\theta)}{2} = \cos 2\theta = T_i \qquad \text{(s7)}$$

where n_i and n_i^* denote the occupation numbers of the bonding (HOMO) and antibonding (LUMO) orbitals, respectively, and they are expressed by using the orbital overlap; $n_i = 1 + T_i$ and $n_i^* = 1 - T_i$. The effective bond order (b) is nothing but the orbital overlap between BS MOs under the BS approximations.

The orbital overlap (T_i) between BS orbitals in eq. (s6) is variable with the weight of the Hartree-Fock exchange term (w) in the hybrid DFT (HDFT) models. Therefore, the w-values have been assumed to be variable in the 10 ~ 20 % range of w in our series of papers. The nature of the chemical bonds of the metal-oxo bonds are variable, depending on w. Therefore, relative energies among several intermediates in the S_i state are also variable with w (Isobe et al. 2016; Yamaguchi et al. 2018, Miyagawa et al. 2020), indicating a semi-theoretical nature of HDFT for SCES such as **1**. Thus, beyond HDFT computations have been inevitable to examine scope and reliability of HDFT results. To this end, DLPNO CCSD(T_0) computations have been performed to elucidate the relative energies (Miyagawa et al. 2019, 2020, 2021, 2022a, b). The comparison between HDFT and DLPNO CCSD(T_0) results for reference molecules in turn provides a reasonable w value of HDFT for related large molecular systems under examination.

Quantum resonance between BS solutions and effective exchange integrals

In this section, the concept of the quantum resonance is introduced for recovery of the broken symmetry. The concept of the orbital symmetry breaking is useful for qualitative understanding and explanation of the oxyl-radical character of the high-valent metal-oxo bonds at the MO level. However, this does not mean the true symmetry breaking in the phase transition of the solid-state physics (Anderson 1973). The Slater determinant consisted of BS MOs, namely a mean field model, is not an eigen function of the total spin operator $<\boldsymbol{S}^2>$ required from quantum mechanics (QM) of finite systems. However, two BS solutions, namely the up-down ↑•Mn(IV)-O•↓ and down-up ↓•Mn(IV)-O•↑ BS solutions, are emerged and degenerated in energy, indicating the same antiferromagnetic spin correlation in finite systems.

Therefore, the quantum resonance (Anderson 1973) between these BS configurations is inevitable under the finite overlap interaction, indicating the recovery of the broken-symmetry state. The resonating BS (RBS(+)) solution is indeed obtained by the in-phase combinations of these BS solutions, providing the pure singlet state satisfying the $<\boldsymbol{S}^2>$ operator because of the elimination of the triplet component involved in each single Slater-determinant BS configuration. On the other hand, the out of phase combination of two BS configurations, namely RBS(–) solution, provides the pure triplet state satisfying the $<\boldsymbol{S}^2>$ (= 1(1+1) = 2) operator. The energy gap between the singlet and triplet state is formally expressed by the effective exchange integrals (J) in the quantum Heisenberg model

$$^1E(RBS(+)) - {}^3E(RBS(-)) = 2J. \qquad (s8)$$

The J value plays an important role for analysis of the EPR results.

Weight of the doubly excited configuration and oxyl-radical characters

In this section, the diradical character is introduced as a common index obtained by the symmetry-recovered RBS (RBS(+)) and configuration interaction (CI) solutions. To this end, RBS(+) is rewritten by the 2 × 2 CI wavefunction constructed by the natural orbitals (UNO) of the BS solutions as shown in Fig. S1 (Yamaguchi 1975a, 1990b)

$$\Phi_{RBS(+)} = \frac{1}{\sqrt{2(1+T_i^2)}}\{(1+\cos 2\theta_i)(\phi_{HOMO-i}\overline{\phi_{HOMO-i}}) - (1-\cos 2\theta_i)(\phi^*_{LUMO+i}\overline{\phi^*_{LUMO+i}})\} \qquad (s9)$$

where the first and second terms denote the ground and doubly excited configurations, respectively. This UNO(MO)-CI picture (Yamaguchi 1975a, 1980; Döhnert and Koutecký 1980) is useful for theoretical understanding of labile chemical bonds with moderate orbital overlaps in eq. (s6). For example, manganese oxo (Mn=O) (Yamaguchi et al. 1986) and peroxo (Mn-O-O) (Yamaguchi 1983; Yamaguchi et al. 1988b, 1992) bonds in high-valent manganese oxides exhibit labile behaviors because of variable θ_i values. The effective bond order (B) is defined by $\Phi_{RBS(+)}$ to express the nature of these labile chemical bonds as

$$B_i = \frac{n_{HOMO-i}(RBS(+)) - n_{LUMO+i}(RBS(+))}{2} = \frac{2b_i}{1+b_i^2} \geq b_i \qquad (s10)$$

The effective bond order (B_i) after elimination of triplet contamination in the BS solution is larger than that (b_i) of the BS solution itself. This is not at all trivial, indicating that BS computational results without symmetry recovery are often biased to radical picture arising from the high-spin component. In fact, the spin density increases sharply with the decrease of the orbital overlap (T_i) as shown in Fig. S2.

The oxyl radical character (Y) after elimination of spin contamination (Yamaguchi 1975a) is defined by the weight of the doubly excited configuration under the UNO (MO) CI approximation in eq. (s9) (Yamaguchi 1975a; Döhnert and Koutecký 1980) as

$$Y = 2W_D = \frac{(1-T_i)^2}{1+T_i^2} = 1 - \frac{2T_i}{1+T_i^2} = 1 - B_i \qquad (s11)$$

The oxyl radical character Y (= y) is directly related to the decrease of the effective bond order B after spin projection. These chemical indices are used for diagnosis of radical reactivity of manganese oxide species such as $CaMn_4O_x$ (x = 5, 6) clusters (Isobe at al. 2012; Yamanaka et al. 2012).

The BS (RBS(+)) MO solution is rewritten by localized UNO (ULO) CI to obtain the zwitterionic (ZW) and covalent (CO) configurations (Yamaguchi 1990a), namely variable ionic and radical characters: this is an origin of chameleonic (radical/electrophilic) reactivity of these bonds. Thus, ULO is useful for the resonating VB (RVB) theoretical explanations for open-shell species. The ULO are useful for constructions of trial orbitals for beyond BS computational procedures such as MkMRCC and UCCSD(T) based on the domain-based local pair natural orbital (DLPNO).

Spin density, unpaired electron density and pair and spin correlation functions

In this section, possible roles of the spin density by the BS model are examined on the theoretical ground. Spin density by the BS method is a useful chemical index for understanding radical reactivity of local spins (•). However, spin densities disappear in the case of the pure singlet state arising from the quantum resonance $\Phi_{RBS(+)}$. In 1970s, spin density in the singlet UHF solution was therefore an important origin against the BS approach to radical reactions (Yamaguchi 1975a). Thus, an approximate spin projection (AP) scheme was developed based on the quantum Heisenberg model so as to eliminate spin contamination in the BS solution (Yamaguchi et al. 1986; Yamaguchi 1990a). As a result, our computational scheme of the J values is in turn applicable to BS, AP BS, CASSCF, and MR CI (CC) methods (Yamaguchi et al. 1986, Yamaguchi 1990a)

However, even at that time, we noticed the temperature dependent para-magnetism observed for the singlet state of the exchange coupled transition-metal oxides such as the Cu(II)-O-Cu(II) bonds (Yamaguchi et al. 1986) and Cr(II)-Cr(II) metal-metal bonds (Yamaguchi 1979), indicating thermal mixing of high-spin component into the low-spin ground state. The neutron diffraction results were also used to elucidate the spin correlation functions of antiferromagnetic solids consisted of the Cu(II)-O-Cu(II) unit (Bednorz and Müller 1986; Yamaguchi 1990b). However, short-range magnetic order in doped copper oxide is destroyed by quantum fluctuations (Bednorz and Müller 1986). Therefore, we have considered electron correlation and spin correlation functions to elucidate possible roles of spin density in open-shell species under the BS approximation (Yamaguchi 1990a).

To this end, the second-order pair and spin correlation functions of the BS solutions were indeed useful to elucidate important roles of spin densities for exchange-coupled systems (Yamaguchi 1990a). The on-site pair function (P_2) for electrons with different spins is given by

$$P_2(\boldsymbol{r}_1, \boldsymbol{r}_1; \boldsymbol{r}_1, \boldsymbol{r}_1) = \frac{P_1(\boldsymbol{r}_1, \boldsymbol{r}_1)^2 - Q_1(\boldsymbol{r}_1, \boldsymbol{r}_1)^2}{2} \quad \text{(s12)}$$

where $P_1(\mathbf{r}_1, \mathbf{r}_1)^2$ and $Q_1(\mathbf{r}_1, \mathbf{r}_1)^2$ denotes, respectively, the square of density and spin density. This means that the magnitude of spin density is directly related to the size of Coulomb hole (mutual repulsion) for electrons with different spins, providing an important theoretical picture that size of the dot • in preceding section means the magnitude of Coulomb hole on the theoretical ground. Therefore, the eq. (s12) was used for DFT correlation function for the MR DFT approach. Moreover, the unpaired electron density $U(\mathbf{r}_1)$ responsible for deviation from the single determinant is expressed by the square of spin density under the BS approximation as (Yamaguchi 1990a)

$$U(\boldsymbol{r}_1) = Q^2(\boldsymbol{r}_1, \boldsymbol{r}_1) = Q^2(\boldsymbol{r}_1) = \sum n_i(2 - n_i). \quad \text{(s13)}$$

The magnitude of spin densities reported in various recent BS calculations can be understood from the viewpoint of non-dynamical correlations between electrons with different spins, namely, strong electron repulsion effects in SCES in Fig. 2 and Fig. S1. Therefore, the spin density index is also introduced to express the characteristic behavior of the spin density as

$$Q_i(\boldsymbol{r}_1, \boldsymbol{r}_1) = \sqrt{U_i(r)} = \sqrt{1 - T_i^2}, \; Q(\boldsymbol{r}_1, \boldsymbol{r}_1) = \sum_i Q_i(\boldsymbol{r}_1, \boldsymbol{r}_1). \quad \text{(s14)}$$

On the other hand, how about the sign of spin densities is a basic problem under the BS approximation. In order to resolve this problem, the spin correlation function is introduced since it can be observed in the case of infinite systems with neutron diffraction technique (Yamaguchi 1990a). In fact, the spin correlation function $K_2(\mathbf{r}_1, \mathbf{r}_2)$ for the BS solution is approximately given by

$$K_2(\mathbf{r}_1, \mathbf{r}_2) = \int \boldsymbol{s}(1)\boldsymbol{s}(2)P_2(\boldsymbol{r}_1, \boldsymbol{r}_2; \boldsymbol{r}_1, \boldsymbol{r}_2)\mathrm{d}s \approx Q(\boldsymbol{r}_1)Q(\boldsymbol{r}_2) \quad \text{(s15)}$$

where P_2 denotes the second-order density matrix. This means that the spin correlation is singlet-type if the sign of spin density product is negative in sign; (↑↓) or (↓↑), although such short-time spin order cannot be detected by the neutron diffraction because of the quantum resonance mentioned above (Anderson 1973). Therefore, the spin correlation function is a conceptual bridge between BS and beyond BS models such as CASSCF, UNO CI, etc. in Fig. S1.

Spin structures and spin correlation diagrams for multi-nuclear systems

In this section, spin structures for multi-nuclear systems are touched briefly. The spin correlation function in eq. (s15) provides a theoretical procedure of the spin-dependent expressions of SCES in Fig. 2 in the main text. Indeed, spin structures for multi-nuclear systems are regarded as a schematic illustration of spin correlation functions in eq. (s15). For example, BS methods provide eight spin structures of **1** (Kanda et al. 2011b) as shown in Fig. 7 in the main text. These spin structures are approximately regarded as a schematic expression of the spin correlation functions, which can be obtained by the second order density matrix (see in eq. (s15)) of the beyond BS methods such as the UNO MR CI (CC) in Fig. S1. Therefore, we have used a pictorial expression of the spin correlation functions for qualitative purpose, for example (↑↓↓↑), of the antiferromagnetic (AF) low-spin (LS) configuration of poly-radical species such as the $CaMn_4O_5$ cluster in OEC of PSII since the sign of spin density product is closely related to the spin correlation function under the BS approximation as shown in eq. (s15).

Although the spin densities arising from the first-order density $P_1(\mathbf{r}_1, \mathbf{r}_2)$ disappears at the pure singlet state, RBS(+), the unpaired electron density ($U(\mathbf{r}_1)$) and spin correlation function (K_2) still exist as important spin and electron correlation indices even in the resonating BS (RBS) and symmetry-adapted multi-reference (MR) beyond BS wave functions such as MR CI (CC) and MR DFT, complete active space (CAS) CI (Yamaguchi 1980), and CASSCF (Roos 1980). Therefore, sign and magnitude of spin densities in the BS approach should be understood from the above theoretical viewpoints in eqs. (s12)-(s15). The pair and spin correlation functions can be used to elucidate the nature of chemical bonds of SCES such as **1** in the case of beyond BS RBS and MR approaches as alternative indices for spin density at the BS UB3LYP level of theory.

Functional behaviors of several chemical indices with the orbital overlap

In this section the information entropy is introduced as a chemical index. As described above, several chemical indices are derived for understanding and explanation of the nature of chemical bonds of open-shell molecules and clusters. Figure 2 illustrates their functional behaviors against the orbital overlap between BS orbitals, namely the order parameter in the solid-state physics (Anderson 1973). Recently, information entropies have been accepted great interest in several fields. The information entropy is a useful measure of quantum effects in SCES. The information entropy (I_n) for chemical bonds is also defined by the occupation number (Shoji et al. 2004) as

$$I_i = -n_i \ln n_i,\ I_c = -2 \ln 2,\ I_n = I_i / I_c. \qquad \text{(s16)}$$

The ratio between I_i and I_c denotes the bond order defined by the information entropy. Its behavior is similar to that of the effective bond order (*b)* as shown in Fig. S2. Several chemical indices defined by the occupation numbers of the natural orbitals (UNO) are equally applicable for beyond BS methods such as UNO MR CI and MR CC. Therefore, they play important roles for conceptual bridges between BS and beyond BS methods as illustrated in Fig. S2.

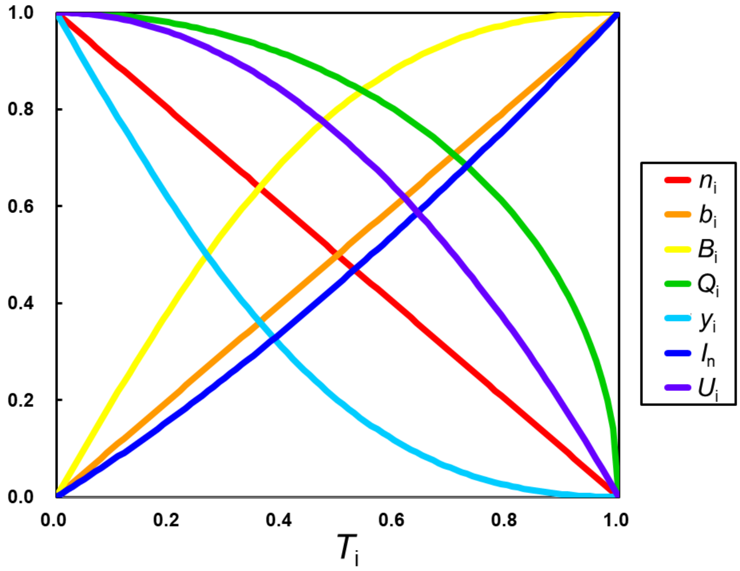

Fig. S2 Schematic illustration of variations of the chemical indices with the orbital overlap (T_i) between broken-symmetry (BS) orbitals in the BS methods coupled with spin projections. Spin density (green line) increase sharply even in the large orbital-overlap region (T_i = 0.7 ~ 0.8), indicating that several other chemical indices are examined to examine the nature of chemical bonds of SCES such as **1**.

SI Part III Spinel Mn solids, Spin frustrations and Triangular and Cubane-type clusters

The triplet double bond dissociates into triplet two fragments

In this section, the non-collinear spin structure is introduced to describe the dissociation of formal double bond in the ground triplet state into two triplet species under the BS MO model. The molecular oxygen (O=O) and Fe(IV)=O bond are the ground triplet species with the total effective bond order b = 2. The dissociation of triplet O=O provides triplet oxygen atom (^{3}O) and singlet oxygen atom (^{1}O) under the up- and down spin- (collinear spin) model as shown in eq. (s1b) in accord with the dissociation of the singlet σ-bond. However, triplet O=O dissociates into two triplet ^{3}O atoms in the ground triplet state, indicating the spin rotation from up-spin (↑) to the horizontal (→) spin and from down-spin (↓) to the horizontal (→)

spin at the right oxygen site to form the ^{3}O atom under the non-collinear (canting) spin model as shown in eq. (s1c). The situation is the same for the dissociation reaction of triplet Fe(IV)=O bond as shown in eqs. (s2b) and (s2c).

↑•O–O•↑ → ↑•O….O•↑ → ^{3}[↑•O•↑ + ↓•O•↑] (s1b)

↑•O–O•↑ → ↑•O….O•↓ → → ^{3}[↑•O•↑ + →•O•→] (s1c)

↑•Fe(IV)–O•↑ → ↑•Fe(III)….O•↑ → ^{3}[↑•Fe(II)•↑ + ↓•O•↑] (s2b)

↑•Fe(IV)–O•↑ → ↑•Fe(III)….O•↓ → → ^{3}[↑•Fe(II)•↑ + →•O•→] (s2c)

Thus, the concept of general spin structures becomes necessary under the general BS model.

Peculiar nature of chemical bonds of Mn oxides and possible isospin organic models

In this section, the magnetism and chemical bond of the Mn oxides are briefly reviewed in relation to non-collinear spin structures of metal oxides. In 1960s, magnetic properties of Mn oxides had been accepted great interest because of the so-called spin-frustrated (SF) non-collinear spin structures such as triangular and helical spin structures, etc. as illustrated in Fig. 5 in the main text. We have interested in these beautiful structures, proposing general spin structures of organic radicals the basis of the isospin analogy (Yamaguchi 1975b). The triangular cluster in A of Fig. 5 and tetrahedral cluster in B of Fig. 5 were examined as the smallest examples of such SF materials. The spin vector model characterized by the magnetic group (S × T; see later) was first applied to elucidate the spin structures for these clusters as shown in Fig. 5 (Yamaguchi 1975b). The possible broken-symmetry (BS) solutions such as UHF (one dimensional (1D) spin), GHF with 2D spin structure (see A of Fig. 5), and GHF with 3D spin structure (see B of Fig. 5) (see Fig. S1) were also constructed on the basis of the magnetic double group consisted of spatial symmetry (P_n), spin rotation (S), and time-reversal (T) symmetry (P_n × S × T) as illustrated in Fig. S3 (Yamaguchi et al. 1990b). The relative stabilities of these solutions for these clusters were examined by obtaining the analytical solutions of the Hubbard models.

In 1970s, we have obtained the analytical solutions after symmetry projections relating to P_n, S, and T (so-called extended GHF MO solutions) by the Hubbard model, demonstrating the triangular and tetrahedral spin correlations expressed by A and B in Fig. 5. On the other hand, Anderson has developed the resonating valence bond (RVB) model for these clusters (Anderson 1973). Thus, cubane-structure has been a very important structure in both physics and chemistry, providing key basic BS concepts and computational methods in Fig. S1. At that time, group-theoretical bifurcation theory has been developed in the field of mathematics.

Cubane-like multi-center structures for Mn oxides and Iron-sulfur complexes

In this section, chemical synthesis of the cubane-like cluster is reviewed briefly. In 1980s, the SF cubane-structure (Yamaguchi 1975b) became a chemical interest. Brudvig and Crabtree first proposed the cubane-like Mn_4O_4 cluster as a possible catalytic model for water oxidation in OEC of PSII (Brudvig and Crabtree 1986). An adamantine-like cluster Mn_4O_6 was indeed synthesized for the model as shown in Fig. 5 (Wieghardt et al. 1983). The Mn_4O_6 cluster was also assumed as an important intermediate for oxygen evolution (Brudvig and Crabtree 1986). These clusters are regarded as typical SF molecules in the molecular magnetism. In late 1980s and early 1990s, triangular and butterfly Mn clusters were also synthesized as model complexes of the catalytic sites for water oxidation as shown in Fig. 5 in the main text (Jang et al. 1989; Hendrickson et al. 1992; Vincent et al. 1989). The XRD experiments of these Mn complexes by Hendrickson and Christou group have elucidated the Mn-Mn and Mn-O distances, elucidating the Jahn-Teller (JT) effect of the Mn(III) ion for the elongation of the Mn-O bonds. They have also performed the magnetic susceptibility and EPR experiments of the Mn model complexes, elucidating the SF effects for the exchange-coupled Mn complexes. Therefore, the cubane-like Mn clusters synthesized chemically have been interesting materials for single molecule magnets (Sessoli et al. 1993) and catalytic model clusters for OEC of PSII (Rüttinger and Dismukes 1997).

Historically, iron-sulfur clusters such as triangular Fe_3S_4 (A in Fig. 5) and cubane-like Fe_4S_4 clusters (B in Fig. 5) (Calvin 1976) have been investigated before chemical synthesis of Mn model clusters in Fig. 5. Possible collinear and non-collinear BS solutions for Fe_4S_4 were constructed by the magnetic double group symmetry ($P_n \times S \times T$) as illustrated in Fig. S3 (Yamaguchi et al. 1990b). Recently, Fe_2S_2 and Fe_4S_4 clusters have been attracted great interest in relation to one electron transport in metalloenzymes such as PS I in photosynthesis. The magnetic properties of the cubane-like synthetic clusters with spin frustrations (SF) have been summarized in our previous review (Yamaguchi et al. 1999) (see also Table S1). Thus, magnetism and chemical bonds of the cubane-like clusters are closely related to the photosynthesis.

Developments of the chemical synthesis of the Cubane-like complexes and magnetic properties

In this section, a classical spin vector model is introduced to explain the non-collinear spin structure. In 1990s, theoretical investigations of spin frustration systems were crucial in both fields of molecular magnetism and photosynthesis. Spin frustration systems in the field of the molecular magnetism are expressed by noncollinear spin alignments under the classical Heisenberg model,

$$H_{classical} = -2\, J_{ab}\, S_a\, S_b \cos\theta_{ab} \qquad \text{(s17)}$$

where θ_{ab} denotes the torsional angel 0° < θ_{ab} < 180° between spin vectors as illustrated in Fig. 5 in the main text (Yamaguchi 1975b; Yamaguchi et al. 1990a, b). On the other hand, ferromagnetic and antiferromagnetic spin alignments are expressed by collinear parallel (θ_{ab} = 0°) and antiparallel (θ_{ab} = 180°) spin alignments, respectively (for example, see eqs. (s1c) and (s2c). Therefore, $S_a\, S_b \cos\theta_{ab}$ is regarded as a classical expression of the spin correlation function in eq. (s15). Triangular and tetrahedral spin alignments in Fig. 5 have been regarded as schematic expressions of antiferromagnetic (singlet-type; 90° < θ_{ab} < 180°) spin correlations. In 1990s, effective exchange integrals for spin frustrated transition metal clusters have been determined by EPR and related magnetic methods as shown in Table S1 (Yamaguchi et al. 1999). Therefore, the torsional angles (θ_{ab}) between spin vectors for these complexes are obtained by using J_{ab} values (see Table S1).

Table S1 Effective exchange integrals between transition metal ions in the multinuclear transition complexes.

System	J [cm^{-1}]	θ_{ij}	Alignments
$[Mn(III)_2O(O_2CMe)_2L$	$J = 9$	$\theta = 0°$	axial
$Mn(IV)_4O_6(bipy)_6(ClO_4)_4H_2O$	$J_1 = -88, J_2 = -134$	$\theta = -180°$	axial
$[Mn(II)Mn(III)_2O(O_2CMe)_6(pyr)_3](pyr)$	$J_1 = -8.3, J_2 = -5.1$	$\theta_{12} = 0°, \theta_{13} = 180°$	axial
$[Mn(III)O(O_2CMe)(pyr)_3]_3(ClO_4)_2$	$J_1 = J_2 = -10$	$\theta = 120°$	triangular
$[Mn(IV)_3O_4(OH)(bpea)_3](ClO_4)_3$	$J_1 = -11, J_2 = -76$	$\theta = 94.15°$	helical
$[Mn(IV)_3O_4(H_2O)_4(bpy)_4]^{4+}$	$J_1 = -49, J_2 = -91$	$\theta = 105.62°$	helical
$[(Mn(III)Mn(IV)O_2)_2(tphn)_2]^{4+}$	$J_1 = -101.1, J_2 = -8.4$	$\theta_{ij} = 90°$	axial
$[Mn(II)\{Cu(II)(oxpn)_3\}](ClO_4)_2$	$J = -13.3$	$\theta_{ij} = 120°$	axial
$[Mn(III)_4O_2(O_2CMe)_7(bipy)_2]^{+}$	$J_1 = -7.8, J_2 = -23.5$	$\theta_{13} = 99.6°$	helical
$[Mn(II)_2Mn(III)_2O_2(O_2CMe)_6(bipy)_2]$	$J_1 = -1.97, J_2 = -3.12$	$\theta_{13} = 113.2°$	helical
$[Mn(IV)Mn(III)_3(\mu\text{-}O)_3(\mu\text{-}Cl)]$	$J_1 = 11.3, J_2 = -13.1$	$\theta_{12} = 0°, \theta_{34} = \pi$	axial
$[Mn(II)_4Te_4]$ (cubane)	$J = -21.7$	$\theta_{ij} = 109°$	T_d
$[Mn(II)_4(\mu\text{-}OR)_4]$ (cubane)	$J_1 = -2.5 \sim 0.25$	$\theta_{ij} = 109°$ or $\theta_{12} = \theta_{34}$	T_d
$[Mn(IV)_4O_6]^{4+}$ (adamantane)	$J = 6.9$		axial

Developments of the GSO HDFT program and GSO computations

In this section, a general broken-symmetry (BS) MO model for metal complexes with the non-collinear

spin structures is explained briefly. In 1990s, general molecular orbital models have been necessary for understanding of electronic and spin states of the spin frustrated clusters for both molecular magnetism and chemical reactions. The BS solutions with 1D spin structure obtained by the HOMO-LUMO mixing (see Fig. 1) are often unstable in spin frustrated systems, reorganized into general BS solutions with 2D or 3D spin structures described by general spin orbitals (GSO), so-called two component spinor, which are obtained by the HOMO- SOMO-LUMO mixing (Yamaguchi et al. 1990a,b; Yamanaka et al. 2003a). However, the GSO DFT solutions were not obtained by using conventional Gaussian program package at that time. Therefore, we have developed our own program system that can treat all the possible types of BS solutions for cubane-like clusters at the level of hybrid DFT approximation as illustrated in Fig. S3 (Yamanaka et al. 2000). The magnetic group theoretical classifications of the GSO hybrid DFT (HDFT) solutions are shown in Fig. S3. GSO DFT solutions for cubane-like structures indeed provided tetrahedral type non-collinear spin structures (namely three-dimensional spin densities in Fig. S3), as illustrated in Fig. 5 (Yamanaka et al. 2003a, b).

Dismukes et al. (2009) have investigated the structure and reactivity of cubane-like Mn_4O_4 clusters. The GSO DFT model in Fig. S3 is applicable to investigate the evolution of triplet molecular oxygen ($^3O{=}O$) from the cubane-like Mn_4O_4 cluster in Fig. 5D, providing [the open-cubane cluster in Fig. 5H + $^3O{=}O$] (Yamanaka 2003a, b). The non-collinear models in Fig. 5 are conceivable for Fe5 cluster in Fig. 5I. On the other hand, the DODS-type collinear models such as HDFT are applicable under the distorted cubane-like cluster involving the Jahn-Teller Mn(III) ion (see Fig. 7 in the main text).

The exact diagonalizations of the quantum Heisenberg models constructed of the J_{ab} are necessary to elucidate the quantum energy levels for Mn clusters with spin frustrations. The ground spin state of the Mn_3O_4 (see Fig. 5) cluster is doublet (S_{total} = 1/2) for both Mn(II) (S_{local} = 5/2) and Mn(IV) (S_{local} = 3/2) in accord with all the antiferromagnetic exchange interactions (negative spin correlation functions in eq. (s15)) between Mn ions. On the other hand, the ground spin states become S_{total} = 5/2 and S_{total} = 3/2 for collinear ground state, respectively. Both classical and quantum spin Hamiltonian models are useful for theoretical investigations of the electronic and spin states of SCES such as **1** in Fig. 2. Thus, theoretical procedures such as BS HDFT and GSO HDFT have been developed for Mn oxide clusters before the HR XRD experiments (Umena et al. 2011). After the discovery, BS HDFT method has been applied to **1** as summarized in this review. The MR CI (CC) by the use of UNO by GSO HDFT is desirable for quantitative purpose as shown in Fig. S1, but extremely time-consuming for Mn oxide clusters. Therefore, such beyond DFT computations are future projects in our group.

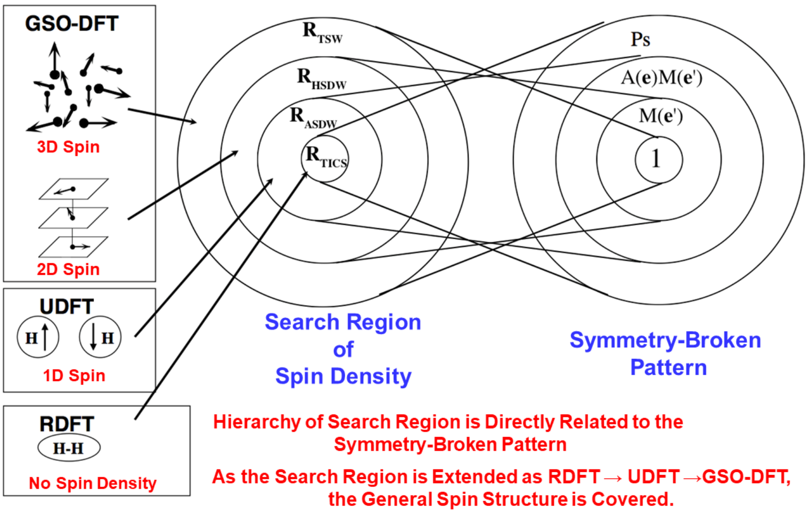

Fig. S3 Collinear and non-collinear broken symmetry solutions classified by the magnetic double groups (Yamanaka et al. 2000, 2003a, b)

Discovery of the London XRD structure

In this section, the so-called London XRD structure is examined as a typical triangular Mn_3 cluster in Fig. 5G. In 2000s, the cubane-like structure in Fig. 5 became much more important in both material and biological sciences. As mentioned in the main text, Sauer and Yachandra have examined a possible origin of the $CaMn_4$ cluster in OEC of PSII, suggesting the important roles of Mn minerals with spinel crystal structure (Sauer and Yachandra 2002). They have proposed many possible 3D structures of the $CaMn_4$ cluster for the models of the OEC of PSII. Some of the proposed $CaMn_4$ cluster based the SF spinel crystal involve the triangular Mn cluster in Fig. 5 (Sauer and Yachandra 2002).

GSO-DFT methods were first applied to elucidate the electronic and spin structure of the cubane-type Mn_4O_4 cluster (Yamanaka 2003a, b), which was investigated by Dismukes group. After just publication of the paper, the London model (Ferreira et al. 2004) with the almost equilateral triangular Mn_3O_4 fragment in the cubane-type $CaMn_3O_4$ cluster was proposed on the basis of the XRD experiments (note that the oxygen atoms were invisible by XRD at that time). Thus, the London XRD experiment provided a first example of the cubane-like $CaMn_3O_4$ cluster involved in OEC of PSII.

Therefore, we have immediately applied our BS approach to the cubane-like structure $CaMn_3O_4$ involved in the London structure (Isobe et al. 2005). The well-known triangular spin alignment with 120° of spin rotation was indeed obtained for the London model as illustrated in Figs. 5 and 6. The SF $CaMn_3$ cubane core of the London $CaMn_4O_5$ cluster (Ferreira et al. 2004) may be regarded as an extended form with a closed-cubane structure consisted of the triangular Mn_3 cluster in Fig. 5. Thus, the GSO DFT computations have revealed that the London model (Ferreira et al. 2004) is different from the spin and

valence states of the HR XRD structure later found by Umena et al. (2011). BS methods illustrated in Fig. S3 are found to be applicable for discriminations of Mn-oxides clusters with collinear and non-collinear spin structures.

The SF $CaMn_3$ cubane core of the London $CaMn_4O_5$ cluster (Ferreira et al. 2004) may be regarded as an extended form with a closed-cubane structure consisted of the triangular Mn_3 cluster in Fig. 5. After the publication of the London structure (Ferreira et al. 2004), theoretical investigations of the possible structures of the $CaMn_4O_4$ cluster with the cubane-like core were performed under the assumption of the (3344) valence structure determined by the EXAFS (Yano et al. 2005a). The Mn_1-Mn_2, Mn_2-Mn_3, Mn_3-Mn_4, Mn_1-Mn_3, and Mn_1-Mn_4 distances by the EXAFS plus MM model (Dau et al. 2008) were 3.2 (2.81), 2.7 (2.87), 2.7 (2.74), 3.2 (3.02), and 3.7 (4.34) Å, respectively, where the corresponding distances by the DFT calculations (Siegbahn 2008) are given in parentheses. Judging from the Mn_1-Mn_3 distances, these proposed structures are regarded as the open-cubane structure in agreement with their assumption of the (3344) valence state involving the JT Mn(III) ion. The Mn_1-Mn_4 distances were calculated to be extremely short, indicating the difficulty of the prediction of the HR XRD structure (Umena et al. 2011) on the basis of the London XRD structure of the protein matrix.

On the other hand, judging from the GSO DFT computations (Isobe et al. 2005), the valence state of the London structure (Ferreira et al. 2004) may be explained by assuming the (4442) structure in the S_1 state. However, the $Mn(II)_4$ site is hardly acceptable from available EXAFS results (Dau et al. 2008). Thus, the valence configuration is one of the important factors to investigate scope and reliability of the XRD and theoretical structures. Therefore, we have investigated the four degrees of freedom in Fig.2 to elucidate the geometric, electronic and spin structures of the $CaMn_4O_5$ cluster based on the HF XRD result (Umena et al. 2011) as shown in the main text.

Recent developments of the SF solids and their application to catalysts for water oxidation

In this section, according to the referee' comments, applicability of metal oxides involving the cubane-like cluster to water oxidation is touched briefly. In 2010s, spin frustrated (SF) materials have been attracted great interest in both material science and artificial photosynthesis. Several new magnetic phases have been discovered in the Kagome and super Kagome lattices consisted of the triangle clusters (A in Fig. 5) and in pyrochlore crystals consisted of the tetrahedral cluster (B in Fig. 5). These materials exhibit the spin frustration (SF) effect (Anderson 1973; Yamaguchi 1975b, 1990b) in accord with the SF structures in Fig. S3. Therefore, many spinel compounds have been investigated in relation to the new concepts such as spin liquid state in the solid-state physics. Applications of these materials to development of effective catalysts for water oxidation are also interesting.

Indeed, spinel SF $LiMn_2O_4$ solid with the mixed valence structure (3.5 = (4 + 3)/2) has been investigated to elucidate chemical reactivity, indicating that the Mn_4O_4 core is generated in the catalytic site of the system for water oxidation (Robinson et al. 2010). Therefore, reexaminations of recently developed SF materials such as pyrochlore crystals may be future interesting directions for search of new water oxidation mesoscopic catalysts consisted of the earth-abundant 3d transition metal oxides since molecular clusters complexes in Fig. 5 are not so stable even in the protein matrix in OEC of PSII. Thus, fundamental finding of the native OEC of PSII provide working hypotheses of design of robust artificial catalysts for water oxidation (Isobe et al. 2012).

SI Part IV Kok cycle for water oxidations

High-oxidation scenario

In this section, the charge degree of freedom in Fig. 2 is examined in relation to the valence state of the $CaMn_4$ cluster in OEC of PSII. Joliot (Joliot et al. 1969) and Kok (Kok et al. 1970) have first elucidated that five steps S_i (i = 0 ~ 4) are necessary for water oxidation in OEC of PSII as shown in Fig. S4. Therefore, theoretical investigations of possible intermediates in the whole Kok cycle are crucial for understanding and explanation of the mechanisms of water oxidation. Concerning with the charge degree of freedom in Fig. 2, both high- and low-oxidation scenarios for valence states of Mn ions in the Kok cycle have been proposed on the experimental grounds (Pace et al. 2012). In our papers, the valence state of the Mn ions is examined under the assumption of the high-oxidation scenario which provides the $Mn(III)_2Mn(IV)_2$ valence configuration (Yachandra et al. 1996) for the dark stable S_1 state (Isobe et al. 2012). The valence states of the S_0 and S_2 are obtained by the one electron reduction and oxidation of the S_1 state, respectively, providing the $Mn(IV)Mn(III)_3$ and $Mn(IV)_3Mn(III)$ configurations as shown in Fig. S4. However, the electron transfer (ET) processes are often coupled with proton transfer (PT) processes in biological systems such as OEC of PSII. In fact, a proton is released in the S_0 to S_1 transition as shown in Fig. S4. On the other hand, a proton is not released to lumen in the case of the S_1 to S_2 transition.

Both 3H (W2 = OH^-) and 4H (W2 = H_2O) models have been proposed for the dark stable state in the Kok cycle. We have assumed the 4H model for the S_1 state of **1**, $Ca(II)Mn(III)_2$ $Mn(IV)_2(H_2O)_4$ (Kanda et al. 2011b; Yamanaka et al. 2011; Isobe at al. 2012), providing the $Ca(II)Mn(III)_1$ $Mn(IV)_3(H_2O)_4$ for the S_2 state with the low spin configuration (S = 1/2) because of one electron release. On the other hand, the high-spin (S = 5/2) S_2 state may be formed by the deprotonation of W2 for which the generated proton is trapped with base (B), namely BH^+, providing $Ca(II)Mn(III)_2Mn(IV)_2(OH)(H_2O)_3$

of 1_{s2} under the assumption of no proton release to lumen in the physiological condition (Shoji et al. 2015c, 2019b).

Mutation experiments have provided critical information on the protonation states of coordinated water molecules to **1** (Debus 1992). Interestingly, proton release has been observed in the S_1 to S_2 transition when Val 185 has been mutated with threonine (Thr) with the OH group, which can form the hydrogen bonding interaction of the deprotonated W2 site (W2 = OH^-) of 1_{s2}. The EPR spectroscopy has elucidated the formation of the high spin (S = 5/2) S_2 state of the V185T mutant (Sugiura et al. 2018). W2 site is also linked with the hydrogen bonding network, Asp61→W8→W9→W2→W5→W6 (orW3) →W7→Tyr161, which is compatible with the EPR results for the mutant (Sugiura et al. 2018). Thus, the 4H model (Yamaguchi et al. 2016; Miyagawa et al. 2021) is flexible enough for understanding and explanation of complex behaviors revealed by the EPR results in the S_2 state (Boussac 2019).

Proton release from W1 followed by water insertion occurs in the S_2 to S_3 transition (Shoji et al. 2015), providing the S_3 intermediate as shown in Fig. S4. The same S_3 intermediate was formed even in the case of the V185T mutant, exhibiting the common S_{3abca}(R)-OH structure between V185 and V185T in accord with the DLPNO CCSD(T_0) results in Table 5. Proton release from W1 of the S_3 intermediate occurs in the S_3 to S_4 transition, entailing the O-O bond formation at the transient S_4 state. Interestingly, the rate of the O-O bond formation is significantly slowing down for the V185T mutant (rate change was from 1 ~ 2 ms to 20 ms), indicating a serious variation of the hydrogen-bonding network around the Mn_4 ion, for example variation from W2 = OH^- to W2 = OH^-….HO-group of Thr, reducing a proton accepting function for the O-O bond formation in the S_4 state. Proton and molecular oxygen are released in the S_4 to S_0 transition as shown in Fig. S4. Thus, the mutation experiments provide crucial information for elucidation of the hydrogen bonding networks for water oxidation (Shoji et al. 2015b).

The valence state of the S_3 state is observed to be $Mn(IV)_4$ (Yano and Yachandra 2014) in accord with hydroxide inserted S_3 structure (see Table 3). On the other hand, a proton coupled electron transfer (PCET) from Mn-hydroxide becomes feasible at a high temperature (Renger 2012), providing the Mn-oxyl structure with the $Mn(IV)_3Mn(III)$+O• valence state in Fig. S4 (Yachandra et al. 1996) (see details below). Two protons are released in the S_3 → [S_4] → S_0 transition for which deprotonation of BH^+ occurs to afford B. The timing of the electron transfer from 1_{s3} to Tyr161-radical in this transition is important to understand possible mechanisms of water oxidation as examined in the text (see also SI. Parts VII and VIII. Therefore, dynamical mechanisms of proton release in the final transition are under current investigation (Bhowmick et al. 2023; Greife et al. 2023). The 4H model is flexible enough for understanding and explanation of the 1:0:1:2 (1:1:1:1) pattern of proton release in the Kok cycle for wild (mutant) systems.

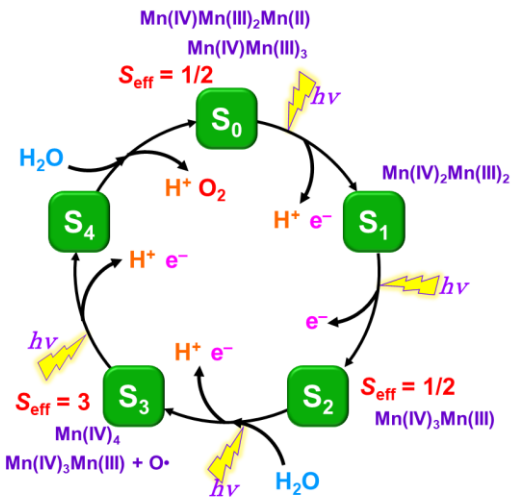

Fig. S4 Kok cycle (Kok et al. 1970) for water oxidation in OEC of PSII; One electron oxidation of the $CaMn_4O_5$ cluster (**1**) occurs four times for the formation of total four holes formation for water oxidation. The valence and spin states of **1** are shown under the assumption of the high-oxidation scenario for the S_i (i = 1 ~ 4) states, namely the $Mn(III)_2Mn(IV)_2$ valence state for the dark stable S_1 state. Possible pathways for water inlet and proton release in the Kok cycle are discussed in this supporting materials.

Discovery of substrate water molecules by HR XRD: Possible reaction pathways

In this section, we revisit early computational results for XRD structure discovered in 2011 in relation to the referee's comments for water insertion in the S_2 to S_3 transition. Two water molecules are necessary for oxygen evolution in the Kok cycle as illustrated in Fig. S4. In 2011, Umena et al. have first observed water molecules in OEC of PSII by the high-resolution (HR) XRD method (Umena et al. 2011). The three-dimensional (3D) HR XRD structure (Umena et al. 2011) has revealed water molecules around the $CaMn_4O_5$ cluster as illustrated in Figs. 14-17. According to the HR XRD structure, they have proposed that three important water molecules, two coordinated water molecules (W2, W3) and $O_{(5)}$ site of the $CaMn_4O_5$ cluster play important roles for water oxidation reaction in OEC of PSII (Umena et al. 2011). Therefore, our first collaborative work (Yamanaka et al. 2011) with the Shen-Kamiya group was to confirm their proposal.

We first performed the QM (HDFT) computations of the $CaMn_4O_5$ cluster with the distorted chair-like XRD structure (Umena et al. 2011) in order to elucidate possible reaction pathways (Yamanaka et al. 2011). To this end, the BS DFT (UB3LYP) computations were performed for the $CaMn_4O_5$ cluster with the (4444) valence state responsible for the S_3 state in the high-oxidation scenario. The natural orbital (UNO) analysis of the BS DFT solutions was performed to elucidate frontier molecular orbitals (FMO) of the $CaMn_4O_5$ cluster (Yamanaka et al. 2011). The 3D pictures of the FMOs have elucidated

four possible reaction pathways: (1) attack of W3 to the $O_{(5)}$ site, (2) attack of W2 to the $O_{(5)}$ site, (3) attack of W1 to the $O_{(4)}$ site, and (4) attack of W3 to the $Mn_4{=}O$ bond formed with the reaction; $Mn_4(OH)_2 \rightarrow Mn_4{=}O(OH_2)$. Thus, the UNO (FMO) analysis has elucidated important water molecules (W1, W2, and W3) and the oxygen sites ($O_{(5)}$ and $O_{(4)}$) for water oxidation catalyzed by the $CaMn_4O_5$ cluster (Yamanaka et al. 2011), supporting the proposal based on the HR XRD structure (Umena et al. 2011).

HDFT computations of possible intermediates in the S_2 and S_3 states: Early results

In this section, HDFT computations on the possible S_2 and S_3 intermediates are revisited briefly. The HR XRD structure (Umena et al. 2011) was obtained for the dark stable state in the Kok cycle. However, the X-ray damage (Yano et al. 2005a; Galstyan et al. 2012) of the $CaMn_4O_5$ cluster was pointed out as a serious problem at that time (~2011). Therefore, we have constructed possible 48 BS solutions for the $CaMn_4O_5$ cluster by changing the six valence states and eight spin states in the S_1 state (Yamanaka et al. 2012), confirming the (3443) valence state in the ground S_1 state (Umena et al. 2011). The refinements of the HR XRD structure (Umena et al. 2011) by the full geometry optimizations were also crucial as summarized in a recent review article (Yamaguchi et al. 2022a). The details are not repeated here for brevity.

Unfortunately, there are no reliable XRD structures for the S_2 and S_3 states in the Kok cycle at that time (~2011). Therefore, we have performed full geometry optimizations of the possible intermediates in the S_2 state, assuming the main skeleton of the $CaMn_4O_5$ cluster (Isobe et al. 2012). Interestingly, both left (L)- and right (R)-opened geometric structures were obtained, depending on the (4443) and (3444) valence structures of the $CaMn_4O_5$ cluster. Two different European groups (Pantazis et al. 2012; Guidoni, Bovi, Narzi et al. 2013, 2014) have also performed theoretical investigations of the S_2 state, elucidated similar important and interesting results. Thus, the Mn_4-$O_{(5)}$-Mn_1 bond was labile in accord with the UNO analysis of the nature of the chemical bonds of the $CaMn_4O_5$ cluster (Kanda et al. 2011a).

According to the Kok cycle, water insertion has been assumed in the S_2 to S_3 transition. Therefore, we have examined the water insertion in the S_3 state, elucidating the full-optimized structure of the left-opened (L) S_3 structure with the inserted hydroxide ($O_{(6)}H$) (Isobe et al. 2012). Therefore, the L-type Mn-hydroxide was confirmed as a possible S_3 intermediate on the theoretical ground. The full geometry optimizations of the right-opened (R) S_3 structure with the inserted hydroxide ($O_{6)}H$) have also been performed to elucidate the valence and spin structures of the S_3 intermediates (Isobe et al. 2014). Thus, full geometry optimization by HDFT has been useful for elucidation of possible intermediates in

the S_3 state.

QM/MM calculations for water input and proton-release pathways in OEC of PSII

In this section, theoretical investigations of bio-molecular system structures for water oxidation in OEC of PSII are revisited. Our theoretical computations (Isobe et al. 2012, 2014) have elucidated the hydroxide inserted S_3 structures in the Kok cycle. According to the original proposal based on the HR XRD (Umena et al. 2011), W2 or W3 may be inserted into the labile Mn_4-$O_{(5)}$-Mn_1 bond. Therefore, theoretical investigations on the water inlet pathway (WIP) and proton release pathways (PRP) were essential at that time (~ 2012). To this end, QM/MM computations were performed for several model clusters as shown in Figs. 14-17. The QM/MM computational results (Shoji et al. 2013) have elucidated an important role of Val185 in front of the $O_{(5)}$ site, indicating the hydrophobic reaction field near the $O_{(5)}$ site of the $CaMn_4O_5$ cluster. Therefore, the QM/MM-HR XRD structure suggests that the insertion of an extra water molecule in front of the $O_{(5)}$ site is hardly possible for proton transfer although Siegbahn has considered such a model in his early paper (Siegbahn 2012); therefore, he has revised his model later (Siegbahn 2018). Interestingly, Dilbeck et al. have performed the mutation experiment of Val185, demonstrating that the mutation of Val185 has a strong effect on the water oxidation mechanism (Dilbeck et al. 2013).

The QM/MM-HR XRD computations have elucidated the WIP and three PRPs as illustrated in Figs. 14-17. The HR XRD structures (Umena et al. 2011) have elucidated inclusions of several glycerol molecules in the large channels (A, B, and C), in turn indicating the possible channels for water insertion (Shoji et al. 2015a, b). The QM/MM computations have also revealed the water wire linked with the $CaMn_4O_5$ cluster ; (A, B, C) → W24 → W23 → W22 → W21 → W10 → W4 → W3 → W5 → W2 → W9 → W8 → Asp61 → W12 → W13… (in our notations) for the WIP and PRP I. Therefore, several proposals have been presented to obtain an answer for the question; “ Which water molecule is inserted as the $O_{(6)}H$ hydroxide in the S_2 to S_3 transition? Shoji et al. (2015c) has performed the QM/MM computations, proposing that W3 is a candidate for the inserted $O_{(6)}$ hydroxide in both L- and R-opened S_3 structures.

Multiple intermediates models for the S_3 state

In this section, multiple intermediates models for the S_3 state are examined briefly. One of the authors (KY) obtained e-mail from Prof. Gernot Renger (2012), which illustrated his multiple S_3 models based on the spectroscopy (Renger 2012). Formations of two hydroxide bonds were assumed in a possible S_3 intermediate of the Renger models (Renger 2012). We feel that this may indicate the formation of hydroperoxide (HOOH) as a two-electron oxidation product in special reaction conditions. On the other

hand, the spin density populations by the QM computations on the $CaMn_4O_5$ cluster (Yamanaka et al. 2011) have indeed elucidated a possibility of the $O_{(5)}$-$O_{(4)}$ bond formation like in the first proposal of the radical coupling by Yachandra et al. (Yachandra et al. 1996). Therefore, we must examine a possibility of the O-O bond formation in the S_3 state (Yamaguchi et al. 2014). Isobe et al. performed full geometry optimizations (~2014) of several possible S_3 intermediates, proposing an equilibrium model for the S_3 state (Isobe et al. 2016). However, three years were necessary for the publication of our paper because of several referee's comments against the proposal (Isobe et al. 2016). In the same year, Berkeley group (Young et al. 2016) published the XFEL results claiming the no water insertion in the S_2 to S_3 transition. Therefore, the QM/MM computations were performed for both S_3 intermediates with no water insertion and with water insertion (Shoji et al. 2017), showing that the geometric structure by the XFEL SFX (Young et al. 2016) is rather similar to the water-inserted structure (see also the refined XFEL SFX paper consistent with the water-inserted structure (Kern et al. 2018).

Water insertion in the S_2 to S_3 transition in the Kok cycle : Recent results

In this section, recent proposals for water insertion pathways are reviewed briefly. After the discovery of the HR XRD structure (Umena et al. 2012, many proposals have been published for the mechanisms for water oxidation in OEC of PSII. According to the referee comments, we here review several proposals relating to water insertion mechanisms in the S_2 to S_3 transition in Fig. S4. Recent time-resolve XFEL SFX results have elucidated the multi-step processes in the S_2 to S_3 transition: (1) generation of Tyr161-O radical with the second flash, (2) proton release for the $CaMn_4O_x$ cluster, (3) one electron transfer from the $CaMn_4O_x$ cluster to Tyr161-O radical, and (4) water insertion into the $CaMn_4O_x$ (x = 6) cluster, providing the S_3 intermediates. Retegan et al. have detected two S_3 intermediates; one is the EPR active intermediate with S = 3 spin state and the other is the EPR inactive intermediates (Retegan et al. 2016). The former S_3 intermediate detected by EPR was assigned as the right (R)-opened Mn-hydroxide; S_{3abca}-$O_{(6)}$H investigated by the CAM-B3LYP and CASSCF computations of the optical excitation and EPR results. They have proposed a water insertion via the PRP I (Cl1 channel; see also Fig. 15) in our notation.

Askerka et al. have proposed a carousel rearrangement of water ligands around Mn4 which is described as follows (Askerka et al. 2015) ; water molecule in the narrow water network (PRP III in our terminology (Shoji et al. 2015)) → W2 → W1 → W2 → W6 coordinated to the Mn4 site. Their proposal is essentially related to the EPR experiment that predicts NH_3 molecule at the W2 site of the $CaMn_4O_x$ cluster, indicating flexible nature of water molecules coordinated to the Mn4 site. The QM/MM computational results have also supported their proposal (Askerka et al. 2015). Wang et al. have extended

the carousel mechanism, proposing the proton release from the inserted $O_{(6)}H_2$ of the L-opened S_{2acca} intermediate followed by the electron release to provide the L-opened S_{3acca}-$O_{(6)}H$ intermediate (Wang et al. 2107). The proton shift from the $O_{(6)}H$ to the $O_{(5)}$ site is assumed at the final stage, providing the R-opened S_{3acca}-$O_{(6)}H$ intermediate. According to their analysis of the available XFEL results for the S_1 and S_3 states, their carousel proposal is consistent with these experimental results. They have proposed a water insertion via the PRP III (O4 channel or narrow channel; see also Fig. 17) in our notation.

Capone et al. have performed the DFT+U computations, showing that the passage between the L-opened S_2 structure S_{2abca} and R-opened S_3 structure is possible via a reorganization between the W2 hydroxy group and the μ-oxo $O_{(5)}$ group (Capone et al. 2015). The water insertion and proton release occurs in their model after the formation of the (4444) valence state. They have further performed the minimum energy path (MEP) nudged elastic band (NEB) calculations for four different water insertion processes; XW_1, XW_2, XW_3, and XW_4 (Capone et al. 2016). Judging from the calculated activation barriers, they have concluded that the XW_2 and XW_3 pathways are energetically acceptable as compared with the XW_1 and XW_4 pathways. However, they have pointed out that the XW_3 pathway is not favorable because of several reasons (Capone et al. 2016), in turn supporting the XW_2 pathway. Therefore, their conclusion is consistent with the proposal by Retegan et al. (2016), namely water insertion via the PRP I (Cl1 channel; see also Fig. 15) in our notation.

Very recently, Okamoto et al. have examined the effects of D1-N298A mutation and NO_3^- anion substitution for Cl^-, which perturbed the WIP (O1 channel) and PRP I (Cl1 channel) pathways, respectively, on the $S_2 \rightarrow S_3$ kinetics using the time-resolved infrared (TRIR) spectroscopy (Okamoto et al. 2021). Interestingly, the $S_2 \rightarrow S_3$ transition was retarded upon the D1-N298A mutation, indicating a fast geometry change around Y_z instead of a long-range proton transfer via the PRP II (Y_z channel; see Fig. 16). The TRIR experimental results obtained by fitting the IR absorption changes have elucidated that the proton release pathway in the $S_2 \rightarrow S_3$ transition is the PRP I (Cl1 channel), which is consistent with the observation that Cl^- depletion fully blocks the $S_2 \rightarrow S_3$ transition. The TRIR results have further supported the proposal that W3 ligated to the Ca(II) ion is inserted into the $O_{(6)}$ (O_x) site and W3 is replenished by water delivery through the WIP (O1 channel).

Siegbahn has not taken into account of Val185 in this early paper (Siegbahn 2012). However, he also pointed out the important role of Val185 in front of the $O_{(5)}$ site because of the mutation experiments (Dilbeck et al. 2013) by in his revised paper (Siegbahn 2018), investing rotation of Val185 for reduction of the repulsion effect for water insertion. He further performed the UB3LYP* computations of the S_2 and S_3 intermediates, indicating that the L-opened closed-cubane structure is never involved in the water insertion event in the $S_2 \rightarrow S_3$ transition because of the high energy (about 18 kcal/mol) (Siegbahn 2018) against the carousel mechanism (Wang et al. 2017) and the QM/MM results

(Capone et al. 2016). However, according to his computational model, W3 insertion to the Mn_1 site was energetically feasible under the assumption of the Mn(III)Mn(IV)$_3$ valence state instead of the Mn(IV)$_4$ state. On the other hand, recent XFEL SFX results (Hussein et al. 2021) have elucidated the proton coupled electron transfer process for water insertion.

Thus TR IR (Okamoto et al. 2021) and TR XFEL SFX (Bhowmick et al. 2023) experiments have provided important information for the dynamic water insertion in the S_2 to S_3 transition. The QM/MM computational results (Shoji et al. 2015c) have small energy difference between the L- and R-opened pathways for W3 insertion in compatible with the TRIR result. However, as mentioned above, several different S_3 intermediates have been proposed on both theoretical and experimental grounds. The TR XFEL SFX results for the S_3 state at about 2.0 Å resolution are not conclusive yet. Therefore, we have decided to perform full geometry optimizations of all the conceivable S_3 intermediates from four degrees of freedom (spin, charge (mixed valence), orbital (Jahn-Teller effect), and nuclear motion involving proton shift) by the HDFT methods. The EPR parameters for the optimized S_3 structures have been also calculated for comparisons with available experimental results. Relative stabilities among the S_3 intermediates have been elucidated by several DFT methods and DLPNO CCSD(T_0) methods. These computational results are useful as reference data for consideration of time-dependent sequential variations of several S_3 structures in the water-insertion processes investigated by the above all the experimental and theoretical articles.

Two possible valence structures in the S_3 state

In this section, possible valence structures in the S_3 state are revisited. Historically, two different proposals have been presented for the ET process in the S_2 to S_3 transition. Early XAS and EXAFS experiments (Yachandra et al. 1996) have elucidated the no change of the valence state of the Mn ions in the S_2 to S_3 transition, proposing the Mn-Oxyl radical structure, Mn(IV)$_3$Mn(III)-O• as shown in Fig. S4, for which the one electron oxidation occurs at ligand site (O•) instead of the Mn ion. On the other hand, recent XES and related experiments (Yano and Yachandra 2014) have elucidated the Mn(IV)$_4$ valence configuration in the S_3 state as illustrated in Fig. S4. The HDFT computations (Isobe et al. 2016) have elucidated small energy difference between these two different structures, suggesting the mixing in the S_3 state at room temperature. On the other hand, DLPNO CCSD(T_0) computations based on the intermediate QM (227 atoms) model have indicated the great stability of the Mn-hydroxide than the Mn-oxyl intermediate at the zero temperature as shown in the main text. However, the QM (227 atoms) model might be small to obtain conclusive results for water oxidation. Therefore, possible mechanisms for the O-O bond formation at the $S_3 \rightarrow [S_4] \rightarrow S_0$ transition have been discussed on the basis of the DLPNO

CCSD(T_0) computational results at the current level, providing multiple mechanisms discussed in the main text. Further interplay between theory and experiments is inevitable for the goal.

SI Part V The biomolecular system structures of OEC of PSII

Naming of the key water molecules around the $CaMn_4O_5$ cluster

In this section, a large-scale QM/MM computation for elucidation of the hydrogen bonding networks is touched briefly in relation to the naming of key water molecules; W1-W27. The HR XRD experimental results (Umena et al. 2011) have first elucidated the positions of water molecules (about 2800 molecules), two chloride ions, etc. in OEC of PSII, elucidating biomolecular system structures (Saito et al.2012). Unfortunately, hydrogen atoms of these key water molecules were invisible at the 1.9 Å resolution of the HR XRD structure (Umena et al. 2011). Therefore, the oxidation states of the water molecule (oxygen dianion O^{2-} (= a), hydroxide anion OH^- (= b), and H_2O (= c)) in **1** are not elucidated on the experimental grounds. The situation is the same even for recent XFEL SFX results (Kern et al. 2018; Suga et al. 2019). Moreover, several papers (Galstyan et al. 2012; Yano et al. 2005b) have pointed out X-ray damages of the HR XRD structure. Therefore, refinements of the hydrogen bonding networks were also crucial for discussions of water inlet pathway (WIP) and proton release pathway (PRP) for water oxidation in eq. (1) at that time (2013-2015).

BS computations (Shoji et al. 2015a, b; Isobe et al. 2016) have elucidated strong couplings between **1** and hydrogen bonding networks, indicating biomolecular systems structures for water oxidation in OEC of PSII. The large-scale QM/MM of these biomolecular system structures were indeed crucial for understanding and explanations of biological functions of protein matrix. Judging from populations of water molecules, key water molecules have been found around the $CaMn_4O_5$ cluster (**1**) embedded in protein matrix (Shoji et al. 2015a, b). The two water molecules (W1000 and W999) coordinated to the Mn_4 ion have been conveniently named as W1 and W2, respectively (Umena et al. 2011). The two water molecules (W541 and W540) coordinated to the Ca ion are similarly named as W3 and W4, respectively. Many other water molecules are linked with these water molecules, forming hydrogen bonding networks around **1**. However, other key water molecules around **1** were not characterized with simplified names in the HR XRD structure (Umena et al. 2011). Therefore, we have provided conventional names of the key water molecules as W5 ~ W27 after the large-scale QM/MM refinements of the networks as shown in Fig. 14 in the main text.

Full geometry optimizations of the hydrogen bonding network by large scale QM/MM

In this section, hydrogen-bonding networks around **1** are constructed with invisible hydrogen atoms. Refinement of the hydrogen bonding networks by HR XRD was a first theoretical task for elimination of possible geometric errors and X-ray damage. To this end, we have performed full geometry optimizations of the hydrogen-bonding networks around **1** by the large-scale QM(380 atoms)/MM calculations to refine the observed H-bond networks. Table S2 summarizes the optimized distances between the heavy atoms X and Y, and the optimized H---Y distances and angles for X-H---Y of the hydrogen bonding (X-Y) systems in OEC of PSII by the QM(380 atoms)/MM computations. The hydrogen bonding distances and angles are compatible with standard values concluded from many model compounds. Therefore, the QM(380 atoms)/MM computations have indeed provided the refined network structures which have been useful and reliable to assign WIP and PRP as shown in Figs. 14-17 in the main text.

The optimized hydrogen bonding networks in the S_1 state are useful for understanding of their variations at the transition(s) from the S_1 to other state(s) in the Kok cycle. A water molecule, W16 in our nation (Shoji et al. 2015b), becomes invisible after the S_1 to S_2 transition, indicating dynamical motion in Fig. 2. However, other water molecules named in the S_1 state are observed in the S_2 state. Therefore, the same naming is applicable to understand a characteristic change of the hydrogen bond network in the S_1 to S_2 transition. The situation is also similar in the S_2 to S_3 transition except for the insertion of a new water molecule, namely $O_{(6)}$ (Suga et al. 2019) and/or O_X (Kern et al. 2018). Second invisible water molecule ($H_2O_{(7)}$) is considered to be inserted into the reaction site in the $S_3 \rightarrow [S_4] \rightarrow S_0$ transition as shown in Fig. S4. Therefore, the naming of the key water molecules in the dark stable S_1 state is useful and reliable for understanding and explanation of the observed changes of the hydrogen-bonding networks in the Kok cycle in Fig. S4. Large-scale QM(380 atoms)/MM computations (Shoji et al. 2015b) were useful and handy for refinements of the hydrogen bonding networks around **1** observed by HR XRD (Umena et al. 2011).

Table S2 The hydrogen bonding distances and angles by HR XRD and large-scale QM/MM computations.

X-Y H-bonds	R(X-Y)[a)]	R(X-Y)[b)]	R(H---Y)[b)]	Angle (X-H---Y)[b)]
W1-Asp61	2.72	2.63	1.65	165
W1-Ser169(BB)	2.77	2.82	1.76	167
W2-W5	2.75	2.90	1.94	166
W3-W7	2.75	2.77	2.06	173
W3-W5	2.92	2.70	1.97	165

W4-Tyr161	2.87	2.89	2.03	148
W4-Gln165	2.76	2.75	1.80	162
W5-Asp170(BB)	2.88	3.01	2.05	167
W6-W5	2.78	2.67	1.68	167
W6-W7	2.86	2.81	1.88	158
W7-Tyr161	2.62	2.65	1.66	171
W7-Glu189	2.75	2.78	1.79	147
W8-Asp61	2.66	2.72	1.78	159
W8-W9	2.72	2.70	1.71	170
Lys317-W8	3.03	2.63	1.58	163
W9-W2	2.93	2.85	1.86	176
W9-Asn181	2.78	2.79	1.71	170
W10-$O_{(1)}$	2.67	2.77	1.95	156
W10-W21	2.91	2.88	1.92	169
W11-$O_{(4)}$	2.50	2.77	1.78	173
W11-Asp61	2.72	2.69	1.70	169
Ser169-W11	2.73	2.85	1.87	174
W12-Asp61	2.62	2.71	1.74	167
W12-W14	2.70	2.67	1.69	167
W15-Cl1	3.03	3.1	2.13	171
Lys317-Cl1	3.39	3.2	2.24	154
Asn181-Cl1	3.34	3.37	2.36	169
Glu333(BB)-Cl1	3.03	3.41	2.44	159
W16-W11	2.76	2.8	1.84	164
W16-W17	2.71	2.74	1.76	167
W17-W18	2.73	2.83	1.84	177
W17-Asp61	2.72	2.77	1.84	160
W19-Ser169(BB)	2.76	2.88	2.07	147
W20-W10	3.00	2.76	2.05	164
W21-W4	3.84	3.05	2.09	157
His337-W25	2.84	2.82	1.91	152
W26-Cl2	3.21	3.14	2.17	168
W27-Cl2	2.87	3.16	1.92	169
His337(BB)-CL2	3.21	3.12	2.12	164

Asn338-CL2	3.72	3.54	2.24	154
Glu354-CL2	3.32	3.6	2.58	172

a) HR XRD (Umena et al. 2011) b) large-scale QM(380 atoms) /MM (Shoji et al. 2015a,b)

Basis set effects for full geometry optimizations by large-scale QM/MM calculations

In this section, the basis set dependency for the optimized geometric parameters are touched briefly. The Mn-Mn and Mn-Ca distances of **1** by HR XRD (Umena et al. 2011) were also expected to be refined by the large-scale QM(380 atoms)/MM computations (Shoji et al. 2015b) because of the strong couplings with the hydrogen bonding networks, removing out the semi-internal reduction (Yamaguchi et al. 2014). However, Lanl2DZ basis set was used for the QM/MM computations, indicating the necessity of the examination of the basis set effects. The optimized geometrical parameters for the right (R)- and left (L)-opened S_3 intermediates are summarized in Tables S3 and S4 for comparisons with present results (see the main text) by UB3LYP-D3 with de2-TZVP basis. It is noteworthy that the valence state is expressed by the d(1)c(2)b(3)a(4) order in these Tables. The optimized Mn3-Mn4 distances were 2.79 (2.77), 2.75 (2.73), and 2.81 (2.83) Å, respectively, for the R-opened Mn-hydroxide, Mn-oxo and Mn-oxyl structures, where the corresponding distances by UB3LYP-D3/def2-TZVP are given in the parentheses. Judging from the computational results for the heavy atom distances, the large-scale QM(HDFT)/MM computational results using Lanl2DZ are reliable as compared with the XFEL SFX results (Kern et al. 2018). This in turn indicates that the refined results for the hydrogen bonding networks in Table S2 by large-scale QM(380 atoms)/MM computations (Shoji et al. 2015b) are also reliable enough for examinations of WIP and PRP.

Table S3 The optimized Mn-Mn and Ca-Mn distances of the right (R)-opened intermediates in the S_3 state by the large-scale QM/MM methods.

Distances	Hydroxide	Oxo	Oxyl	Peroxo	Superoxo
Valence	(4444)	(4444)	(4443)	(4433)	(3433)
Mn_1-Mn_2	2.74	2.77	2.75	2.75	2.74
Mn_2-Mn_3	2.81	2.77	2.78	2.78	2.78
Mn_3-Mn_4	2.79	2.75	2.81	2.96	3.05
Mn_1-Mn_3	3.56	3.39	3.33	3.33	3.37
Mn_1-Mn_4	5.29	5.07	5.13	4.99	5.09

Ca-Mn_1	3.39	3.26	3.33	3.74	3.61
Ca-Mn_2	3.42	3.40	3.41	3.48	3.50
Ca-Mn_3	3.56	3.47	3.48	3.77	3.93
Ca-Mn_4	4.02	3.92	3.97	4.09	4.23

Table S4 The optimized Mn-Mn and Ca-Mn distances of the left (L)-opened intermediates in the S_3 state by the large-scale QM/MM methods.

Distances<br>Valence	Hydroxide<br>(4444)	Oxo<br>(4444)	Peroxo<br>(3443)	Superoxo<br>(3433)
Mn_1-Mn_2	2.68	2.69	2.72	2.73
Mn_2-Mn_3	2.78	2.76	2.77	2.80
Mn_3-Mn_4	2.79	2.75	2.96	3.05
Mn_1-Mn_3	2.90	2.89	3.22	3.35
Mn_1-Mn_4	5.32	5.04	5.07	5.34
Ca-Mn_1	3.41	3.41	3.61	3.54
Ca-Mn_2	3.50	3.58	3.50	3.43
Ca-Mn_3	3.42	3.41	3.74	3.72
Ca-Mn_4	4.28	3.73	3.98	4.20

SI Part VI Relative stabilities among the S_3 intermediates by DFT and beyond DFT

Scope and reliability of DLPNO CCSD(T_0) starting from UNO of HDFT

In this section, scope and applicability of UNO for trials for beyond DFT computations are examined briefly. Elucidation of relative energies among S_3 intermediates is a difficult task at HDFT level of theory as compared with the geometry optimizations (see Fig. S6). Therefore, beyond HDFT computations such as the coupled cluster (CC) model are desirable. The UHF solution has been used as the UHF coupled cluster (CC) SD(T) computations (UCCSD(T)) as shown in Fig. S1. The UCCSD(T) computations are feasible for organic radicals. On the other hand, UHF SCF computations are often hardly converged in the case of large transition metal oxides such as $CaMn_4O_6$ cluster (**1**). Alternately, unrestricted Hartree-Fock-Slater (UHFS) methods have been used for such complex systems (Slater 1972). In fact, UCCSD computations starting from UHFS solution has been proposed as an approximate procedure for metal clusters (Yamaguchi 1979). The UHFS method is considered to be a precursor method for hybrid DFT (HDFT) method that is applicable even for **1**.

According to the referee comments, we touch the computational procedures in the supporting section. The UHF SCF computation for **1** is hardly converged even for QM I (103 atoms) level, indicating that the UHF CCSD(T) computation in Fig. S1 is hardly applicable to **1**. On the other hand, HDFT SCF computations such as UB3LYP-D3 are easily converged for QM I (103 atoms) model. However, the HDFT solutions do not satisfy the Brillouin theorem (Yamaguchi 1979), indicating that the natural orbitals (UNO) of the HDFT solutions are approximate natural molecular orbitals for the CCSD(T). Therefore, large-scale multi-configuration (MC) SCF computations (Yamaguchi 1980) such as CASSCF (Roos 1980) are desirable for the refinements of the natural orbitals as shown in Fig. S1. However, such computations for **1** are hardly possible in our group. Therefore, we first examined variations of relative energies among the S_3 intermediates by using UNO (or localized version of UNO (ULO)) obtained by hybrid DFT solutions with different Hartree-Fock exchange term (*w*) to elucidate possible errors of UNO UCCSD(T) approach.

Trial localized natural orbitals (ULO) by HDFT are indeed very important for effective and practical DLPNO CCSD(T_0) computations for open-shell clusters (Saitow et al. 2017). The ULO obtained by the localization of the canonical UNO by UB3LYP are used for trial orbitals in our DLPNO CCSD(T_0) computations as shown in Fig. S5. We have performed the normal (*n*-) PNO DLPNO CCSD(T_0) computations using trial ULOs obtained by UB3LYP (w = 20%), UB3LYP* (w = 15%), and UB3LYP*** (w = 10%) methods as shown in Fig. S5. Relative energies for each intermediate by *n*-PNO DLPNO CCSD(T_0) are not so different among trial ULOs employed because of the orbital optimization by the CC single (S) excitation process. This in turn means the ULOs by HDFT are useful for trial NOs for the DLPNO CCSD(T_0) computations. Therefore, UNO by UB3LYP (w = 20%) (see Fig. S1) has been used for practical trials for the ULO DLPNO CCSD(T_0) computations of total energies of possible intermediates in the Kok cycle for water oxidation in eq. (1) (Miyagawa et al. 2019, 2020, 2021, 2022a, b).

The DLPNO CCSD(T_0) computational results may be variable, depending on the sizes of the QM models employed. We have examined the environmental effects for the $CaMn_4O_6$ cluster on the basis of the QM I (103 atoms), QM II (227 atoms), and QM III (283 atoms) models. DLPNO CCSD(T_0) computations by using the ORCA program (Saitow et al. 2017) are time consuming in our in-house computer system (6TB memory/Xeon Platinum 8360HL 4CPUs machine). For example, the 2 ~ 3, 5 ~ 7, and 10 ~ 14 days are required for DLPNO CCSD(T_0) computations of QM I, II, and III models, respectively. Therefore, we have performed DLPNO CCSD(T_0) computations of possible intermediates in the S_i (i = 0 ~ 3) states to elucidate relative stabilities among them at the QM I model mainly constructed by amino acid residues in the first coordination shell. The QM II model involves the amino acid residues in the second coordination shell and several water molecules around the $CaMn_4O_6$ cluster. Therefore,

comparisons of the computational results for QM I and QM II models provide important information on the environmental effects.

Relative energies among the S_3 intermediates based on the QM II model were shown in Fig. 6 of the main text, indicating variations, depending on the weight (*w*) of the Hartree-Fock exchange term in the UB3LYP computations (*w* = 10 ~ 20 %). Therefore, DLPNO CCSD(T_0) results are crucial for elucidation of scope and reliability of HDFT results as shown in Fig. 6. The computational results based on the QM model I by DLPNO CCSD(T_0) are also shown for comparative discussions in Fig. S6 (Miyagawa et al. 2022b).

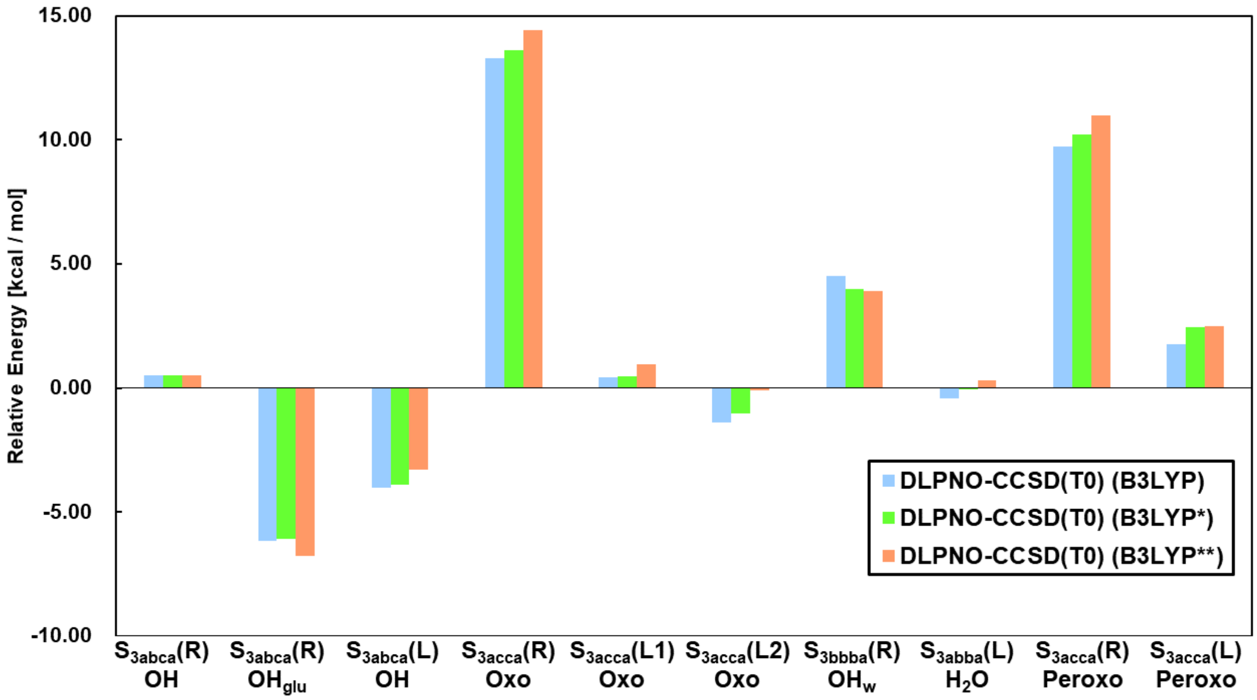

Fig. S5 Relative energies among the S_3 intermediates by *n*-DLPNO CCSD(T_0) computations using trial ULOs obtained by UB3LYP (*w* = 20%), UB3LYP* (*w* = 15%), and UB3LYP*** (*w* = 10%) methods (Miyagawa et al. 2022).

Relative stabilities among the S_3 intermediates based on sizes of the QM models

In this section, relative stabilities among the S_3 intermediates based on the small QM-I (103 atoms) are revisited as the references. DLPNO CCSD(T_0) computations are time-consuming for large systems. Therefore, our early computations are performed for the small QM-I (103 atoms) model to elucidate relative energies among possible S_3 intermediates (Miyagawa et al. 2022b) as shown in Figs. S5 and S6. However, the relative energies are considered to be sensitive to the QM models employed. In this review,

DLPNO-CCSD(T) based on the QM-II (227 atoms) model have been used to examine relative energies among the possible S_3 intermediates as shown in the main text. The full geometry optimizations of all the intermediates by the UB3LYP-D3 method have been performed before the DLPNO CCSD(T_0) computations. The key geometrical parameters obtained are summarized in Tables 4 and 5 in the main text. The DLPNO CCSD(T_0) computational results are different between QM-I (103 atoms) and QM-II (227 atoms) models, indicating importance of sizes of QM models employed. Detailed results are discussed in the main text.

We have examined scope and reliability of the conclusions based on QM II model by comparing with the computational results based on the QM III model (unpublished yet). The main conclusions for the S_3 state were not changed by the model expansion from QM II to QM III model. On the other hand, the ground spin states for the S_1 state were improved by the expansion from QM model II to QM III model in accord with the EPR results (Yamauchi et al. 1997). The QM-IV (350-380 atoms) models are in progress for confirmation of the computational results at the QM-III level. The QM-IV model is expected to provide important information on the water insertion pathway discussed in SI Part IV.

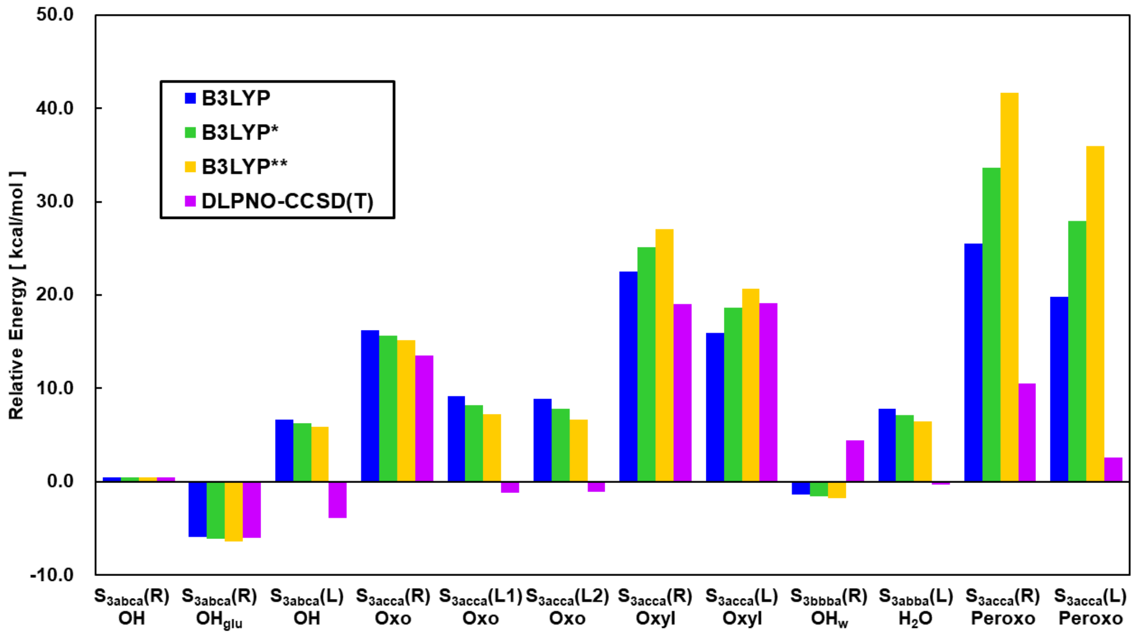

Fig. S6 Relative energy among the twelve S_3 intermediates by UB3LYP (w = 20%), UB3LYP* (w = 15%), UB3LYP** (w = 10%), and tight PNO (t-) DLPNO-CCSD(T) based on the QM-I (103 atoms) model (Miyagawa et al. 2022).

SI Part VII Possible reaction mechanisms for water oxidation via the multiple intermediates

model

Possible reactions modes for the native water oxidation in OEC of PSII

In this section, possible reaction pathways revealed by the interplay between theory and experiments are reviewed briefly. Recent SFX XFEL experiments (Kern et al. 2018; Suga et al. 2019; Hussein et al. 2021; Bhowmick et al. 2023) have elucidated the O-O bond formation in the $S_3 \rightarrow hv \rightarrow S_3$-Tyr-O• → [$S_4$] → S_0 transition. DLPNO CCSD(T_0) computations have provided crucial information for elucidating possible mechanisms for water oxidation in OEC of PSII. Interplay between theory and experiments at the present stage has elucidated five steps for the above transition: (a) generation of Tyr-O• radical, (b) generation of proton vacancy via proton release, (c) generation of the active precursor for the O-O bond formation, (d) the O-O bond formation, and (e) evolution of molecular oxygen and insertion of second water molecule. The timing of OET from the Mn-cluster to Tyr-O• radical in the (c) and (d) processes is very important, providing different mechanisms for water oxidation in OEC of PSII. Therefore, we may have three different cases on the theoretical grounds (Miyagawa et al. 2022b). To save the elongation of the main text, we here summarize the important information on several steps for water oxidation in OEC of PSII.

(1) The S_{3abca}(R)-OH is the most stable intermediate among the S_3 intermediates at the QM-II model. Thermal PCET for [S_{3abca}(R)-OH with the (4444) valence state] may produce partly the [S_{3acca}(R)-oxyl with the formal (4443)-O• valence state] at local high-temperature in the protein matrix. This may be a structural origin for the A mechanism in the text.

(2) Third flash for S_{3abca}(R)-OH generates the proton vacancy by releasing proton in an early state of the S_4 state, providing [S_{3abba}(R)-OH with the (4444) valence state + Tyr-O•]. After this step, several possibilities (A, B, C, and D) are conceivable on the theoretical grounds (Miyagawa et al. 2022b).

***Case I**: the O-O bond formation before OET to Tyr161-radical (mechanism A)*

(3) Proton release from [S_{3acca}(R)-oxyl with the formal (4443)-O• valence state] occurs, providing [S_{3abca}(R)-oxyl with the (4443)-O• valence + Tyr-O•] which undergoes the O-O bond formation, affording [S_{3abca}(R)-peroxide with the (3443) valence state + Tyr-O•] in the early S_4 state. This process is referred to as the mechanism A in the main text.

(4) [S_{3abca}(R)-peroxide with the (3443) valence state + Tyr-O•] undergoes the one electron transfer (OET) to Tyr-O•, providing [S_{4abca}(R)-peroxide with the (4443) valence state + Tyr-OH].

(5) [S_{4abca}(R)-peroxide with the (4443) valence state] undergoes the oxygen evolution to afford [S_{0abca}(R) (or S_{0accb}(R)) with the (3433) valence state + O_2] coupled with the concerted water insertion (CWI) (Shoji et al. 2018b) through the [S_{4abca}(R)-superoxide with the (3443) valence state].

Case II: the O-O bond formation coupled with OET to Tyr161-radical (mechanism B)

(6) [S_{3abca}(R)-oxyl with the (4443)-O• valence + Tyr-O•] undergoes the O-O bond formation coupled with the OET to Tyr-O•, namely non-adiabatic (NA) OET mechanism, providing [S_{4abca}(R)-peroxide with the (4443) valence state] for which the process (5) is followed for the oxygen evolution. The formation of the Mn(V) is not necessary for this mechanism.

(7) [S_{3abba}(R)-OH with (4444) + Tyr-O•] undergoes the O-OH bond formation coupled with the OET to Tyr-O•, namely Y_Z-assisted OOH mechanism, providing [S_{4abba}(R)-OOH with the (4443) valence state + Tyr-OH] followed by the proton transfer or PCET to provide Mn-peroxide with the (4443) valence state or Mn-superoxide [S_{4abca}(R)-OO• with (3443)]. S_{4abca}(R)-OO• undergoes the oxygen evolution followed by water insertion. This process is further discussed in relation to the reverse C*c*O process in the main text (Fig. 20) (see next section).

Case III: the O-O bond formation after OET to Tyr161-radical (mechanism C)

(8) The OET from [S_{3abba}(R)-OH with the (4444) valence state] to Tyr-O• provides the [formal high valent (HV) S_{4abca}(R)-oxo species with the (5444) valence state + Tyr-OH] and/or [S_{4abca}(R)-oxyl with the formal (4444)-O• valence state + Tyr-OH].

(9) The S_4 intermediate with strong HV S_{4abca}(R)-oxo (5444) character undergoes the O-O bond formation via the Ca-assisted concerted bond switching (CBS) mechanism (Shoji et al. 2019b), providing S_{4abca}(R)-peroxide with the (4443) valence state for which the process (5) is followed for the oxygen evolution. One electron reductions of the Mn_1(V) and Mn_4(IV) occur instead of the two-electron reduction from Mn_1(V) to Mn_1(III) even in the CBS process assisted by the Ca ion.

(10) The S_4 intermediate with strong S_{4abca}(R)-oxyl (4444)-O• character undergoes the O-O bond formation via the radical coupling (RC) mechanism, providing [S_{4abca}(R)-peroxide with the (4443) valence state] for which the process (5) is followed for the oxygen evolution.

Case III'; the O-O bond formation after OET to Tyr161-radical (mechanism D)

(11) The S_4 intermediate with strong HV S_{4abca}(R)-oxo (5444) character undergoes the O-OH bond formation via the Ca-assisted nucleophilic attack (NuA) of water molecule $H_2O_{(7)}$, providing [S_{4abca}(R)-hydroperoxide with the (4443)] valence state (Yamaguchi et al. 2019).

(12) The [S_{4abca}(R)-hydroperoxide with the (4443) valence state] undergoes the oxygen evolution via the proton-coupled (PC) electron transfer (ET) mechanism through the [S_{4acca}(R)-superoxide with the (3443) valence state], providing [S_{0abca}(R) (or S_{0accb}(R)) with the (3433) valence state + O_2].

Five different reaction pathways for the water oxidation

In this section, possible reaction mechanisms for water oxidation in OEC of PSII are summarized in

supporting Figures. The above classifications (Yamaguchi et al. 2019; Miyagawa et al. 2022b) have been applied to elucidate possible reaction mechanisms for water oxidation in OEC of PSII. The water oxidation processes in eqs. (9)-(12) are schematically illustrated in supporting Figs. S7-S11. Very recently, the TR SFX XFEL results (Bhowmick et al. 2023) have been presented for cyanobacteria, elucidating geometric structures of key intermediates in the $S_3 \rightarrow hv \rightarrow S_3$-Tyr-O• → $[S_4] \rightarrow S_0$ transition. One of the key findings by TR SFX XFEL is that the O-O bond formation and one electron reduction occur in the parallel manner in the S_4 state at room temperature. However, the transition structure (TS) for the O-O bond formation is not observed even by them because of very short lifetime, indicating the necessity of its location based on large QM models by HDFT/def2-TZVP level. Here, we summarize possible pathways, which can be proposed by the interplay between our QM and QM/MM computational results and available experimental results by other groups at the present time.

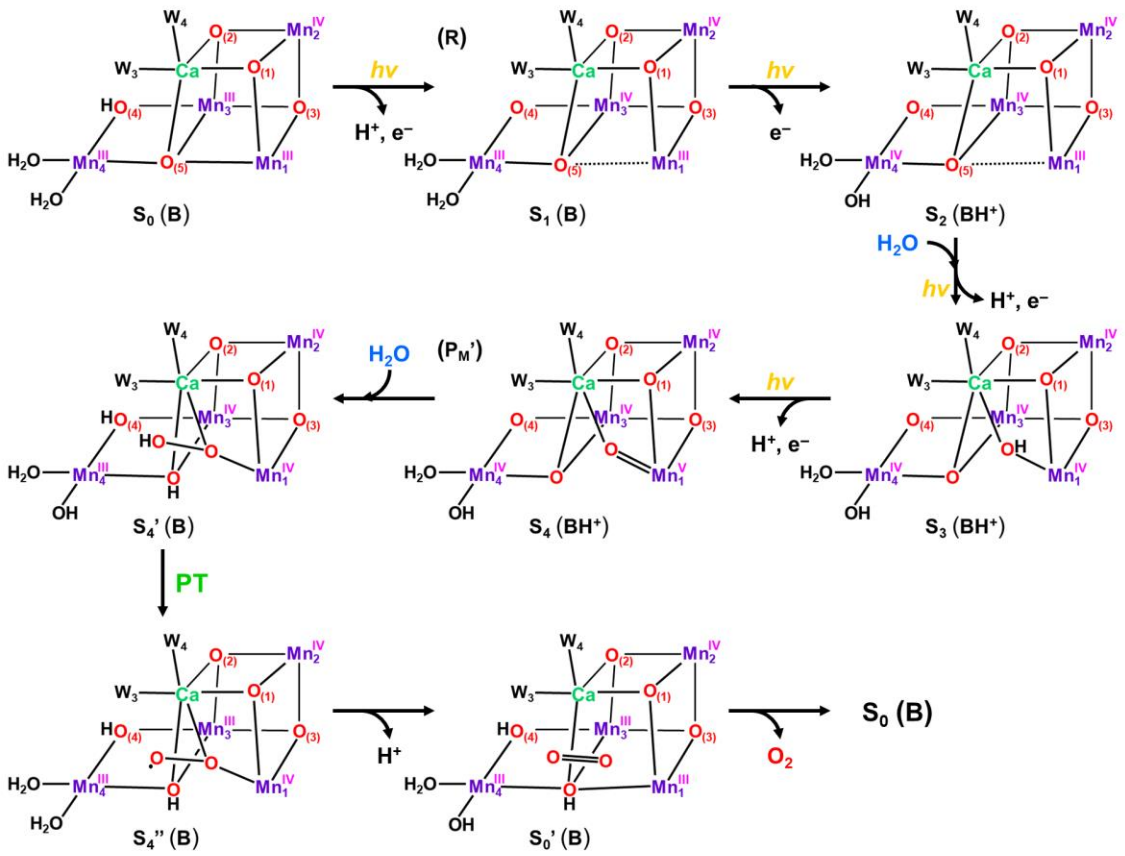

Fig. S7 Ca-assisted acid-base (AB) mechanism for the O-OH bond formation in the C-type reaction mode; ***Case III'*** (Yamaguchi et al. 2019). Formal formation of the Ca-assisted Mn(V)=$O_{(6)}$ bond is assumed in this mechanism (mechanism D). Rapid OOH bond formation is necessary before 1200 μs after the third flash (Bhowmick et al. 2023). The reductions from $Mn(V)_1$ to $Mn(IV)_1$ and $Mn(IV)_4$ to $Mn(III)_4$ occur in the course of the OOH bond formation, indicating two one-electron reductions at the

final stage instead of the simple AB mechanism. Peroxide is not formed in in this mechanism, providing Mn-superoxide radical directly. Ligation of the Asp170 to the Ca ion is broken to insert second water $H_2O_{(7)}$ (Bhowmick et al. 2023) because of the maximum eight coordination number of the Ca(II) ion.

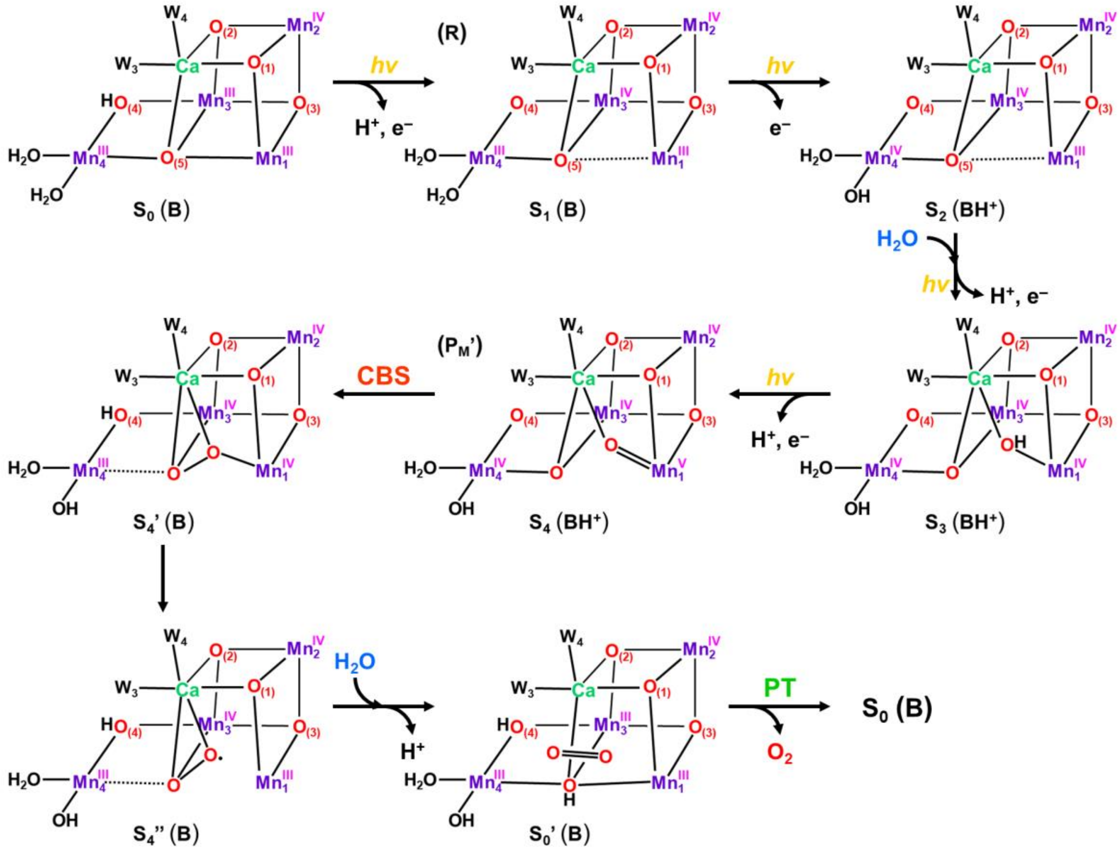

Fig. S8 Ca-assisted concerted bond switching (CBS) mechanism for the O-O bond formation in the C-type reaction mode (Shoji et al. 2019b). Formal formation of the Mn(V)=$O_{(6)}$ bond is assumed in this mechanism (timing ***Case III***). Rapid OO bond formation via the CBS mechanism is necessary before 1200 μs after the third flash (Bhowmick et al. 2023). The reductions from $Mn(V)_1$ to $Mn(IV)_1$ and $Mn(IV)_4$ to $Mn(III)_4$ occur in the course of the O-O bond formation, indicating two one-electron reductions at the final stage instead of the simple AB mechanism. Peroxide is formed in in this mechanism, indicating two step mechanism. Ligation of the Asp170 to the Ca ion is broken to insert second water $H_2O_{(7)}$ which undergoes substitution reaction with superoxide anion.

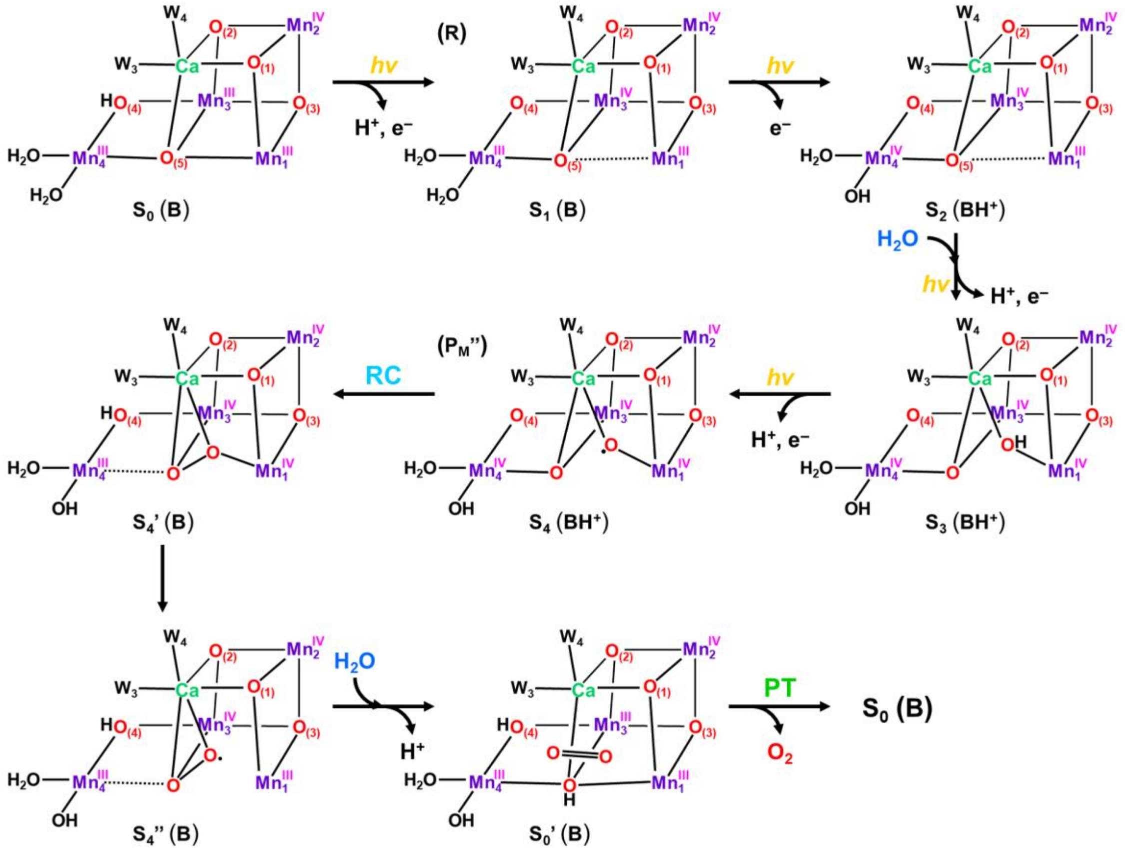

Fig. S9 Radical coupling (RC) mechanism for the O-O bond formation in the C-type reaction mode (***Case III***). Formation of the Ca-assisted Mn(V)=$O_{(6)}$ bond is not necessary in this mechanism (Yamaguchi et al. 2010) because of the formation of the spin polarization configuration Mn(IV)-O• (Saito et al. 2012). Rapid OO bond formation via the radical coupling (RC) mechanism is necessary before 1200 μs after the third flash (Bhowmick et al. 2023). The reductions from $Mn(V)_1$ to $Mn(IV)_1$ and $Mn(IV)_4$ to $Mn(III)_4$ occur, indicating two one-electron reductions at the final stage. Peroxide is formed in in this mechanism, indicating two step mechanism. Ligation of the Asp170 to the Ca ion is broken to insert second water $H_2O_{(7)}$ which undergoes substitution reaction with superoxide anion.

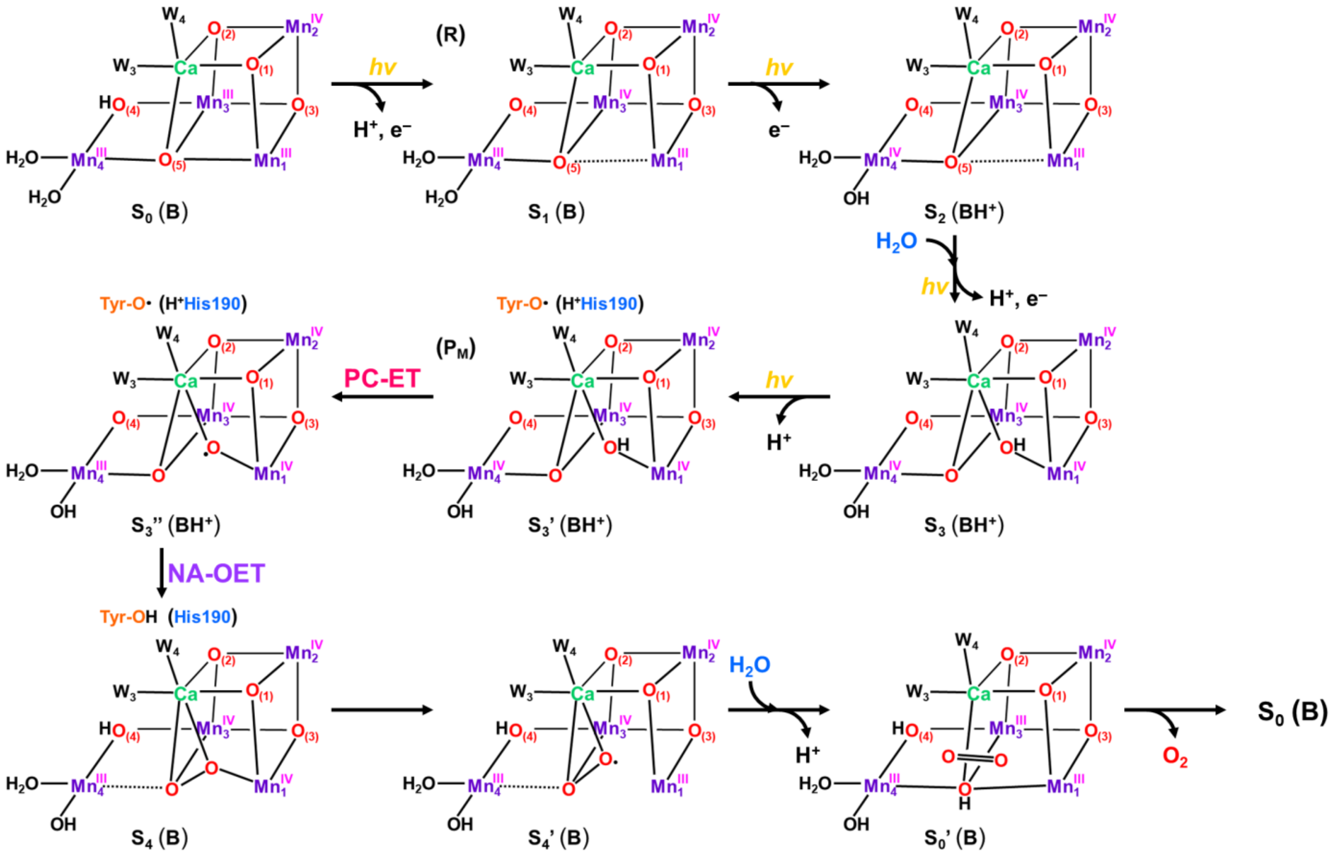

Fig. S10 Y_Z-assisted non-adiabatic (NA) one-electron-transfer (OET) mechanism (Shoji et al. 2018a) for the O-O bond formation in the B-type reaction mode (***Case II***). Formal formation of the $Mn(V)=O_{(6)}$ bond is not assumed because Tyr161-radical acts as one electron acceptor in this mechanism. The O-O bond formation is coupled with the one electron reduction of Tyr161-radical in this mechanism, which is accord with TR SFX XFEL results (Bhowmick et al. 2023). Peroxide is formed in in this mechanism, indicating two step mechanism. Ligation of the Asp170 to the Ca ion is broken to insert second water $H_2O_{(7)}$ which undergoes substitution reaction with superoxide anion. The one electron reductions occur at $Mn(IV)_4$ and tyr161-radical sites occur for the O-OH bond formation in the NA-OET mechanism.

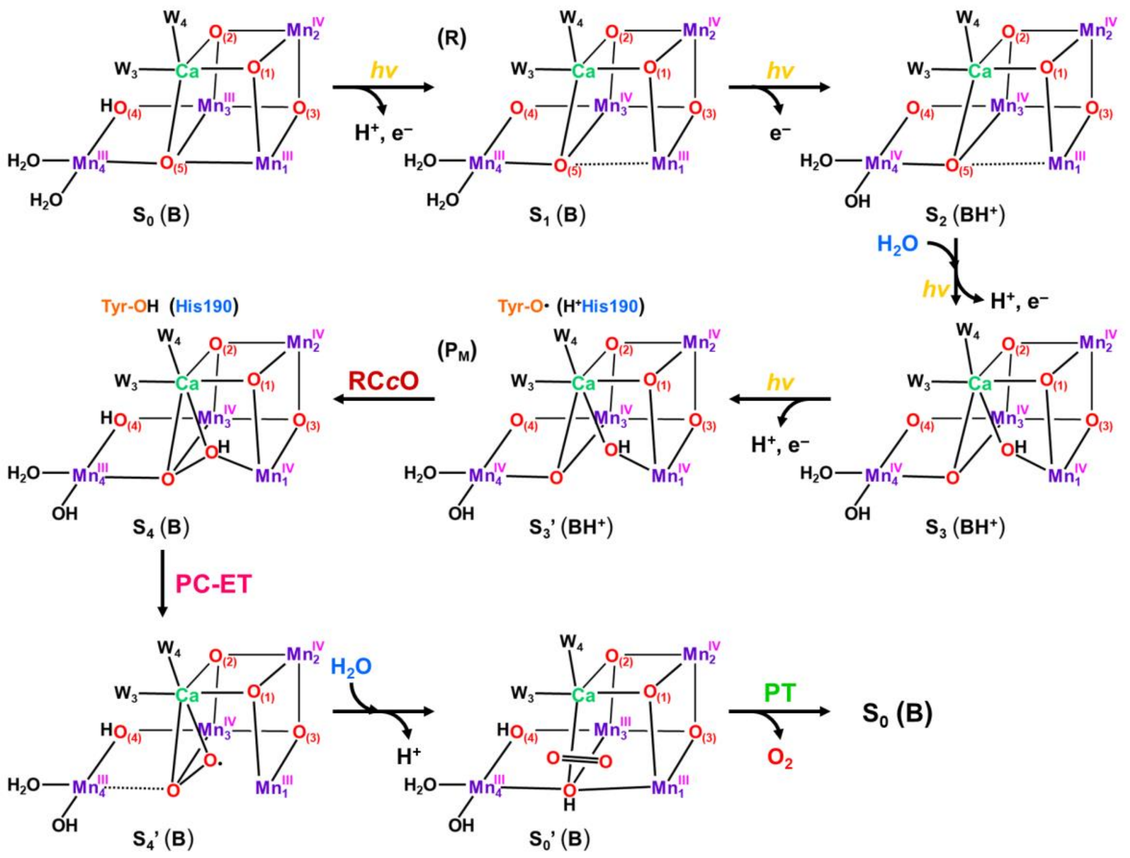

Fig. S11 Y_Z-assisted hydroperoxide (HP) mechanism for the O-OH bond formation. Formal formation of the Mn(V)=$O_{(6)}$ bond is not assumed because Tyr-O radical acts as one electron acceptor in this mechanism. The O-OH bond formation is coupled with the one electron reduction of Tyr-O radical in this mechanism, which is accord with the TR SFX XFEL results (Bhowmick et al. 2023). Ligation of the Asp170 to the Ca ion is broken to insert second water $H_2O_{(7)}$ because of the maximum eight coordination number of the Ca(II) ion. Y_z-assisted adiabatic OET mechanism for hydroperoxide formation is regarded as the reverse process of oxygen reduction by C*c*O as illustrated in Fig. 20 in the main text, indicating the reverse C*c*O (RC*c*O) mechanism. The spin inversion is not necessary for this adiabatic process. The one electron reductions occur at Mn(IV)$_4$ and Tyr161-O radical sites for the O-OH bond formation in the Y_Z-assisted adiabatic (A)-OET mechanism. Proton-coupled electron-transfer (PC-ET) plays an important role in this mechanism. Therefore, a continuous process via RC*c*O-PC-ET is regarded as the PC-ET mechanism for the O-O bond formation.

Thus, at the moment, the experimental finding (Bhowmick et al. 2023) can be explained by the NA-OET mechanism in Fig. S10 and/or Y_Z-assisted hydroperoxide mechanism (reverse C*c*O: RC*c*O) in Fig. S11) mechanism as discussed in the main text.

SI Part VIII Formal reverse analogy of cytochrome *c* oxidase (C*c*O) to PSII

Structure of cytochrome *c* oxidase (C*c*O)

In this section, RC*c*O mechanism in Fig. 20 in the main text is explained in detail. Cytochrome *c* oxidase (C*c*O) is consisted of four redox-active transition metal ions (Cu_A, Fe_a, Fe_{a3}, and Cu_B). C*c*O catalyzes reverse reaction of water oxidation in eq. (1), namely reduction of molecular oxygen into two water molecules: $O_2 + 4H^+ + 4e^- \rightarrow 2H_2O$ (Yoshioka et al. 2003). Therefore, formal reverse analogy is conceivable between PSII and C*c*O from the viewpoint of the functional behaviors although the constructions of the reaction sites are different between them. Judging from available experimental results, Heme Fe_{a3} and Cu_B sites of C*c*O for oxygen reduction are regarded as the $Mn_{4(a)}$ and $Mn_{1(d)}$ sites of PSII for water oxidation as shown in Fig. 20 in the main text. Similarly, Tyr244 and His240 of C*c*O are regarded as Tyr161 and His190 of PSII, exhibiting an important proton-coupled electron transfer (PCET) reaction via a water molecule coupled with Tyr244 (Yoshioka et al. 2003). Three proton insertion pathways, K-, D-, and H-pathways, have been proposed for C*c*O, whereas three proton release pathways: path I (Cl1channel), path II (T_Z-channel) and path III (O_4-channel), have been proposed for PSII. The oxygen insertion and water release pathways are also proposed for C*c*O. On the other hand, oxygen release and water input pathways are proposed for PSII. Thus, C*c*O and PSII have biomolecular systems structures for oxygen (four electron) reduction and water (four electron) oxidation, respectively. Sun energy is converted into the chemical energy of NADPH (NADH) in photosynthesis, whereas the chemical energy of NADPH(NADH) is used for oxygen reduction coupled with proton transfers in C*c*O.

Possible roles of Tyr244 of C*c*O and Tyr161 of PSII

In this section, analogy between Tyr161 in OEC of PSII and Tyr244 in C*c*O is examined in detail. The initial state (Reactant state; R) of the oxygen reduction in C*c*O is expressed as

$$R: \quad Fe(II)_{a3}\ldots Cu(I)_B + \text{Tyr244-OH} \qquad S_0 \qquad \text{(s16a)}$$

where S_0 denotes the corresponding state of the Kok cycle for water oxidation. Molecular oxygen is inserted into the R-state, providing the A state of C*c*O, which is an exchange coupled state between Fe(II) and O_2. The A' state with superoxide anion is formed by the one electron transfer (OET) from $Fe(II)_{a3}$ to O-O. Further OET from $Cu(I)_B$ to the O-O anion provides the A'' state with oxygen dianion, which is protonated to afford hydroperoxide anion coordinated to $Fe(III)_{a3}$, namely A''' state.

A:	$Fe(II)_{a3}$-O-O…$Cu(I)_B$ + Tyr244-OH	S_{4f}	(s17a)
A':	$Fe(III)_{a3}$-O-O•…$Cu(I)_B$ + Tyr244-OH	S_{4c}	(s18a)
A'':	$Fe(III)_{a3}$-O-O^-…$Cu(II)_B$ + Tyr244-OH	S_{4b}	(s19a)
A''':	$Fe(II)_{a3}$-O-OH…$Cu(II)_B$ + Tyr244-O^-	S_{4a}	(s20a)

The key step for oxygen reduction in C*c*O is the O-OH bond dissociation step (P-sate) by OET from Tyr244O-O anion for heterolysis of the OOH bond of A''', generating the P_M state.

P_M:	[$Fe(IV)_{a3}$=O^{2+} $^-$HO-$Cu(II)_B$] + Tyr244-O•	S_3-Tyr161-O•	(s21a)
P_M':	$Fe(V)_{a3}$=O^{3+} $^-$HO-$Cu(II)_B$] + Tyr244-O^-	S_{4d}	(s22a)
F:	$Fe(IV)_{a3}$=O^{2+} $^-$HO-$Cu(II)_B$] + Tyr244-OH	S_3	(s23)

The formation of $Fe(V)_{a3}$=O^{3+} bond is not necessary if the Tyr244-O• radical is participated for the O-OH dissociation reaction as shown in eq. (s21). On the other hand, the formal high-valent $Fe(V)_{a3}$=O^{3+} bond is formed is under no participation of Tyr244-O• radical as shown in eq. (s22a). The one electron reduction of Tyr244-O• in the PM state provides the F state with Tyr244-OH. Therefore, four electron reduction of molecular oxygen occurs by using two electron from $Fe(II)_{a3}$, one electron from $Cu(I)_A$ and one electron from Tyr244 in the Tyr244-radical assisted O-O dissociation of C*c*O. Participation of $Fe(II)_a$ might be possible to avoid two electron process on $Fe(II)_{a3}$.

The above oxygen reduction reaction by C*c*O is regarded as a reverse process of oxygen evolution in OEC of PSII. The key state for the O-OH bond formation of PSII is the reverse P_M (RP) state, namely S_3-Tyr161-O• state:

S_3-Tyr161-O•:	[$Mn(IV)_4$=O^{2+} $^-$HO-$Mn(IV)_1$ + Tyr161-O•]	(s21b)
S_{4d}:	[$Mn(V)_4$=O^{3+} $^-$HO-$Mn(IV)_1$ + Tyr161-OH].	(s22b)

The formation of the formal high-valent Mn(V) site is not necessary for S_3-Tyr161-O•. On the other hand, Mn(V)=O^{3+} may be formed by early PCET process before the O-O bond formation under no participation of Tyr161-O•: S_{4d}-state. The O-OH bond formation (Y_Z-OOH) assisted by Tyr161-O•-radical is feasible for S3-Tyr161-O•, providing hydroperoxide anion S_{4a}, for which proton shift to Tyr244-O^- is feasible to afford peroxide dianion S_{4b}, and successive OET from peroxide dianion to Cu(II) affords the superoxide anion S_{4c}. The formal Mn(IV) to Mn(II) reduction process is conceivable as the reverse C*c*O process, providing the S_0 state with the (3442) valence configuration. However, $Mn(IV)_3$ site is also involved in the one electron reduction process as shown in the S_{4c}' and S_0', providing the (3433) valence state.

S_{4a}:	$[Mn(III)_4$-O-OH $Mn(IV)_1]$ + Tyr244-O^-	(s20b)
S_{4b}:	$[Mn(III)_4$-O-O^- $Mn(IV)_1]$ + Tyr244-OH	(s19b)
S_{4c}:	$[Mn(III)_4$-O-O• $Mn(III)_1]$ + Tyr244-OH	(s18b)
S_0:	$[Mn(II)_4$ •O-O• $Mn(III)_1]$ + Tyr244-OH	(s18b)
S_{4c}':	$[Mn(III)_4Mn(IV)_3$-O-O• $Mn(III)_1]$ + Tyr244-OH	(s18c)
S_{4c}':	$[Mn(III)_4Mn(III)_3$ •O-O• $Mn(III)_1]$ + Tyr244-OH	(s17c)
S_0':	$[Mn(III)_4Mn(III)_3…Cu(I)_B]$ + Tyr244-OH	(s16c)

Thus, Tyr-O radical plays an important role for the O-OH (or OO) bond formation in accord with the experimental finding (Bhowmick et al. 2023) that the O-O bond formation and reduction of Tyr161-radical occur in a parallel manner in the case of the S_4 state of the Kok cycle of cyanobacteria.

SI Part IX Future prospect

QM/MM/MD computations and entropy effects

In this section, the entropy term for proton transfer is touched briefly. We have performed the QM and QM/MM computations of the model complexes of the OEC of PSII to elucidate relative energies among several possible intermediates in the S_i state (i = 0 ~ 4) of the Kok cycle as shown in Fig. S12. However, relative stabilities among them might be variable on the basis of the entropy effect at finite temperature (Capone et al. 2020; Greife et al. 2023). In fact, relative stability between the Mn-oxyl/oxo and Mn-hydroxide intermediates in the S_3 state may be variable, depending on the temperature as mentioned in the main text. Therefore, we have proposed an equilibrium model for the S_3 intermediates (Renger 2012; Isobe at al. 2016). Recent TR XFEL SFX at room temperature (Bhowmick et al. 2023) and FTIR (Greife et al. 2023) results have elucidated multi-step dynamical mechanisms of the O-O bond formation in the S_4 state. In our review article in 2015 (Shoji et al. 2015a), we have also emphasized an important role of proton dynamics in OEC of PSII as shown in Fig. S12 in accord with the referee's comment. Therefore, the QM/MM/MD computations are really desirable for theoretical investigations of the dynamical mechanisms for the native water oxidation in combinations of the TR XFEL SFX, FTIR, and other experiments as illustrated in Fig. S12. However, the QM/MM/MD investigations are still in future task of our theoretical group.

In 1976, the electron-transfer (ET) diradical model (Takabe and Yamaguchi 1976) was proposed to electron transfer (ET) reactions strongly coupled with molecular deformations of reactants in

addition to deformations of environments of the original Marcus ET theory (Marcus 1956). Indeed, our BS ET approach based on the QM/MM model (Shoji et al. 2015) was applicable to the NA OET mechanism of the O-O bond formation for water oxidation (Shoji et al. 2018a). The proton-transfer process is formally regarded as an ionic process as shown in eq. (s24a). On the other hand, the one ET process is formally regarded as a valence variation (redox active) process as shown in eq. (s24b). The BS ET model (Takabe and Yamaguchi 1976) is applicable to describe the coupling of these processes, namely the concerted proton coupled (PC) electron transfer (ET) process as shown in eq. (s24c) (Weinberg et al. 2012), where moderate ET diradical character is emerged at TS. However, extension of the BS ET method to elucidate the entropy contribution to the PCET in our theoretical model is a future problem in our group.

$$M(X)\text{-}OH_2 + O{=}M(Y) \rightarrow M(X)\text{-}OH^- + {}^+HO{=}M(Y) \quad (s24a)$$

$$M(X)\text{-}OH_2 + O{=}M(Y) \rightarrow M(X+1)\text{-}OH_2 + O{=}M(Y-1) \quad (s24b)$$

$$M(X)\text{-}OH_2 + O{=}M(Y) \rightarrow M(X+\delta)\text{-}OH\ldots H\ldots O{=}M(Y-\delta) \rightarrow M(X+1)\text{-}OH + HO\text{-}M(Y-1) \quad (s24c)$$

Classical and quantum natures of proton transfer

In this final section, the quantum nature of proton is touched briefly. Over past decades, the quantum nature of proton in biological systems has been investigated with several theoretical models (Marx et al. 1999; Curchod and Martinez 2018; Crespo-Otero and Barbatti 2018). The activation barriers for proton transfer reactions calculated by the BS HDFT methods are often larger than the experimental values, suggesting possible roles of quantum tunneling effects of proton (Shoji et al. 2018a). In fact, the classical and quantum nature of proton and electron transfers has been investigated in several systems such as the Eigen complex ($H_9O_4^+$) (Moser et al. 1992; Marx et al. 1999). The Born-Oppenheimer approximation (BOA) decoupling the electron and nuclear motions may break down in the non-adiabatic PC-ET processes in biological system (Shigeta et al. 1999). However, theoretical investigations of the quantum nature of the PC-ET processes in the hydrogen bonding networks around the $CaMn_4O_6$ cluster have not been performed yet because of several reasons. For example, one of the reasons is that the QM/MM/MD model for OEC of PSII becomes large because the $CaMn_4O_x$ (x = 5, 6) cluster is strongly coupled with the hydrogen bonding networks (at least 57 water molecules around the cluster in our QM/MM model) in the OEC of PSII as shown in Figs. 14-18 (Shoji et al. 2015). Therefore, ab initio quantum molecular dynamics investigation of water oxidation in OEC of PSII is a future interesting and important problem in photosynthesis as shown in Fig. S12.

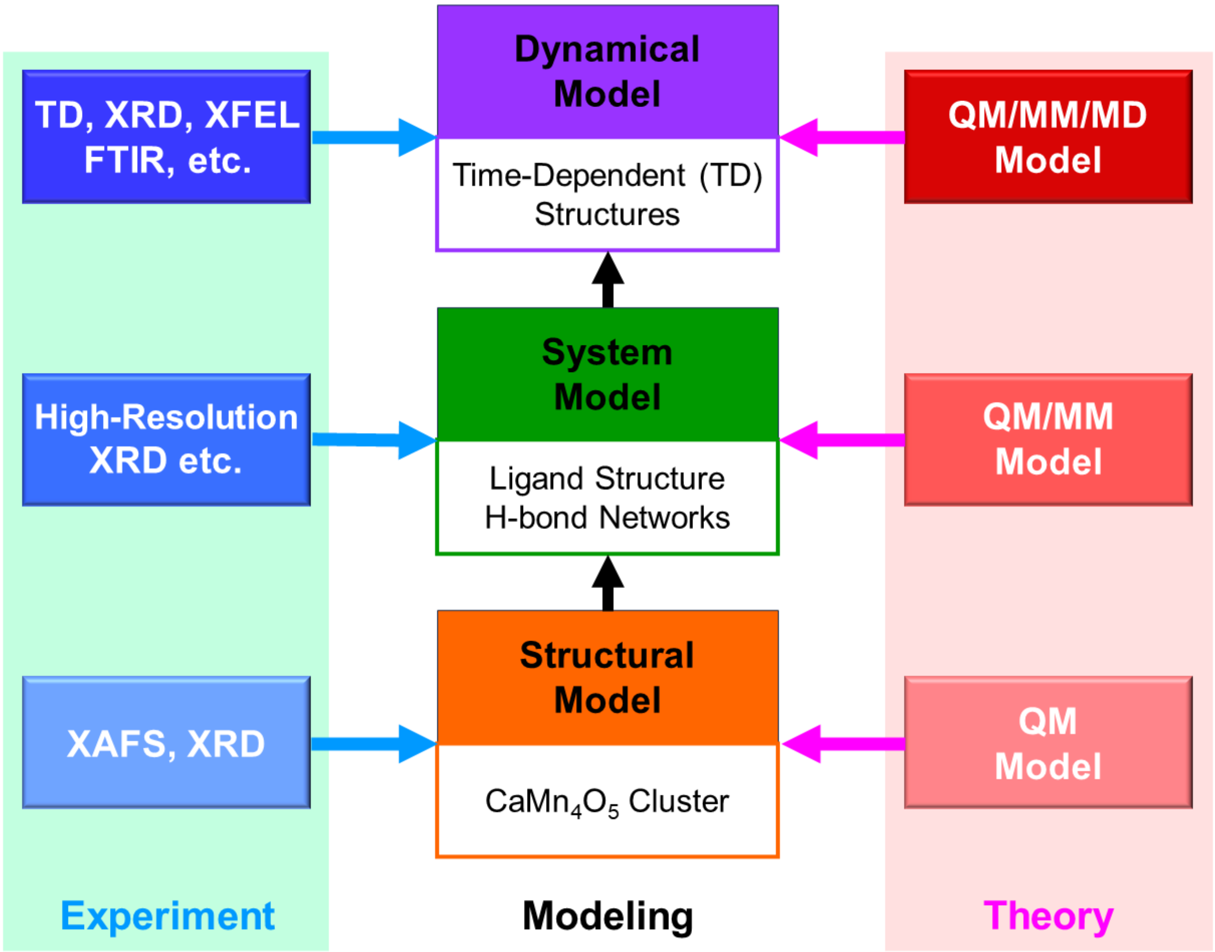

Fig. S12 Three levels of mechanisms of water oxidation in the oxygen-evolving complex (OEC) of photosystem II (PSII) (Shoji et al. 2015a). In this review, we have mainly summarized our QM and QM/MM computational results in combinations with available experimental results. The QM/MM/MD computations are in progress in our group. Many QM/MM/MD computational results for OEC of PSII by other groups are not reviewed in this paper.
